# Deterministic Evolutionary Trajectories Influence Primary Tumor Growth: TRACERx Renal

**DOI:** 10.1016/j.cell.2018.03.043

**Published:** 2018-04-19

**Authors:** Samra Turajlic, Hang Xu, Kevin Litchfield, Andrew Rowan, Stuart Horswell, Tim Chambers, Tim O’Brien, Jose I. Lopez, Thomas B.K. Watkins, David Nicol, Mark Stares, Ben Challacombe, Steve Hazell, Ashish Chandra, Thomas J. Mitchell, Lewis Au, Claudia Eichler-Jonsson, Faiz Jabbar, Aspasia Soultati, Simon Chowdhury, Sarah Rudman, Joanna Lynch, Archana Fernando, Gordon Stamp, Emma Nye, Aengus Stewart, Wei Xing, Jonathan C. Smith, Mickael Escudero, Adam Huffman, Nik Matthews, Greg Elgar, Ben Phillimore, Marta Costa, Sharmin Begum, Sophia Ward, Max Salm, Stefan Boeing, Rosalie Fisher, Lavinia Spain, Carolina Navas, Eva Grönroos, Sebastijan Hobor, Sarkhara Sharma, Ismaeel Aurangzeb, Sharanpreet Lall, Alexander Polson, Mary Varia, Catherine Horsfield, Nicos Fotiadis, Lisa Pickering, Roland F. Schwarz, Bruno Silva, Javier Herrero, Nick M. Luscombe, Mariam Jamal-Hanjani, Rachel Rosenthal, Nicolai J. Birkbak, Gareth A. Wilson, Orsolya Pipek, Dezso Ribli, Marcin Krzystanek, Istvan Csabai, Zoltan Szallasi, Martin Gore, Nicholas McGranahan, Peter Van Loo, Peter Campbell, James Larkin, Charles Swanton, the TRACERx Renal Consortium

**Affiliations:** 1 Translational Cancer Therapeutics Laboratory, the Francis Crick Institute, London NW1 1AT, UK; 2 Renal and Skin Units, the Royal Marsden NHS Foundation Trust, London SW3 6JJ, UK; 3 Department of Bioinformatics and Biostatistics, the Francis Crick Institute, London NW1 1AT, UK; 4 Urology Centre, Guy’s and St. Thomas’ NHS Foundation Trust, London SE1 9RT, UK; 5 Department of Pathology, Cruces University Hospital, Biocruces Institute, University of the Basque Country, Barakaldo, Spain; 6 Department of Urology, the Royal Marsden NHS Foundation Trust, London SW3 6JJ, UK; 7 Department of Pathology, the Royal Marsden NHS Foundation Trust, London SW3 6JJ, UK; 8 Department of Pathology, Guy’s and St. Thomas’ NHS Foundation Trust, London SE1 7EH, UK; 9 Cancer Genome Project, Wellcome Trust Sanger Institute, Hinxton CB10 1SA, UK; 10 Department of Surgery, Addenbrooke’s Hospitals NHS Foundation Trust, Cambridge CB2 0QQ, UK; 11 Department of Medical Oncology, Guy’s and St. Thomas’ NHS Foundation Trust, London SE1 9RT, UK; 12 Experimental Histopathology Laboratory, the Francis Crick Institute, London NW1 1AT, UK; 13 Department of Scientific Computing, the Francis Crick Institute, London NW1 1AT, UK; 14 Advanced Sequencing Facility, the Francis Crick Institute, London NW1 1AT, UK; 15 Department of Radiology, the Royal Marsden NHS Foundation Trust, London SW3 6JJ, UK; 16 Berlin Institute for Medical Systems Biology, Max Delbrueck Center for Molecular Medicine, Berlin, Germany; 17 Bill Lyons Informatics Centre, UCL Cancer Institute, University College London, London WC1E 6DD, UK; 18 Bioinformatics and Computational Biology Laboratory, the Francis Crick Institute, London NW1 1AT, UK; 19 Cancer Research UK Lung Cancer Centre of Excellence London, University College London Cancer Institute, London WC1E 6DD, UK; 20 Department of Physics of Complex Systems, ELTE Eötvös Loránd University, Budapest, Hungary; 21 Department of Bio and Health Informatics, Technical University of Denmark, Kgs Lyngby 2800, Denmark; 22 Computational Health Informatics Program, Boston Children’s Hospital, Harvard Medical School, Boston, MA, USA; 23 Cancer Genomics Laboratory, the Francis Crick Institute, London NW1 1AT, UK; 24 Department of Human Genetics, University of Leuven, 3000 Leuven, Belgium; 25 Department of Medical Oncology, University College London Hospitals, London NW1 2BU, UK

**Keywords:** renal cell cancer, cancer evolution, intratumor heterogeneity, metastasis, tumor diversity, deterministic evolution, chromosome instability, punctuated evolution, branched evolution, linear evolution

## Abstract

The evolutionary features of clear-cell renal cell carcinoma (ccRCC) have not been systematically studied to date. We analyzed 1,206 primary tumor regions from 101 patients recruited into the multi-center prospective study, TRACERx Renal. We observe up to 30 driver events per tumor and show that subclonal diversification is associated with known prognostic parameters. By resolving the patterns of driver event ordering, co-occurrence, and mutual exclusivity at clone level, we show the deterministic nature of clonal evolution. ccRCC can be grouped into seven evolutionary subtypes, ranging from tumors characterized by early fixation of multiple mutational and copy number drivers and rapid metastases to highly branched tumors with >10 subclonal drivers and extensive parallel evolution associated with attenuated progression. We identify genetic diversity and chromosomal complexity as determinants of patient outcome. Our insights reconcile the variable clinical behavior of ccRCC and suggest evolutionary potential as a biomarker for both intervention and surveillance.

## Highlights

• ccRCC evolutionary subtypes correlate with clinical phenotypes
• Genetic diversity and chromosome complexity contribute to patient outcomes
• Early fixation of multiple driver events leads to rapid growth and metastases
• Subclonal diversification is linked with slower growth and attenuated metastases

## Graphical Abstract

**Figure:**
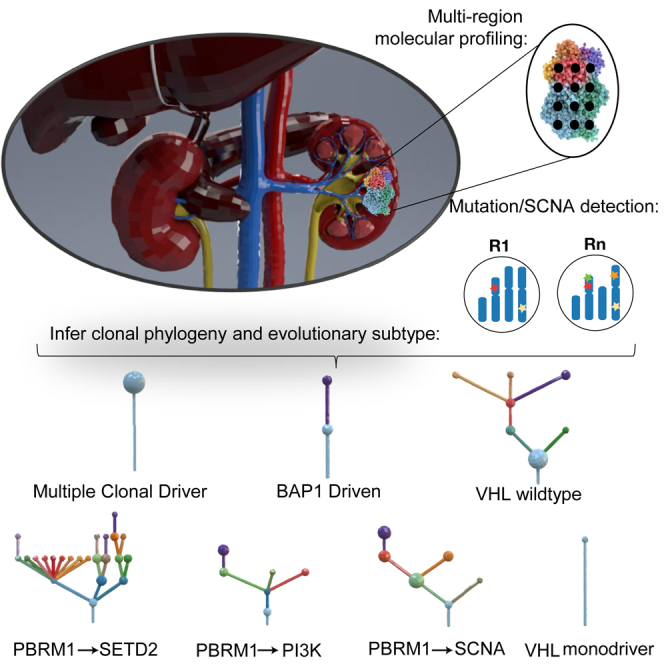

## Teaser

A multi-center prospective study on 101 patients with clear-cell renal cell carcinoma resolves the evolutionary features and subtypes underpinning the diverse clinical phenotypes of the disease and suggests these features as potential biomarkers for guiding intervention and surveillance.

## Introduction

Renal cell carcinoma (RCC) is the 7^th^ most frequently diagnosed malignancy (Znaor et al., 2015), with a rising incidence in the developed world (Smittenaar et al., 2016). The most common histological subtype, clear cell (ccRCC), is associated with a wide range of clinical outcomes. Around one-third of patients with localized ccRCC relapse following surgery, with tumor size, grade, and necrosis (Leibovich et al., 2003), the presence of vascular and/or fat invasion (da Costa et al., 2012), and sarcomatoid differentiation (Zhang et al., 2015) shown to impact the risk of recurrence. While these parameters are useful for patient counselling and stratification for follow-up and adjuvant studies, their predictive accuracy is inexact. Solitary metastasis at relapse may be amenable to surgery (metastasectomy) or local therapy (e.g., ablation) on a case-by-case basis (Bex et al., 2016). Patients relapsing with multiple but low volume, slow-growing metastases could be observed initially, but the risk of deferring systemic therapy remains unclear (Rini et al., 2016). Up to 30% of patients present with metastatic disease at the outset. In select cases, primary surgery is still used with cytoreductive intent; while some patients will also undergo a complete metastasectomy with curative intent. Patient selection for these interventions remains under intense debate, as does the management of small renal masses (SRMs) (renal lesions <4 cm in size). Increasing use of abdominal cross-sectional imaging has led to incidental discovery of SRMs, the majority of which have favorable natural history, leading to concerns about over-treatment (Welch et al., 2017). At present, molecular profiling does not impact decision-making in any of these clinical scenarios.

The molecular landscape of ccRCC was elucidated by a number of next-generation sequencing studies (Cancer Genome Atlas Research Network, 2013, Dalgliesh et al., 2010, Sato et al., 2013, Scelo et al., 2014, Varela et al., 2011) that revealed frequent inactivation of the *VHL* tumor suppressor gene, alterations in the SWI/SNF complex (Varela et al., 2011), histone-modifying genes (Dalgliesh et al., 2010), and the PI3K/AKT/mTOR pathway (Cancer Genome Atlas Research Network, 2013, Sato et al., 2013, Scelo et al., 2014). Recurrent arm level or focal losses are observed on chromosomes 1p, 3p, 4q, 6q, 8p, 9p, and 14q, and gains on chromosomes 1q, 2q, 5q, 7q, 8q, 12p, and 20q (Beroukhim et al., 2009, Cancer Genome Atlas Research Network, 2013). We previously reported significant mutational and somatic copy number alteration (SCNA) intratumor heterogeneity (ITH) in ten cases of advanced ccRCC (Gerlinger et al., 2014, Martinez et al., 2013), showing that single-biopsy analyses may miss important genetic events or misclassify them as clonal due to the “illusion of clonality,” thus hindering our understanding of tumor evolution. To date, attempts to molecularly classify ccRCC have included single biopsy analyses of mutations (Hakimi et al., 2013, Kapur et al., 2013, Sato et al., 2013) or gene expression and methylation (Cancer Genome Atlas Research Network, 2013, Chen et al., 2016).

To aid an evolutionary classification of RCC, we established a multi-center prospective longitudinal cohort study, Tracking Renal Cell Cancer Evolution through therapy (TRACERx Renal, https://clinicaltrials.gov/ct2/show/NCT03226886), with a protocol-specified endpoint of examining the association of ITH with disease stage and clinical outcomes through multi-region genomic profiling of primary tumors (Turajlic and Swanton, 2017). The TRACERx Renal program began recruitment in July 2012, enrolling patients undergoing nephrectomy (with curative or cytoreductive intent) for suspected or confirmed renal cell carcinoma (STAR Methods), with a target accrual of 320 patients with ccRCC. We report our interim findings of the patterns of ITH, clonal evolution, and tumor progression in the first 101 patients with the diagnosis of clear cell non-familial RCC (for full inclusion criteria for this cohort see STAR Methods).

## Results

### Intratumor Heterogeneity of Driver Events in Primary ccRCC

**Figure S1:**
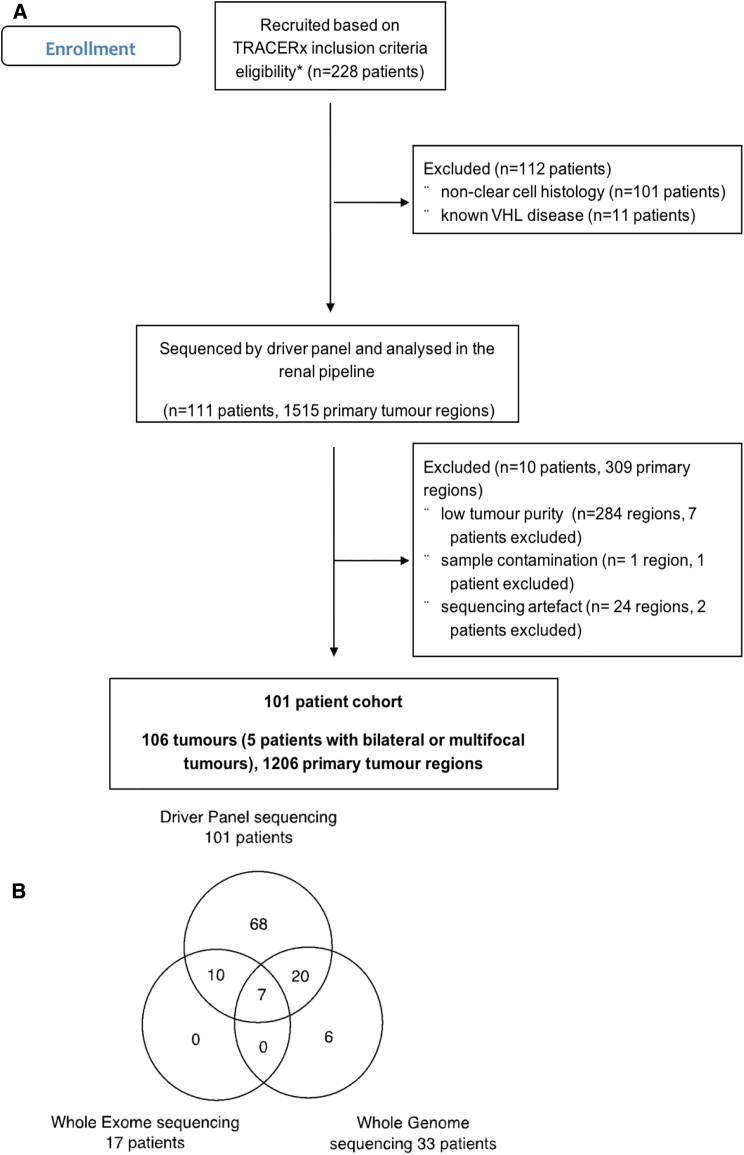
Consort Diagram, Related to STAR Methods (A and B) (A) shows the Consort diagram for the filtering steps leading to the reported cohort; (B) shows the summary of Driver Panel, Whole Exome and Whole Genome Sequencing in the TRACERx Renal 101 Cohort.

Clinical annotation of the 101 patients under study is provided in Table S1. Demographic and stage distribution were consistent with the referral patterns of the participating centers. All the samples were profiled using a bespoke sequencing panel targeting ∼110 putative ccRCC driver genes (Figure S1A and STAR Methods, Driver Panel). This approach enabled us to maximize the sequencing depth, a critical factor for correctly inferring evolutionary trajectories (Noorbakhsh and Chuang, 2017). Single nucleotide variants (SNVs), dinucleotides variants (DNVs), small insertion and deletions (INDELs), and SCNAs were successfully derived from 1,206 tumor regions across 106 primary tumors (median 7 [range, 3–75] regions per tumor) from 101 patients, as five patients donated pairs of primary tumors. Within the same cohort, 107 regions from 17 tumors were profiled by whole exome sequencing (WES), 81 regions from 27 tumors by whole genome sequencing (WGS), with six further tumors from the broader TRACERx Renal cohort also profiled by WGS (Figure S1B).

**Figure 1:**
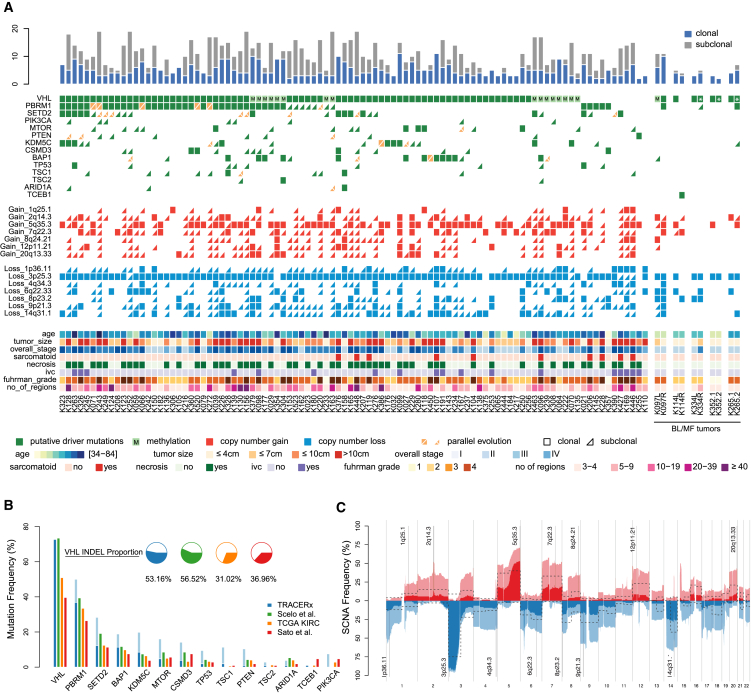
Overview (A) Overview of somatic driver alterations, including SNVs, DNVs, INDELs, and SCNAs, detected in the tumors of 101 TRACERx Renal cases. Rectangles and triangles indicate clonal and subclonal alterations, respectively. Parallel evolution is indicated in orange with a split indicating 2 or more parallel events. Five bilateral/multi-focal cases are shown on the right; distinct *VHL* mutations within tumor pairs are indicated with an asterisk. (B) Mutational frequency in 14 key driver genes in the TRACERx Renal cohort and three single biopsy ccRCC studies (TCGA KIRC, Sato et al. [2013], and Scelo et al. [2014]). Clonal mutations are shown in the darker shade, subclonal in lighter. (C) Frequency of SCNAs in the TRACERx Renal cohort. Copy number gains and losses are indicated in red and blue respectively. Clonal SCNAs are shown in darker and subclonal SCNAs in lighter shade of color. Putative driver copy number altered regions are annotated. The dotted line indicates the frequency of the same SCNAs in the TCGA KIRC cohort. See also Tables S1 and S2 and Data S1, S2, S3, and S4.

Median sequencing coverage across 1,206 tumor regions profiled by the Driver Panel was 612× (range, 105–1,520×). We identified a total of 740 somatic mutations including 538 SNVs (440 non-synonymous SNVs), 7 DNVs, and 195 INDELs (Table S2). We specifically considered non-silent mutations in high-confidence ccRCC driver genes (termed “driver mutations,” annotated in Figure 1A; STAR Methods). The median number of driver mutations was 3, range 0–15 per tumor (Figure 1A). *VHL* mutations were the only consistently clonal event, present in 77/106 tumors (Figure 1A). *VHL* was methylated in 17 additional tumors (Figure 1A and Data S1). One tumor harbored a clonal mutation in the *TCEB1* gene, a part of the *VHL* complex (Hakimi et al., 2015) (Figure 1A), thus 90% (95/106) of the tumors harbored clonal disruption of the *VHL* pathway. 4/11 *VHL* wild type tumors (K206, K228, K427, and K446) (Figure 1A) had evidence of sarcomatoid differentiation (Table S1), a feature reported to be associated with a lower frequency of *VHL* mutations (Malouf et al., 2016, Wang et al., 2017). K255, another *VHL* wild-type tumor, had evidence of both clear cell and papillary histology, and we observed SCNAs specific to both subtypes, including gains of 5q and 16 (Data S2). We observed no mutations in the known ccRCC driver genes in K110 (Figure 1A), and the copy number profile, involving whole chromosome losses on 1, 6, 10, and 17, was consistent with chromophobe RCC (Davis et al., 2014). Additional pathology review confirmed chromophobe histology, and K110 was removed from all subsequent analyses.

The overall frequency of driver mutations was higher in our cohort compared to the published single biopsy studies (Cancer Genome Atlas Research Network, 2013, Sato et al., 2013, Scelo et al., 2014) (Figure 1B). Notably, the frequency of *VHL* mutations in our and Scelo et al. (2014) studies was higher than that reported in the TCGA and Sato et al. (2013) studies, potentially due to the higher overall number of *VHL* INDELs called (Figure 1B). *VHL* INDELS in the TRACERx Renal cohort were all confirmed by Sanger sequencing (Data S1). The higher frequency of mutations in other driver genes was due to the detection of subclonal events through multi-region profiling in our cohort (Figure 1B).

An important goal of the TRACERx Renal study is to determine the contribution of SCNAs to clonal evolution. In ccRCC, recurrent SCNAs occur at a limited number of genomic sites (Beroukhim et al., 2009, Cancer Genome Atlas Research Network, 2013), usually as whole chromosome or chromosome arm events, and the rate of genome doubling is low (Zack et al., 2013). Therefore, recurrent SCNAs can be reliably detected by the Driver Panel, as shown by the high level of concordance with WGS results (Table S2). We measured the fraction of the tumor genome affected by SCNAs using the weighted genome instability index (wGII) (Endesfelder et al., 2014), taking the maximum observed wGII score across all regions per tumor. Maximum values were utilized in order to capture the potential highest risk, and hence most clinically relevant, subclones in each tumor (STAR Methods). Median wGII in the TRACERx Renal cohort was 32.8% (range, 4.7%–97.4%). All SCNAs were annotated using previously defined cytobands (Beroukhim et al., 2009) to quantify driver SCNAs (Figure 1A; STAR Methods). In total, we detected 751 driver SCNAs; median 7, range 1–14 per tumor (Figure 1A).

Loss of chromosome 3p, which is pathognomonic with ccRCC and encompasses four commonly mutated genes (*VHL*, *PBRM1*, *SETD2*, and *BAP1*), was observed in all but five tumors (K021, K375, K354, K255, K114R) (Figure 1A). Of the five, three tumors had clonal 3p copy neutral allelic imbalance (CNAI) (STAR Methods) (K021, K375, K354) (Data S3), one was a mixture of clear cell and papillary histology with no mutations in 3p genes (K255; Figure 1A), and one harbored a mutation in *TCEB1* with 8q loss (K114R; Figure 1A). 3p loss was subclonal in five tumors: one harboring a *VHL* mutation (K252) (Figure 1A), one *VHL* methylation (K070) (Figure 1A), one tumor that was *VHL* wild-type but *SETD2* muttant (K427) (Figure 1A), and two with no mutations in any of the 3p genes (K169, K446) (Figure 1A).

The overall frequency of driver SCNAs was higher compared to the published single biopsy studies (Cancer Genome Atlas Research Network, 2013, Sato et al., 2013, Scelo et al., 2014) due to the detection of subclonal SCNAs in our cohort (Figure 1C). Notably, the frequency of SCNAs with reported prognostic significance, such as loss of chromosomes 14q and 9p and gain of chromosomes 8q and 12p, is markedly underestimated in single biopsy studies (Cancer Genome Atlas Research Network, 2013). Overall ITH was measured as an index (ITH index = # subclonal drivers/# clonal drivers, where “drivers” include all driver mutations and driver SCNAs shown in Figure 1A) (STAR Methods). Median ITH index value was 1, with a high variability across the cohort (range, 0–13.5; SD = 2.16).

### Clonal Evolution and Clinical Variables in ccRCC

**Figure 2:**
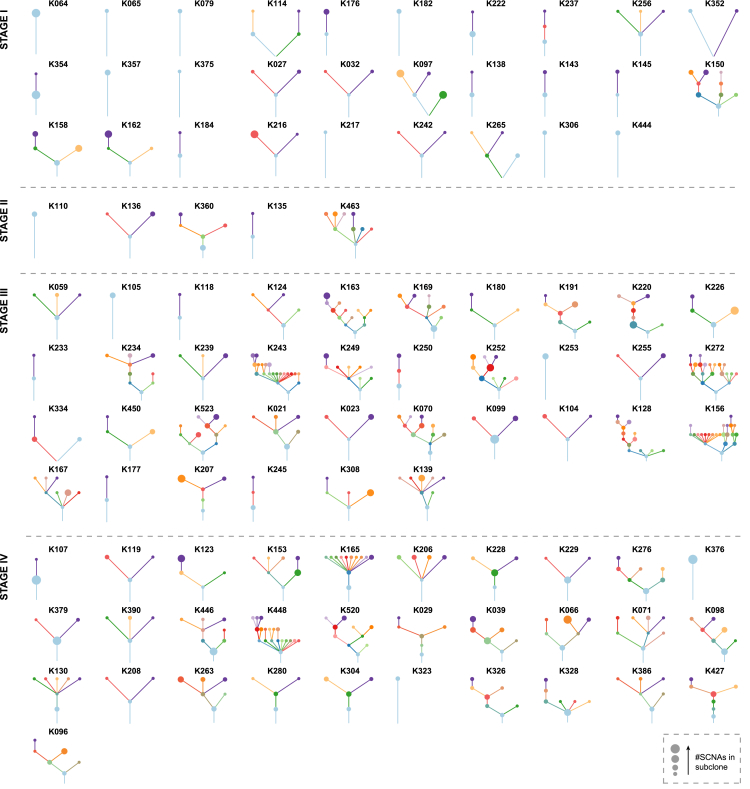
Driver Phylogenetic Trees Driver phylogenetic trees for each tumor (or multiple tumors from the same patient) are shown. The trees are ordered by the overall tumors stage: I–IV. The founding clone is indicated in light blue, with subsequent sub clones shown in distinct colors. The size of each node represents the number of SCNAs detected within that subclone. The length of lines connecting tumor subclones does not contain information. See also Data S2.

ccRCC prognostic variables include primary tumor size, overall tumor stage (TNM), Fuhrman grade, and the presence of necrosis. Overall, the number of driver events was significantly associated with all of these parameters, with the associations specific to subclonal, and not clonal events (Data S4). Similarly, higher ITH index values were associated with advanced tumor size, stage, and grade (Data S4). Clonal ordering techniques (see STAR Methods) were used to infer clonal structures and driver phylogenetic trees (Figure 2). The median number of clones detected was 4 per tumor (range, 1–23). Clone number increased with tumor stage and grade (Data S4), but showed a non-linear association with tumor size, initially increasing in line with tumor dimensions but then plateauing at ∼10 cm beyond which clone number began to marginally reduce with increasing size (Data S4). In conclusion, known prognostic parameters are associated with an increasing repertoire of driver alterations and subclonal driver diversification in ccRCC.

### Convergent Evolution

We profiled three patients with synchronous bilateral ccRCCs and two patients with multifocal ccRCCs, with no family history of ccRCC, or germline mutations in the known ccRCC predisposition genes (Table S1). All five tumor pairs evolved independently, but converged on the VHL pathway. K265, K352, and K334 harbored distinct mutations in *VHL* and 3p loss events in each of the tumors (Figure 1A and Data S3). The right-sided K097 tumor harbored a *VHL* mutation and *VHL* was methylated in the left tumor (Figures 1A and Data S1). Left K114 tumor harbored a *VHL* mutation and 3p loss, while in the right tumor we detected a clonal *TCEB1* mutation with the loss of 8q21.11, encompassing the *TCEB1* locus (Figure 1A). K150 tumor was presumed to be a contralateral renal metastasis from a previously resected left high-risk ccRCC. However, the two tumors had distinct *VHL* mutations (Data S1) implying a case of bilateral metachronous ccRCCs. Our findings illustrate the importance of molecular profiling of patients presenting with multiple renal tumors to guide appropriate clinical management.

### Parallel Evolution

**Figure 3:**
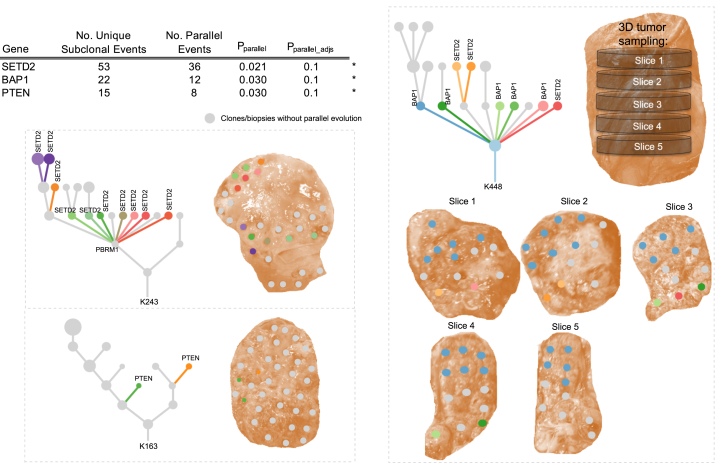
Parallel Evolution Table shows driver gene events with >10 subclonal mutations across the cohort. These genes were tested for evidence of parallel evolution using a permutation model accounting for overall gene mutation frequency and the number of biopsies per tumor (see STAR Methods). *BAP1*, *SETD2*, and *PTEN* were found to show significant evidence of parallel evolution (p < 0.05, FDR < 0.1). Example driver trees and accompanying tumor sampling images are presented for each significant gene: *BAP1*, *PTEN*, and *SETD2.* Parallel events are marked on the driver trees and clone color is matched from the tree to the corresponding sampled tumor region. See also Data S3.

We and others have reported parallel evolution of mutations in the same genes or pathways within distinct tumor subclones in ccRCCs (Brastianos et al., 2015, Gerlinger et al., 2014). In the TRACERx Renal cohort, 13% of untreated primary tumors had evidence of parallel evolution, with *SETD2*, *BAP1*, and *PTEN* (all p < 0.05, false discovery rate [FDR] <0.1) (Figure 3) significantly enriched for parallel evolution, corrected for the number of profiled regions. Certain tumors were notable for the number of parallel events they harbored (e.g., K243 had 10 distinct *SETD2* mutations) (Figure 3). In tumor K448, we observed 5 distinct *BAP1* mutations and 3 *SETD2* mutations, but *BAP1* and *SETD2* mutations never co-occurred within the same clone.

We recently identified parallel evolution of SCNAs in non-small cell lung cancer (Jamal-Hanjani et al., 2017) through mirrored subclonal allelic imbalance (MSAI) (Data S3). We analyzed the incidence of MSAI in a subset of TRACERx Renal patients where whole genome or exome sequencing data were available (n = 41) (STAR Methods) and observed MSAI events in 15/41 tumors (Data S3; STAR Methods), a subset of which were validated by an orthogonal method (Data S3). Parallel loss of chromosome 14q was the most common event (4 patients) (Data S3), encompassing the ccRCC tumor suppressor *HIF1A* locus (Shen et al., 2011).

### Identification of Conserved ccRCC Evolutionary Features

To understand the constraints of ccRCC evolution, we analyzed conserved patterns of driver event co-occurrence, mutual exclusivity and timing to identify statistically significant patterns. We utilized the clonal/phylogenetic hierarchy determined for each case (STAR Methods), in order to accurately place driver events within the same tumor subclone and establish the relative ordering of driver events across the evolutionary path of each tumor.

**Figure 4:**
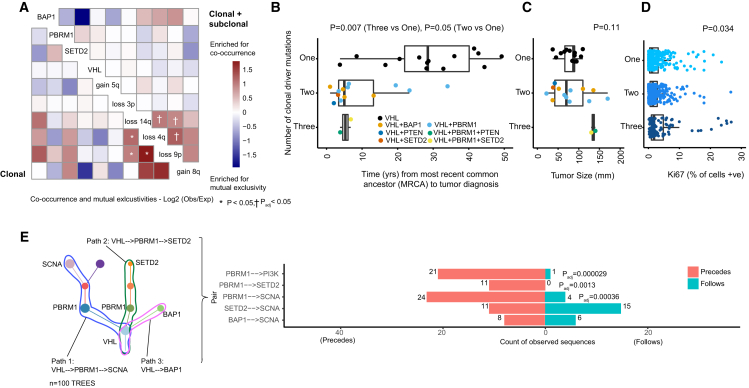
Conserved Features of ccRCC Evolution (A) Event co-occurrence analysis, with red indicating enrichment for co-occurrence and blue for mutual exclusivity. Values are log_2_(observed no. of co-occurrences/expected no. of co-occurrences, STAR Methods), with significant patterns marked according to the legend. Data are shown for event co-occurrence/mutually exclusivity, in first truncal clones only per case (bottom left) and second all terminal subclones (top right) such that all clonal and subclonal interactions are considered (see STAR Methods). p values are calculated under a probabilistic model, as implemented in R package “co-occur,” with only interactions significant in both “clonal” and “clonal + subclonal” analyses are considered significant. (B) Molecular clock timing analysis from the whole genome sequenced cohort, with time from the most recent common ancestor (MRCA) to tumor diagnosis plotted on the x axis. On the y axis are cases split into three groups, based on having one, two or three clonal driver events. *VHL* wild type cases (n = 2) are excluded on account of their distinct etiological and phenotypic profile. p value is assessed using a linear model, adjusting for the total clonal mutation burden per tumor. (C) Same y axis patient groups as (B), but plotted on the x axis is tumor size (mm). p value is based on Kruskal-Wallis test. (D) On the y axis, all cases from the 100-patient cohort, again *VHL* wild-type cases were then excluded, and remaining cases were split into three groups based on one, two, or three clonal driver mutations. Multi-region data on % of cells staining positive for proliferation marker Ki67 is shown on the x axis. p value is based on a linear mixed effect model to account for non-independence of multiple observations per tumor. (E) Left: an illustrative schematic tree to demonstrate the method used to trace each tumor’s evolutionary paths. Right: results from the event ordering analysis for all pairs of events with n = 10 or more observations. Plotted are the counts of instances where: event 1 was found to precede event 2, and event 1 was found to follow event 2. Significance was tested using a binomial test with p values shown after correction for multiple testing using Benjamini-Hochberg procedure. See also Figure S2 and Table S3.

**Figure S2:**
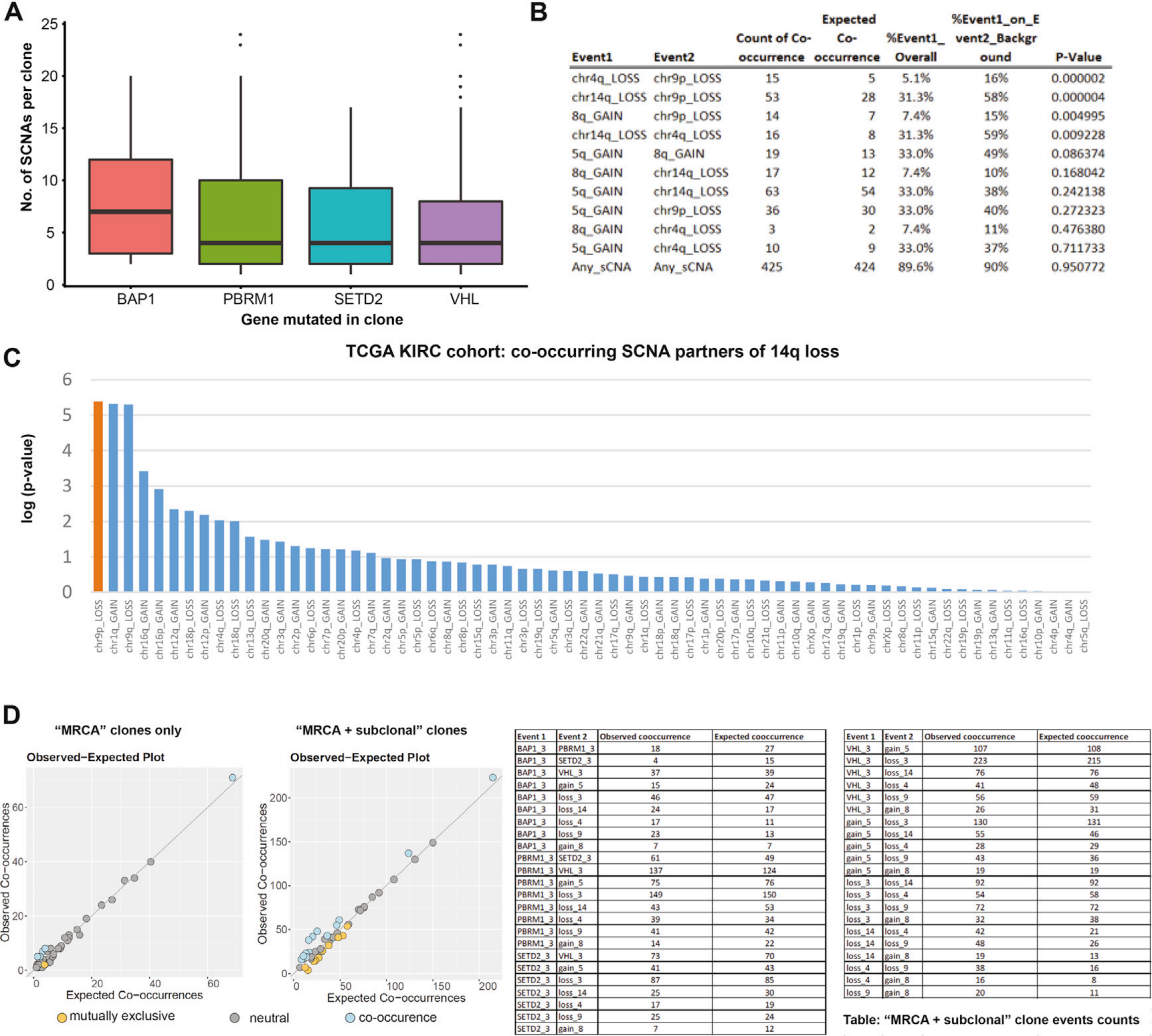
SCNAs Co-occurring with Mutational Driver Events, Related to Figure 4 (A–D) (A) shows SCNAs co-occurring with mutational driver events in TRACERx Renal cohort. (B) shows SCNA co-occurrence in TCGA KIRC cohort. (C) shows 14q loss co-occurring with the other SCNAs. 14q loss is shown on X-axis and on Y-axis is log(p-value) for co-occurrence. (D) shows observed versus expected co-occurrence frequencies.

In our analyses of event co-occurrences at the clone level (STAR Methods), we observe an enrichment for mutual exclusivity between *BAP1* and *SETD2*/*PBRM1* mutations (Figure 4A)*.* However, at a patient level these events were found to co-occur (Figure 1A), often in separate spatially distinct major tumor subclones (e.g., K153) (Data S2). *BAP1* had a propensity for being a lone additional mutational driver event in *VHL*-mutant clones, whereas *PBRM1* and *SETD2* were enriched for mutual clonal co-occurrence. Due to limited sample size, these patterns did not reach formal significance, however, we note the results are in agreement with previously published patient-level meta-analysis (Peña-Llopis et al., 2013). Of all the driver mutations, *BAP1* was associated with the highest number of driver SCNAs in the same clone (Figures 4A and S2, p = 0.014 for *BAP1* mutant clones versus *BAP1* wild-type clones), consistent with its role in chromosome stability (Peng et al., 2015). Overall, the strongest evidence for co-occurrence was found for the following pairs of driver SCNAs: 14q loss with 4q loss, 14q loss with 9p loss, and 4q loss with 9p loss (Figure 4A, all p < 0.05, adjusted for multiple testing). These pairs of events were all found to co-occur ≥1.8 times more frequently than expected by chance. We validated these observations in the TCGA ccRCC data (all p < 0.05, Figure S2), showing that the specific event pairings co-occurred together beyond the general expected correlation between SCNAs (e.g., for 14q loss, the most common partner event genome wide was 9p loss, Figure S2). We note that these SCNAs harbor well-known tumor suppressors 14q31.1-*HIF1A* (Shen et al., 2011), 9p21.3-*CDKN2A* (Beroukhim et al., 2009), and 4q-*CXXC4* (Kojima et al., 2009).

In our previous report of ten ccRCC tumors (Gerlinger et al., 2014), mutations in *VHL* and loss of 3p were consistently clonal, and *PBRM1* was an additional clonal driver mutation in three cases. In our current prospective cohort, we observed a subset of cases that harbored two or more additional clonal driver mutations, aside from *VHL*. Simulated models of tumor growth (Reiter et al., 2013) suggest that just one additional driver will significantly increase the growth rate, and we utilized WGS molecular clock timing data (see the accompanying paper by Mitchell et al., 2018) to test this hypothesis in our data. Time to presentation was calculated as the time elapsed from the emergence of the most recent common ancestor (MRCA) to clinical diagnosis. The median time to presentation from the emergence of the MRCA for cases with *VHL* as the only clonal driver mutation, (n = 14 cases, 48% of the WGS cohort) was 28 years (min = 4, max = 49). The addition of one further clonal driver mutation (n = 13 cases) was associated with a shortening of time to diagnosis, to 5 years (min = 1, max = 34), and the addition of two further clonal driver mutations (n = 2 cases) shortened the time to diagnosis to 5 years with a narrow range (min = 4, max = 7) (p = 0.007, Figure 4B). Despite the shortened time of tumor growth, tumor size was found to be comparable across all the groups (Figure 4C), and we observed no difference in the mode of presentation (incidental versus symptomatic) across the three groups, suggesting there was no lead-time bias. Overall, the groups had the same total median number (n = 3) of driver mutations (considering clonal and subclonal events). Assessment of proliferation by multiregional Ki67 immunohistochemistry (IHC) staining (STAR Methods) showed elevated proliferation index in cases with additional clonal driver mutations (p = 0.034, Figure 4D; Table S3), consistent with the simulation (Reiter et al., 2013).

### Order of Events during ccRCC Evolution

The order in which driver events are acquired can have prognostic and therapeutic implications, as shown by Ortmann et al. (2015) with respect to the order of *JAK2* and *TET2* mutations in myeloproliferative neoplasms. We considered the ordering of driver events in ccRCC, assessing for recurrent patterns of driver events preceding or following one another. To conduct this analysis, we traced all possible evolutionary trajectories, starting at the base of each driver tree and tracing the path through to each terminal subclone, considering all possible sequential paths between events (Figure 4E). Due to the dense spatial sampling in this cohort the driver tree ordering was typically robust, with evidence of sequential waves of clonal expansion between events usually confirmed across multiple biopsy regions. In order to reduce the burden of multiple testing, we limited further analyses to those trajectories containing the most frequent ccRCC driver events: *VHL*, *PBRM1*, *SETD2*, *BAP1*, PI3K/AKT/mTOR pathway mutations, or driver SCNAs (Figure 1B). Event combinations that we observed in ten or more cases were then tested for significance in the ordering pattern (STAR Methods). Six significantly conserved patterns were detected (all FDR <0.05), the first three of which confirmed *VHL* as a universally preceding event, as expected. In addition, *PBRM1* mutations were found to consistently precede PI3K pathway mutations, *SETD2* mutations, and driver SCNA events (Figure 4E). In many of these cases, the event sequences were observed exclusively in one direction (i.e., *PBRM1* precedes *SETD2* in 11 separate cases), but the opposite was never observed.

### Evolutionary Subtypes

**Figure 5:**
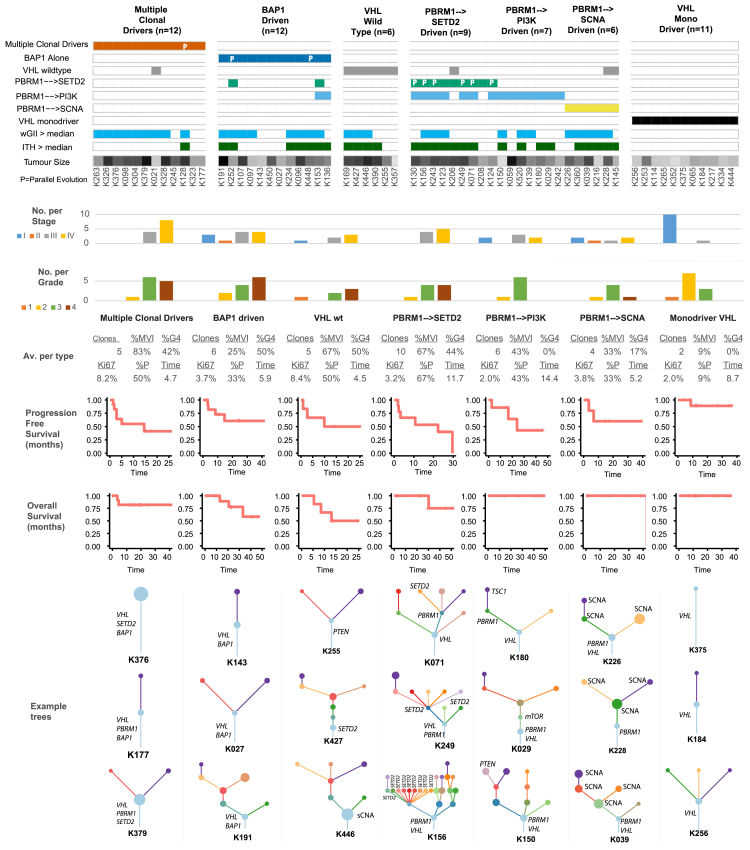
Evolutionary Subtypes Cases grouped by evolutionary subtype, with the following parameters also annotated: presence of clonal wGII (blue > median, white ≤ median), presence of subclonal wGII (blue > median, white ≤ median), ITH index score (red > median, white ≤ median), and tumor size (mm) (range [18–180], white = low, black = high). Occurrences of parallel evolution are denoted in the heatmap with “P.” Plotted next is the distribution of stages per subtype, followed by grade, colored as per the legend, and then a further six metrics are summarized as the average values for each group: (1) mean number of tumor clones, (2) % of patients with grade 4 disease, (3) % of patients with microvascular invasion, (4) mean % of cells staining positive for Ki67 proliferation index (mean calculated first per class and then across the cohort), (5) % of patients with disease relapse/progression, and (6) relapse/progression time. Shown next are relapse/progression-free survival plots per group, and shown last are three example driver phylogenetic trees from each group. See also Figure S3.

**Figure S3:**
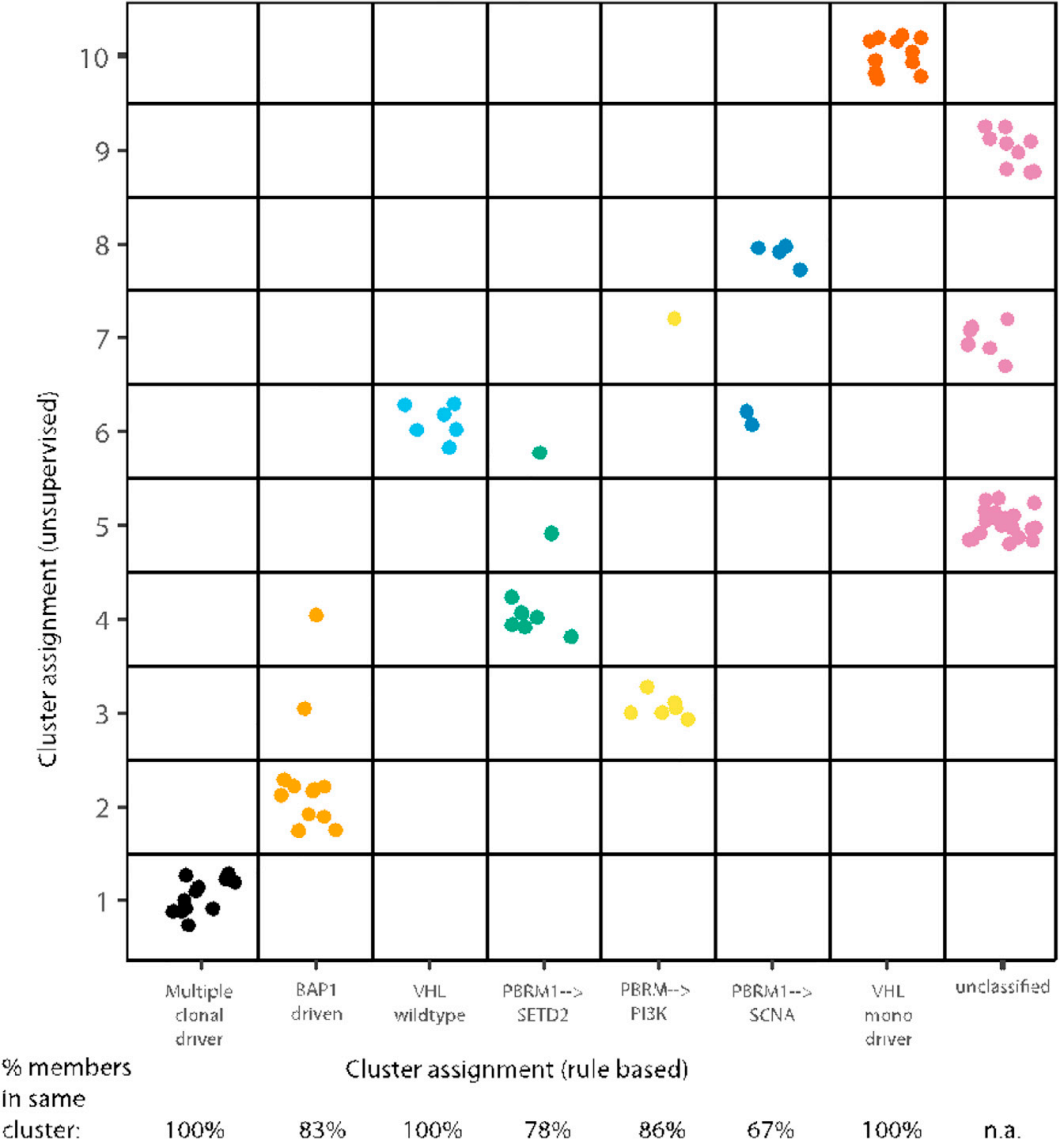
TRACERx Renal Cohort Unsupervised Clustering Analysis of Evolutionary Features, Related to Figure 5 and STAR Methods On the x-axis are the rule based evolutionary subtype groups, and on the y-axis are group assignments based on unsupervised clustering. Shown below the x-axis is the percentage of members, from each evolutionary subtype, which are assigned to the same unsupervised cluster. Colours have no meaning except to denote different groups.

A pertinent question is whether conserved patterns of ccRCC evolution relate to distinct clinical or biological phenotypes; to investigate this in an exploratory context we classified all the tumors under study according to the patterns observed in the evolutionary order, timing, and co-occurrence analyses (Figure 4). Seven evolutionary subtypes were defined (Figure 5) using a rule-based classification system (STAR Methods), which was supported by unsupervised clustering (Figure S3). Subtypes were compared across different genomic and clinical metrics (STAR Methods) including levels of wGII, percentage of cells positive for Ki67, ITH index, clonal structure, and clinical parameters including stage, percentage of tumors that are Fuhrman grade 4 (%G4), or presence of microvascular invasion (%MVI) (Figure 5). The first subtype consisted of tumors with “multiple clonal drivers” (defined as ≥2 *BAP1*, *PBRM1*, *SETD2*, or *PTEN* clonal mutations), and was characterized by high levels of wGII (9 out of 12 cases with wGII > cohort wide median value), enrichment for late stage disease (all cases were stage III+) and a high level of %MVI/%G4/%Ki67. These tumors harbored a smaller number of clones (mean = 5, range [1–14]) and had limited ITH (11 out of 12 cases had ITH < cohort wide median value) (Figure 5; STAR Methods). This pattern would be consistent with sufficient selective fitness being achieved within the dominant clone through fixation of multiple driver mutations and SCNAs causing a clonal sweep during tumorigenesis.

A second and related subtype comprised “*BAP1* driven” cases characterized by tumor clones with *BAP1* as a lone mutational driver in addition to *VHL* (Figure 5). Where the tumors harbored other driver mutations, they were never found in the same subclone as the *BAP1* mutation (K448, K252, K153, K136) (Figure 1 and Data S2). This group was enriched for tumors with elevated wGII (8 out of 12 > median), fewer clones, and a higher tumor grade (%G4). This pattern suggests that *BAP1* mutations coupled with SCNAs afford a fitness advantage such that no additional driver events become fixed making them terminal drivers within individual clones. The third subtype consisted of “*VHL* wild-type” tumors, characterized by high Ki67% (highest across all groups), elevated levels of wGII, potentially compensating for a lack of driver mutations, and additional phenotypic differences such as frequent presence of sarcomatoid differentiation.

The fourth subtype was “*PBRM1* → *SETD2*”-driven, a group characterized by highly branched trees (>10 clones per tumor; range [3–23]), the highest mean ITH score in the whole cohort, lower Ki67%, frequent parallel evolution events, and advanced disease stage (Figure 5). This pattern would be consistent with the notion of slower branched growth with early *PBRM1* mutations followed by strong and repeated selection for *SETD2* mutations. Supporting this notion was the mean time-to-progression (defined as time-to-progression following cytoreductive nephrectomy or the time-to-relapse following nephrectomy with curative intent) in this group (11.7 months), which was more than twice as long as that for “multiple clonal driver,” “*BAP1* driven,” and “*VHL* wild-type” tumors (4.7, 5.9, and 4.5 months, respectively, not formally significant). Critically, the observed features of this subtype were independent of tumor size, with no significant difference between the highly branched “*PBRM1* → *SETD2*” (mean tumor size, 105 mm) (Table S1) and the more monoclonal “multiple clonal driver” subtype (mean tumor size, 107 mm) (Table S1). The fifth and sixth subtypes were “*PBRM1* → PI3K” and “*PBRM1*→ SCNA,” characterized by early *PBRM1* mutation followed by mutational activation of the PI3K/AKT/mTOR pathway or subclonal SCNAs, respectively, and enriched for lower grade tumors.

The final evolutionary subtype consisted of the “*VHL* mono-driver” tumors, which displayed limited branching and a monoclonal structure, with no additional driver mutations and low wGII. The majority of tumors in this group presented at an early stage (mean tumor size, 45 mm) suggesting they may be an early evolutionary ancestor of the more complex subtypes described above. Small renal masses (SRMs) without evidence of vascular or fat invasion (T1a) are an increasingly common clinical entity, which can potentially be managed by active surveillance (Jewett et al., 2011). We note that the only ≤4 cm tumor that was upstaged due to the presence of renal vein invasion (K021) was in the “multiple clonal driver” category, consistent with this evolutionary path enhancing vascular invasion independent of tumor size.

Specific evolutionary subtypes could not be assigned in 37 cases from a wide distribution of disease stages (stage I = 12, II = 2, III = 16, IV = 7). These tumors are likely to be driven by rarer evolutionary patterns not yet identifiable with current sample sizes. Several appeared to exhibit precursor subtype features (e.g., clonal *VHL* mutation) followed by *PBRM1* mutation in a major subclone, that may have continued to evolve if they remained *in situ*. Further elucidation of the genomic and non-genomic drivers of evolutionary subtypes in larger datasets will be of major interest.

### ITH Index and Saturation of ccRCC Driver Events

**Figure 6:**
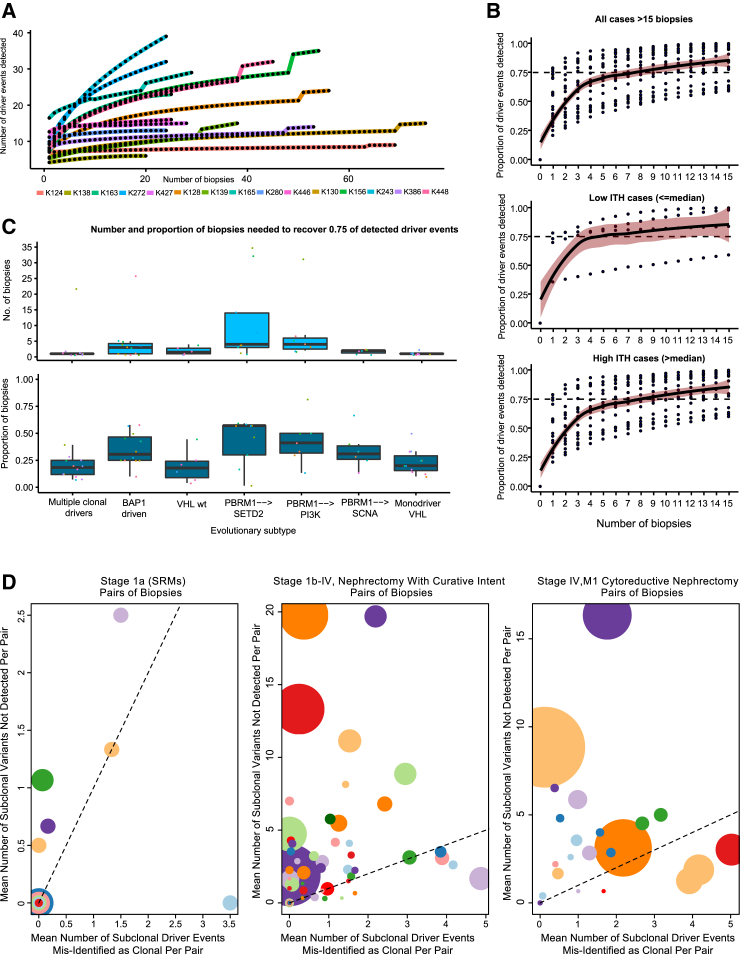
Intratumor Heterogeneity Index and Saturation Analysis (A) Number of tumor biopsies profiled (x axis) versus the number of driver events (i.e. all gene mutations and SCNAs shown in Figure 1A) discovered (y axis) for densely sampled (20+ biopsies) cases. (B) Saturation curves for all cases with ≥15 biopsies, with biopsy number plotted on x axis and proportion of the total driver events detected (from all biopsies) on y axis, increasing with each additional biopsy taken. Data are shown for all cases and tumors split based on low and high ITH (above/below median). (C) Boxplot summary of the absolute number (top) of biopsies needed to detect ≥0.75 of driver events for tumors grouped by evolutionary subtype. Also shown (bottom) is the proportion of biopsies needed (out of the total number taken from each tumor) to normalize for absolute biopsy count. (D) Illustration of the potential errors arising from a two-site biopsy approach: considering all pairs of biopsies, plotted on the x axis is the mean number of subclonal driver events misidentified as clonal (illusion of clonality), on y axis is the number of subclonal driver events missed entirely. Data are shown for three clinical scenarios. Left: small renal masses (size, <4 cm). Middle: tumors treated by nephrectomy with curative intent. Right: tumors treated by cytoreductive nephrectomy. The size of points within a panel is proportional to the number of biopsies available for that tumor and colors vary only to distinguish overlapping points.

While pervasive ITH has been described in multiple tumor types, only one prospective study of multiregional tumor profiling has been reported to date (Jamal-Hanjani et al., 2017). TRACERx Renal, with 1,206 primary tumor biopsies profiled across 101 ccRCC cases, affords an unprecedented opportunity to systematically explore the ITH extent. In a subset of tumors (n = 15) that underwent extensive sampling (≥20 biopsies), we considered driver event (mutation and SCNA) “saturation,” measured as the proportion of events discovered with each additional tumor region profiled. Our analysis revealed a wide spectrum of saturation gradients (Figure 6A), highlighting the challenge of attempting to establish a biopsy count reliably applicable to all ccRCCs. Accepting this caveat, and considering all the tumors with ≥15 biopsies (n = 20) we calculated the stepwise change in driver event discovery when using between 1 to 15 biopsies (Figure 6B). On average, two biopsies were required to detect ≥50% of all variants and seven were required to detect ≥75% of all variants (Figure 6B). As expected, these values changed markedly based on tumor ITH, with homogenous tumors (≤median ITH index) achieving ≥0.75 detection within four biopsies, as opposed to eight biopsies required for heterogeneous tumors (>median ITH) (Figure 6B). Splitting instead by evolutionary subtype, fewest biopsies were needed to reach 0.75 driver detection in the “multiple clonal driver” and “*VHL* monodriver” groups, and largest number for “*PBRM1* → *SETD2*” tumors (Figure 6C).

We considered the utility of a radiologically guided two-site biopsy approach for primary tumors that present as an SRM, or larger tumors without (M0) or with metastases (M1). We down-sampled our dataset to two biopsies per tumor (STAR Methods) and considered the mean results across all possible combinations to simulate how many subclonal driver events would be missed and how many subclonal events would be misclassified as clonal (“illusion of clonality”). For the SRM group, 11/15 tumors had a mean of ≤1 driver event missed and ≤1 driver event misclassified as clonal with a paired biopsy approach (Figure 6D, panel 1). For larger tumors, whether metastatic or not, performance was less favorable, with the majority suffering from multiple missed subclonal drivers and/or events misclassified as clonal (Figure 6D, panels 2 and 3). For these tumors, our data suggest that a range of four to eight biopsies is required to capture the majority of events (≥75% detection), although this approach may still miss some important drivers.

### Clonal Evolution and Clinical Significance

**Figure 7:**
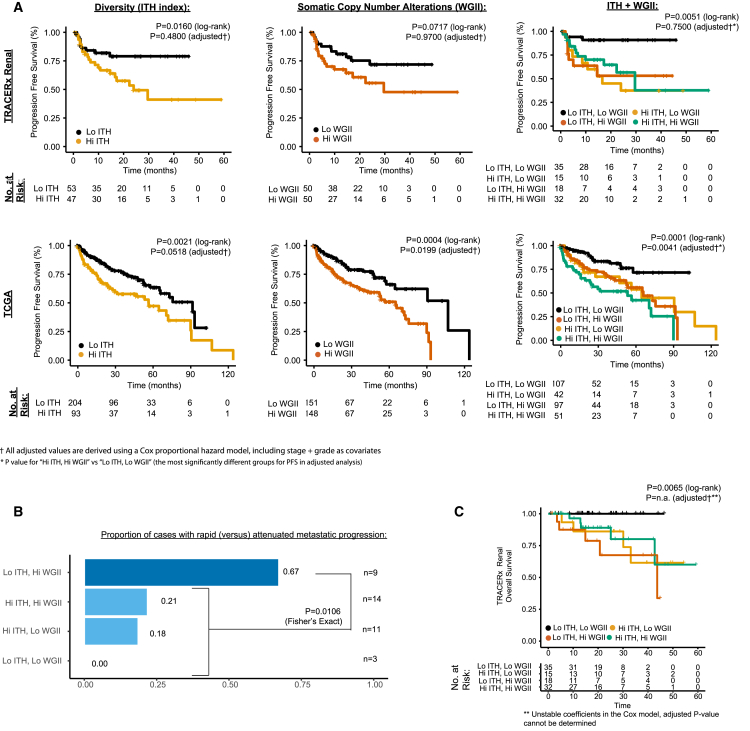
Clinical Endpoints (A) Kaplan-Meier plots for progression free survival (PFS) in the TRACERx Renal cohort (three plots in top row) and for overall survival (OS) in TCGA KIRC cohort (three plots in bottom row). Three groupings are plotted for each cohort. Left: high (>median) versus low ITH index. Middle: high (>median) versus low wGII. Right: four group high/low combination groupings of the two metrics. Log-rank and adjusted (for stage and grade as covariates in a Cox proportional hazard model) p values are stated. (B) Proportion of cases, within each of the high/low four groups, that progressed to disseminated versus solitary metastases, based on each patient’s first progression event. Counts in the highest group “low ITH, high wGII,” were compared to all other groups through Fisher’s exact test. (C) Cancer-related deaths OS analysis (as opposed to PFS shown in A) for the TRACERx Renal cohort, with patients grouped using the four-category high/low ITH/wGII system. Log-rank and adjusted (for stage and grade as covariates in a Cox proportional hazard model) p values are stated. See also Table S4.

Association of the ITH index and disease progression was a pre-defined endpoint of the TRACERx Renal study (Turajlic and Swanton, 2017). We therefore assessed whether patients whose tumors had high ITH index (>median value) had significantly reduced progression free survival (PFS), compared to those with low ITH index. While we detected this in a univariate analysis (p = 0.0160 log-rank, hazard ratio [HR] [95% confidence interval (CI)] HR = 2.4 [1.1–5.2]), the association was not significant when adjusted for known prognostic variables in a Cox proportional hazards model (p = 0.4800 adjusted) (Figure 7A; STAR Methods). As elevated wGII was consistently enriched in the high risk evolutionary subtypes, we also considered its association with PFS. Patients in our cohort whose tumors had high wGII (>median value) had a non-significant trend towards shorter PFS compared to those with low wGII (p = 0.0717 log-rank HR = 1.9 [0.9–4.0], p = 0.9400 adjusted) (Figure 7A). To determine whether the absence of significance may simply be a function of the sample size of this intermediate cohort, we further investigated ITH and wGII metrics in the larger and more robustly powered TCGA KIRC cohort and found both measures to be significantly associated with PFS (p = 0.0021 HR = 1.9 [1.2–2.8] and p = 0.0004 HR = 2.1 [1.4–3.3], respectively, log-rank). Importantly, this association remained independently significant after adjusting for stage and grade (p = 0.05 HR = 1.5 [1.0–2.3] and p = 0.02 HR = 1.7 [1.1–2.6], respectively, adjusted) (Figure 7A), and in addition, both measures were found to be significantly associated with overall survival (OS) in an adjusted analysis (p = 0.04 HR = 1.7 [1.0–2.7] and p = 0.04 HR = 1.7 [1.0–2.8], respectively, adjusted) (Table S4). We note that the single biopsy approach is likely to have reduced the sensitivity to detect ITH and subclonal SCNAs in the TCGA cohort.

Next, we considered ITH and wGII measures in combination, to ascertain if a low score in one measure but high in the other was sufficient on its own to be associated with increased patient risk. Significantly reduced survival was observed in all groups compared to “Low ITH and Low wGII”, suggesting that either driver event intratumor heterogeneity, or a homogeneous profile with high wGII (e.g., “Multiple Clonal Driver” evolutionary subtype), were the underlying factors associated with poor prognosis (TRACERx Renal 100: p = 0.0019 log-rank, p = 0.7500 adjusted, TCGA PFS: p = 0.0025 log-rank, p = 0.0041 adjusted, Figure 7A, TCGA OS: p = 0.0001 log-rank, p = 0.0040 adjusted, see Table S4 for full TCGA Cox model results).

We finally considered whether ITH and wGII measures associated with the pattern of metastatic progression. Within our cohort, 37 patients developed progressive disease, and we classified their disease progression (following cytoreductive or curative intent nephrectomy) into “rapid” or attenuated” (Table S1; STAR Methods). 67% (n = 9) of “Low ITH, High wGII” patients had rapid progression, as compared to 18% (n = 28) in the other three groups (p = 0.0106, Fisher’s exact) (Figure 7B). Although limited by a small number of events (n = 14), overall cancer-specific survival analysis (as opposed to PFS) in our cohort also demonstrated an association between ITH/wGII metrics and patient survival (p = 0.0065 log-rank). The shortest survival time was observed in the “Low ITH, High wGII” group, further highlighting the aggressive nature of homogeneous tumors with high wGII, a measure reflecting early fixation of chromosomal complexity (Figure 7C; Turajlic et al., 2018).

## Discussion

We used clonal event co-occurrence, mutual exclusivity, and temporal ordering to reveal deterministic features of ccRCC evolution and infer seven evolutionary subtypes. The “multiple clonal drivers” subtype was characterized by clonal co-occurrence of drivers that are usually mutually exclusive (*BAP1* and *PBRM1*; *BAP1* and *SETD2*), pointing to their combination being both tolerated and advantageous in certain contexts. These tumors had high wGII and low diversity, suggesting high clonal fitness with limited ongoing selection or a clonal sweep. Despite being the largest tumors in the whole cohort, they had the shortest time from the most recent common ancestor to diagnosis, consistent with a clonal sweep and accelerated tumor growth, due presumably to the presence of additional drivers as shown in simulated models of tumor growth (Reiter et al., 2013). We note that our findings are in keeping with the observation of an aggressive subgroup of ccRCC with the concurrent loss of expression of *PBRM1* and *BAP1*, a likely surrogate for the “multiple clonal drivers” subtype (Joseph et al., 2016). The “*BAP1* driven” subtype confirmed the tendency to mutual exclusivity between *BAP1* and *PBRM1* (Peña-Llopis et al., 2013) mutations at the clone level. The majority of these tumors had no other detectable mutational drivers, suggesting that *BAP1* mutations combined with SCNAs drive a robust clonal expansion. Accordingly, in a recently published mouse model of ccRCC, co-targeting of *VHL* and *BAP1* resulted in high grade tumors with short latency (Gu et al., 2017).

At the other end of the evolutionary spectrum the “*PBRM1* → *SETD2*” tumors had extensive branching, high ITH, and preponderance for parallel evolution. The conserved ordering of *SETD2* and *PBRM1* mutations and the strong repeated selection of *SETD2* mutant subclones that induce a limited clonal expansion raise interesting biological questions. It is possible that this sequence of events cannot achieve broader clonal growth due to a narrow selective fitness or because it occurs after the primary tumor bulk is established. The spatial clustering of parallel *SETD2* mutations suggests a potential role for niche-specific selection, or even niche construction by the *SETD2* mutant subclones.

*PBRM1* mutations are highly enriched as an early event in ccRCC, evidenced by their being clonal in 74% of cases, but also by the “*PBRM1* → PI3K” and “*PBRM1* → SCNA” evolutionary subtypes. In a mouse model of ccRCC (Gu et al., 2017), co-targeting of *VHL* and *PBRM1* led to low grade ccRCC tumors that arose late, while an aggressive phenotype was triggered by the additional disruption of *TSC1*, a component of the PI3K pathway. Thus, although *PBRM1* is frequently selected early on, it appears to have a strong necessity for later subsequent driver events.

The “*VHL* wildtype” tumors were characterized by high wGII of cryptic etiology and were enriched for sarcomatoid differentiation, while the “*VHL* monodriver” tumors had few driver events and low wGII, and were enriched for SRMs.

The evolutionary subtype group sizes were too small for formal survival analysis, and assessment in the full TRACERx Renal study cohort (target n = 320) will be of significant interest. Nevertheless, the combination of the features critical in distinguishing the evolutionary subtypes, diversity (ITH), and chromosomal complexity (wGII) was prognostic in our and the TCGA KIRC cohort. Low diversity, high wGII tumors were more likely to progress rapidly and widely, suggesting the presence of occult metastases at presentation, while heterogeneous tumors (high ITH) with or without high wGII, were more likely to have an attenuated progression pattern, often with solitary metastasis. Thus, cytoreductive nephrectomy, metastasectomy, or deferral of systemic therapy may not be beneficial in the low diversity/high wGII cases, and ongoing investigations will determine if the proposed classification could help to optimize the benefit from these interventions.

An increasingly important area of clinical management are SRMs, which account for almost one-half of all newly diagnosed renal masses (Kane et al., 2008). There is an ongoing debate about their treatment due the low rate of progression observed during active surveillance (Jewett et al., 2011). The majority of SRMs in our cohort had low ITH and low wGII, consistent with high cure rates achieved with early surgical intervention. These tumors could potentially be amenable to observation. However, some SRMs in our cohort were characterized by high ITH or wGII and could progress in the absence of surgical intervention. Therefore, evolutionary classification could aid an active surveillance strategy in the context of SRMs.

The number of driver events required for tumor initiation, maintenance, and progression is subject of active debate and study (Tomasetti et al., 2015). We observed an extensive repertoire of disease drivers, with up to 30 mutational and SCNA driver events detectable in a single tumor. The question remains how many biopsies are required to determine the panoply of disease drivers. While it appears that the gain in driver detection per additional biopsy begins to decline after ∼8 biopsies, in some tumors, especially the “*PBRM1* → *SETD2*” subtype, a large number of driver events would still be missed if only ∼8 biopsies are taken. Without taking into account the spatial arrangement of the tumor biopsies, we note a two-site biopsy approach recovers nearly all subclonal driver events in the majority of SRMs with a moderate risk of illusion of clonality. For larger tumors, our data suggest a biopsy number in the range of four to eight is required to capture the majority of events. We recognize that in the setting of clinical practice, molecular profiling of multiple biopsies will not be practical, and alternative approaches are needed.

Our data account for a number of clinical and experimental observations in ccRCC and highlight important evolutionary principles. Clonal co-occurrence of multiple drivers resulting in a clonal sweep is consistent with the hypothesis of punctuated evolution, proposed as an alternative to phyletic gradualism by Eldredge and Gould (1997), while the contribution of chromosomal complexity to an aggressive phenotype has parallels with Goldschmidt’s view of macroevolution, in *Material Basis of Evolution* (Goldschmidt, 1940). We acknowledge, however, that both micro and macro evolution, as well as non-genetic diversity, are likely to influence clinical outcomes. Finally, while evolutionary contingency was clearly evident in patients with multiple independent primary tumors, the deterministic nature of ccRCC evolution was illustrated by the highly conserved sequence of driver events. We conclude that an understanding of the clonal dynamics and the evolutionary potential of a tumor provide biological insight as well as a potential rationale for clinical decision-making.

## STAR★Methods

### Key Resources Table

**Table:** 

REAGENT or RESOURCE	SOURCE	IDENTIFIER
**Deposited Data**		

Multi-region sequencing data on TRACERx renal 101 patient cohort.	This study.	EGAS00001002793

**Oligonucleotides**		

Oligonucleotide sequences for VHL exon amplification and methylation specific PCR see Data S1.	This paper	N/A

**Software and Algorithms**		

Burrows-Wheeler Aligner (BWA) v0.7.15	Li and Durbin, 2009	http://bio-bwa.sourceforge.net/
Samtools v1.3.1	Li and Durbin, 2009	http://samtools.sourceforge.net/
Picard 1.81	N/A	http://broadinstitute.github.io/picard/
Mutect v1.1.7	Cibulskis et al., 2013	http://archive.broadinstitute.org/cancer/cga/mutect
VarScan v2.4.1	Koboldt et al., 2009	http://varscan.sourceforge.net/
Scalpel v0.5.3	Fang et al., 2016	https://github.com/hanfang/scalpel-protocol
Annovar	Wang et al., 2010	http://annovar.openbioinformatics.org/en/latest/
CNVkit v0.7.3	Talevich et al., 2016	https://github.com/etal/cnvkit
R package ‘Copynumber’	Nilsen et al., 2012	http://bioconductor.org/packages/release/bioc/html/copynumber.html
ABSOLUTE v1.0.6	Carter et al., 2012	http://archive.broadinstitute.org/cancer/cga/absolute
bedtools package	Quinlan and Hall, 2010	http://bedtools.readthedocs.io/en/latest/
R package ‘TRONCO’	De Sano et al., 2016	http://www.bioconductor.org/packages/release/bioc/html/TRONCO.html
PyClone	Roth et al., 2014	https://bitbucket.org/aroth85/pyclone/wiki/Home
AlleleCounter	N/A	https://github.com/cancerit/alleleCount
ASCAT	Van Loo et al., 2010	https://github.com/Crick-CancerGenomics/ascat
Battenberg	Nik-Zainal et al., 2012	https://github.com/cancerit/cgpBattenberg
R package ‘cooccur’	Griffith et al., 2016	https://cran.r-project.org/web/packages/cooccur/index.html
R package ‘Trajectory Miner’	Gabadinho et al., 2011	http://traminer.unige.ch/

### Contact for Reagent and Resource Sharing

Further information and requests for resources and reagents should be directed to and will be fulfilled by the Lead Contact, Charles Swanton (Charles.swanton@crick.ac.uk).

### Experimental Model and Subject Details

Patients were recruited into TRACERx Renal, an ethically approved prospective cohort study (National Health Service Research Ethics Committee approval 11/LO/1996). The study sponsor is the Royal Marsden NHS Foundation Trust. The study is coordinated by the Renal Unit at the Royal Marsden Hospital NHS Foundation Trust. The study is open to recruitment at the following sites: Royal Marsden Hospital NHS Foundation Trust, Guy’s and St Thomas’ Hospital NHS Foundation Trust, Royal Free Hospital NHS Foundation Trust and Western General Hospital (NHS Lothian). Patients were recruited into the study according to the following eligibility criteria:

#### Inclusion criteria

• Age 18- years or older
• Patients with histologically confirmed renal cell carcinoma, or suspected renal cell carcinoma, proceeding to nephrectomy/metastectomy
• Medical and/or surgical management in accordance with national and/or local guidelines
• Written informed consent (permitting fresh tissue sampling and blood collection; access to archived diagnostic material and anonymised clinical data)

#### Exclusion criteria

• Any concomitant medical or psychiatric problems which, in the opinion of the investigator, would prevent completion of treatment or follow-up
• Lack of adequate tissue

Further eligibility criteria were applied to the cohort presented in this paper (it therefore follows that these patients do not have consecutive study ID numbers from 001 to 100):

• Confirmed histological diagnosis of clear cell renal cell carcinoma.
• No documented germline renal cell carcinoma predisposition syndrome (including *VHL*).
• At least three primary tumour regions available for analysis.

The cohort was representative of patients eligible for curative or cytoreductive nephrectomy. Full clinical characteristics are provided in Table S1. Demographic data include: Sex, Age and Ethnicity. Clinical data include: Presenting symptoms, Smoking status, BMI, History of Previous RCC, Family History of RCC, Bilateral or Multi-focal RCC, Neoadjuvant therapy (6 patients received systemic therapy prior to nephrectomy). Histology data include: overall TNM Stage (based on Version 7 classification), Location of nephrectomy, Number of harvested and involved lymph nodes, presence of Microvascular Invasion, presence of Renal Vein Invasion, presence of IVC tumour thrombus, Size of primary tumour, Leibovich score, Fuhrman Grade, Time to nephrectomy (days). Clinical status of patients included: Relapse free survival (months), Total follow up (months), Survival Outcome. 16 patients were lost to follow-up: 8 were stage I, 5 stage III and 3 stage IV. For clinical parameter correlation and outcome analyses for cases with multiple tumours (K114, K324, K354, K097, k265) we used the higher stage (or if stage was equal, then the larger of the two tumours, namely: K114_L, K334_R, K352_1, K097_L, K265_1.

##### Classification of disease progression pattern for metastatic cases.

Patterns of disease progression (Table S1) were classified as follows (1) Rapid- disease progression with multiple new lesions or cancer-specific death within 6 months of surgery (2) Attenuated- no disease progression (for example completely resected metastases at presentation, remains disease-free); disease progression with a single new lesion within 6 months of surgery (for example a solitary bone, brain or lung deposit) OR disease progression after >6 months of surgery.

### Method Details

#### Sample collection

All surgically resected specimens were reviewed macroscopically by a pathologist to guide multi-region sampling for this study and to avoid compromising diagnostic requirements. Tumour measurements were recorded and the specimen were photographed before and after sampling. Primary tumours were dissected along the longest axes and spatially separated regions sampled from the “tumour slice” using a 6 mm punch biopsy needle. The punch was changed between samples to avoid contamination. The total number of samples obtained reflects the tumour size with a minimum of 3 biopsies that are non-overlapping and equally spaced. However, areas which are obviously fibrotic or haemorrhagic are avoided during sampling and every attempt is made to reflect macroscopically heterogeneous tumour areas. Primary tumour regions are labelled as R1, R2, R3… Rn and locations are recorded. Normal kidney tissue was sampled from areas distant to the primary tumour and labelled N1. Each biopsy was split into two for snap freezing and formalin fixing respectively, such that the fresh frozen sample has its mirror image in the formalin-fixed sample which is subsequently paraffin embedded. Fresh samples were placed in a 1.8 ml cryotube and immediately snap frozen in liquid nitrogen for >30 seconds and transferred to -80 C for storage. Peripheral blood was collected at the time of surgery and processed to separate buffy coat.

#### Nucleic acid isolation from tissue and blood (TRACERx Renal cohort)

DNA and RNA were co-purified using the AllPrep DNA/RNA mini kit. (Qiagen). Briefly, a 2mm^3^ piece of tissue was added to 900ul of lysis buffer and homogenised for five seconds using the TissueRaptor (Qiagen) with a fresh homogenisation probe being used for each preparation. Each lysate was applied to a QiaShredder (Qiagen) and then sequentially purified using the DNA and RNA columns according to the manufacturer’s protocol. Germline control DNA was isolated from whole blood using the DNeasy Blood and Tissue kit (Qiagen) according to the manufacturers protocol. DNA quality and yield was measured and accessed using the TapeStation (Agilent) and Qubit Fluorometric quantification. (ThermoFisher Scientific)

#### Detection of *VHL* mutations by Sanger sequencing

Validation of the patient *VHL* mutations was carried using PCR followed by Big Dye Terminator Sanger sequencing on the ABI 3700. 20ng of patient DNA was amplified for each *VHL* exon. PCR conditions involved 35 cycles of denaturation at 95^0^C, followed by oligonucleotide primer annealing at 55^o^C and sequence extension at 72^0^C using Qiagen Taq polymerase and reagents. See Data S1 for Oligonucleotide sequences

#### Methylation specific PCR

Methylation of the *VHL* promoter was detected after bisulphite treatment of 500ng of patient DNA using the EZ DNA Methylation-Direct kit (Zymo Research). Bisulphite treated DNA was amplified in the PCR using methylation specific oligonucleotides followed by Big Dye terminator Sanger sequencing. Methylation was confirmed by comparing and contrasting patient tumour and normal renal tissue for methylation protected CpG sequences. See Data S1 for oligonucleotide sequences

#### Independent pathology review of individual tumour regions

Where available, (median of 7 regions per patient (range: 1-63) from 79 patients) histological sections of each region in each case were evaluated by the same pathologist (JIL). Tumor type was assigned to each case following current classification of the International Society of Urologic Pathology (ISUP) (Srigley et al., 2013). Four main histological types were considered based only on hematoxylin-eosin sections: clear cell renal cell carcinoma, papillary renal cell carcinoma, chromophobe renal cell carcinoma and renal oncocytoma. Atypical cases, including unclassified and tumours with mixed histology, were specifically annotated. Tumor architecture was also considered. The presence of rhabdoid and syncytial (Przybycin et al., 2014, Williamson et al., 2014) cells in any region of tumours were also considered, since both are related to a more aggressive clinical course. Tumour grading was performed according to the most up to date ISUP classification (Delahunt et al., 2013) and the presence of necrosis sarcomatoid changes and microvascular invasion was noted. Percentage of viable tumour cells was also estimated in every sample to provide an approximate percentage of tumour content.

#### Regional staining by Immunohistochemistry and Digital Image Analysis of Ki67

Tissue sections of 4μm were mounted on slides and immunohistochemical staining for Ki67 was performed using a fully automated immunohistochemistry (IHC) system and ready-to-use optimized reagents according to the manufacturer's recommendations (Ventana Discovery Ultra, Ventana, Arizona, USA). Primary antibody used was rabbit anti-Ki67 (AB16667, Abcam, Cambridge, UK) and secondary antibody was Discovery Omnimap anti-rabbit HRP RUO (760-4311, Roche, Rotkreuz, Switzerland). DAB kit was Discovery Chromomap DAB RUO (760-4311, Roche). After IHC procedure, slides were first evaluated for Ki67 staining quality using mouse intestine tissue as positive control. Regions containing tumor tissue were identified and marked by a pathologist and subsequently scanned in brightfield at 20x magnification using Zeiss Axio Scan.Z1 and ZEN lite imaging software (Carl Zeiss Microscopy GmbH, Jena, Germany). Digital images were then subjected to automated image analysis using StrataQuest version 5 (TissueGnostics, Vienna, Austria) for Ki67 quantification. Three different gates were set to quantify low, medium and high intensity DAB staining which corresponded to Ki67 expression levels. Results were depicted as total percentage of Ki67-positive nuclei.

#### Flow Cytometry Determination of DNA Content (FACS)

Fresh frozen tumour tissue samples, approximately 4mm^3^ in size, were mechanically disrupted and incubated in 2ml of 0.5% pepsin solution (Sigma, UK) at 37 ºC for 40 minutes to create a suspension of nuclei. The nuclei were washed with phosphate-buffered saline (PBS) and then fixed with 70% ethanol for a minimum of 90 minutes. The nuclei were washed again with PBS and stained with 200μl of propidium iodide (50μg/ml) overnight. Flow cytometric analysis of DNA content was performed using the LSR Fortessa Cell Analyzer (Becton Dickinson, San Jose, USA), BD Facs Diva™ software and FlowJo software (FlowJo LLC, Oregon, USA. A minimum of 10,000 events were recorded (typically up to 20,000 and up to 100,000 in complex samples). Analysis was performed using methods derived from the European Society for Analytical Cellular Pathology DNA Consensus in Flow Cytometry guidelines and following discussions with Derek Davies (Head of Flow Cytometry Facility, The Francis Crick Institute). Gating of forward and side scatter was applied to exclude debris and cell clumping. Samples with <7,500 events after gating were excluded from further analysis. The coefficient of variation (CV) was measured on each G1 peak. Samples with a CV>10% were excluded from further analysis. Each tumour sample was assumed to contain normal cells to act as internal standard. Where possible the position of the diploid peak was calculated with reference to the peak of diploid cells in a case matched normal tissue sample. The DNA index (DI) of any aneuploid peak present was calculated by dividing the G1 peak of the aneuploid population by the G1 peak of the normal diploid cells. Diploid samples were defined as having DI of 1.00. Any additional peak was defined as aneuploid. A tetraploid peak was defined as having a DI of 1.90-2.10 and containing >15% of total events unless a second peak corresponding to G2 was clear on the histogram. Similarly, aneuploid peaks near to G1 (DI 0.90-1.10) were only considered if there was a clear second peak containing >15% of total events.

#### Targeted Driver Panel (DP) design and validation

Driver gene panels (Panel_v3, Panel_v5 and Panel_v6) were used in this study. Panel_v3 was designed in 2014, including 110 putative driver genes. Panel_v5 and Panel_v6 were designed in 2015, including 119 and 130 putative driver genes respectively. Driver genes were selected from genes that were frequently mutated in TCGA (accessed in April 2015) or highlighted in relevant studies (Arai et al., 2014, Sato et al., 2013, Scelo et al., 2014). Only alterations in driver genes represented in all three panels were considered in the overall driver mutation analyses. All panels targeted potential driver SCNA regions. To prevent inter-patient samples swaps, we included the 24 SNPs that were previously identified by Pengelly et al. (2013) in Panel_v5 and Panel_v6. Details of the 3 panels can be found in Supplementary table (Table S2).

#### Driver Panel Library Construction and Targeted Sequencing

Following isolated gDNA QC, depending on the available yield, samples were normalised to either 1-3 μg or 200 ng for the Agilent SureSelectXT Target Enrichment Library Protocol; standard or low input sample preparation respectively. Samples were normalised using a 1X Low TE Buffer. Samples were sheared to 150-200bp using a Covaris E220 (Covaris, Woburn, MA, USA), following the run parameters outlined in the Agilent SureSelectXT standard 3 μg and low input 200 ng DNA protocols. Library construction of samples was then performed following the SureSelectXT protocols, using 6 pre-capture PCR cycles for the standard input samples and 10 pre-capture PCR cycles for the 200 ng low input samples. Hybridisation and capture were performed for each individual sample using the Agilent custom Renal Driver Panel target-specific capture library (versions 3, 5 & 6). The same version of the capture library being used for all samples from the same patient case. Captured SureSelect-enriched DNA libraries were PCR amplified using 14 post-capture PCR cycles in PCR reactions that included the appropriate indexing primer for each sample. Amplified, captured, indexed libraries passing final QC on the TapeStation 4200 were normalised to 2nM and pooled, ensuring that unique indexes were allocated to all final libraries (up to 96 single indexes available) in the pool. QC of the final library pools was performed using the Agilent Bioanalyzer High Sensitivity DNA Assay. Library pool QC results were used to denature and dilute samples in preparation for sequencing on the Illumina HiSeq 2500 and NextSeq 500 sequencing platforms. The final libraries were sequenced 101bp paired-end multiplexed on the Illumina HiSeq 2500 and 151bp paired-end multiplexed on the NextSeq 500, at the Advanced Sequencing Facility at the Francis Crick Institute. Equivalent sequencing metrics, including per sample coverage, was observed between platforms.

#### Whole Exome Library Construction and Sequencing

gDNA isolated from each sample were normalized to 1-3 μg. Libraries were prepared from using the Agilent SureSelectXT Target Enrichment Library protocol and Agilent SureSelectXT Human All Exon v4 enrichment capture library. The libraries were prepared using 6 pre-capture and 12 post-capture PCR cycles. Captured Whole Exome final libraries passing the final QC step were normalised to 2nM and pooled for sequencing on the HiSeq 2500 instrument. Dual HiSeq SBS v4 runs at 101bp paired-end reads generated the data for analysis. Target coverage was 400-500x for the tumour regions and 100-200x for the associated normal.

#### SNV, and INDEL calling from multi-region DP and multi-region WE sequencing

Paired-end reads (2x100bp) in FastQ format sequenced by Hiseq or NextSeq were aligned to the reference human genome (build hg19), using the Burrows-Wheeler Aligner (BWA) v0.7.15. with seed recurrences (-c flag) set to 10000 (Li and Durbin, 2009). Intermediate processing of Sam/Bam files was performed using Samtools v1.3.1 and deduplication was performed using Picard 1.81 (http://broadinstitute.github.io/picard/) (Li and Durbin, 2009). Single Nucleotide Variant (SNV) calling was performed using Mutect v1.1.7 and small scale insetion/deletions (INDELs) were called running VarScan v2.4.1 in somatic mode with a minimum variant frequency (--min-var-freq) of 0.005, a tumour purity estimate (--tumor-purity) of 0.75 and then validated using Scalpel v0.5.3 (scalpel-discovery in - -somatic mode) (intersection between two callers taken) (Cibulskis et al., 2013, Fang et al., 2016, Koboldt et al., 2009). SNVs called by Mutect were further filtered using the following criteria: i) ≤5 alternative reads supporting the variant and variant allele frequency (VAF) ≤ 1% in the corresponding germline sample, ii) variants that falling into mitochondrial chromosome, haplotype chromosome, HLA genes or any intergenic region were not considered, iii) presence of both forward and reverse strand reads supporting the variant, iv) >5 reads supporting the variant in at least one tumour region of a patient, v) variants were required to have cancer cell fraction (CCF)>0.5 in at least one tumour region (see Subclonal deconstruction of mutations section for details of CCF calculation) , vi) variants were required to have CCF>0.1 to be called as present in a tumour region, vii) sequencing depth in each region need to be >=50 and ≤3000. Finally, suspected artefact variants, based on inconsistent allelic frequencies between regions, were reviewed manually on the Integrated Genomics Viewer (IGV), and variants with poorly aligned reads were removed. Dinucleotide substitutions (DNV) were identified when two adjacent SNVs were called and their VAFs were consistently balanced (based on proportion test, *P*>=0.05). In such cases the start and stop positions were corrected to represent a DNV and frequency related values were recalculated to represent the mean of the SNVs. Variants were annotated using Annovar (Wang et al., 2010). Deleterious mutations were defined if two out of three algorithms - SIFT, PolyPhen2 and MutationTaster - predicted the mutation as deleterious. Individual tumour biopsy regions were judged to have failed quality control and excluded from analysis based on the following criteria: i) sequencing coverage depth below 100X, ii) low tumour purity such that copy number calling failed. Mutations detected in high-confidence driver genes (*VHL, PBRM1, SETD2, PIK3CA, MTOR, PTEN, KDM5C, CSMD3, BAP1, TP53, TSC1, TSC2, ARID1A, TCEB1*) were defined as *driver mutations*. As *TSC1* and *TSC2* were not targeted in Panel v5, to check the mutation status in these two genes, patients were sequenced using Panel v5 were re-sequenced with Panel v6 and no new mutations were detected.

#### SCNA calling from multi-region DP and multi-region WE sequencing

To estimate SCNAs, CNVkit v0.7.3 was performed with default parameter on paired tumour-normal sequencing data (Talevich et al., 2016). Outliers of the derived log2-ratio (logR) calls from CNVkit were detected and modified using Median Absolute Deviation Winsorization before case-specific joint segmentation to identify genomic segments of constant logR (Nilsen et al., 2012). Tumour sample purity, ploidy and absolute copy number per segment were estimated using ABSOLUTE v1.0.6 (Carter et al., 2012). In line with recommended best practice all ABSOLUTE solutions were reviewed by 3 researchers, with solutions selected based on majority vote. Copy number alterations were then called as losses or gains relative to overall sample wide estimated ploidy. Arm gain or loss was called when >50% of the chromosomal have copy number gain or loss. Driver copy number was identified by overlapping the called somatic copy number segments with putative driver copy number regions identified by Beroukhim and colleagues (Beroukhim et al., 2009). We compared SCNA calls between targeted panel and WGS datasets, and SCNA concordance was 87% (Table S2). The average proportion of the genome with aberrant copy number, weighted on each of the 22 autosomal chromosomes, was estimated as the weighted genome instability index (wGII).

#### TCGA WES data analysis

To compare mutation frequency detected in TRACERx Renal cohort with public data (Figures 1B and 1C), event calls from 451 TCGA KIRC patients were retrieved from cBioportal (http://www.cbioportal.org/) on 2017/07/21. To investigate the clonality of mutations in TCGA KIRC cohort, we obtained the next generation sequencing data for matched tumour and normal/blood from 338 cases in BAM format from TCGA, which were then converted into FASTQ format files using bam2fastq in bedtools package (Quinlan and Hall, 2010). SNVs, INDELs and SCNAs were called using the same methods as TRACERx Renal data (STAR Methods: SNV, and INDEL calling from multi-region DP and multi-region WE sequencing, SCNA calling from multi-region DP and multi-region WE sequencing). 20 cases were excluded from the study as the ABSOLUTE v1.0.6 algorithm failed to find a stable SCNA solution, further details can be found in Table S4. Clonality of SNVs and SCNAs were estimated using ABSOLUTE v1.0.6. Cancer cell fraction for INDELs were calculated using method described in STAR Methods: Subclonal deconstruction of mutations. INDELs with CCF>0.5 were called clonal. ITH index for each patient was calculated as the measure of intratumour heterogeneity (ITH index = # subclonal drivers / # clonal drivers). However, due to the limitation of single biopsy, intratumour heterogeneity was found to underestimated (ITH index range 0-3, median=0.0, sd=0.41).

### Quantification and Statistical Analysis

R 3.3.2 was used for all statistical analyses.

#### Saturation Analysis and Phenotypic Correlations

For saturation analysis, the mean number of variants observed for each subset of biopsies of a given size was calculated by exhaustive consideration of all such subsets when the total number of such subsets was less than 18 million and by consideration of a random collection of 18 million subsets, with possible repetition, when the total number of possibilities was greater. For phenotypic correlations, comparisons were performed using the Fisher's Exact test for 2x2 tables and the "non-parametric 2-way anova" Freidman test for n x m tables where at least one of n and m is greater than 2. P-values were corrected for multiple testing using the Benjamini–Hochberg procedure.

#### Subclonal deconstruction of mutations

To estimate the clonality of a mutation in a region, we used the following formula:

$$vaf=\frac{CN_{mut}\;*\;CCF\;*\;p}{CN_{n}\;*\;\left(1-p\right)+\;CN_{t}\;*\;p}$$

where vaf is the variant allele frequency at the mutation base; p is estimated tumour purity; $CN_{t}$ and $CN_{n}$ are the tumour locus specific copy number and the normal locus specific copy number which was assumed to be 2 for autosomal chromosomes; and CCF is the fraction of tumour cells carrying the mutation. Consider $CN_{mut\;}$is the number of chromosomal copies that carry the mutation, the possible $CN_{mut\;}$ is ranging from 1 to $CN_{t}$ (integer number). We then assigned CCF with one of the possible value: 0.01, 0.02, ..., 1, together with every possible $CN_{mut\;}$ to find the best fit cancer cell fraction of the mutation. Since we focused on driver genes in this study and the accuracy of the estimated CCF is limited by the size of the panel, we call mutations with CCF>0.5 as clonal mutations, mutations with CCF≤0.5 and CCF>0.1 are subclonal. To determine the clonality of a mutation in a tumour, we ask for the mutation to be clonal in all regions in a tumour. Exceptions were made for long INDELs that affect >6 bp of the genome, due to VAF under estimation. If a long INDEL is present in all regions of a tumour, we called it as clonal. To determine the clonality of a SCNA in a tumour, we ask for the SCNA to be presence in all tumour regions, otherwise it is called subclonal.

#### Driver tree reconstruction

A matrix with presence and absence of nonsynonymous and synonymous point mutations, DNVs, INDELs and arm level SCNAs was created for each tumour, and all the events were clustered based on the following rule: a valid cluster has to have at least two arm level SCNAs or one non-synonymous mutation. The driver events clusters were then ordered into a clonal hierarchy using TRONCO and presented as driver trees (De Sano et al., 2016).

Clustering was performed on multi-region whole exome sequencing using PyClone Dirichlet process clustering (Roth et al., 2014). For each mutation, the observed variant count was used and reference count was set such that the VAF was equal to half the pre-clustering CCF. Given that copy number and purity had already been calculated, we set the major allele copy numbers to 2 and minor allele copy numbers to 0 and purity to 0.5; allowing clustering to simply group clonal and subclonal mutations based on their pre-clustering CCF estimates. PyClone was with 10,000 iterations and a burn-in of 1000, and default parameters, with the exception of --var_prior set to ‘BB’ and –ref_prior set to ‘normal’.

In terms of limitations, we recognise that our Driver Panel phylogenies are based on fewer clonal markers, as compared to whole exome or genome derived phylogenetic trees. As a consequence some tumour clones are based on only a limited number of genomic markers, however three contingency measures are in place to mitigate against phylogenetic misconstruction: i) ultra-deep 500x sequencing coverage, which ensures stably derived cancer cell fraction estimates, ii) a bespoke gene panel which is enriched for driver events, increasing the likelihood that mutational markers are driving genuine clonal expansion, iii) cross-capture validation with tree structures in >10 cases confirmed using exome sequencing data (Table S5). Furthermore, the panel sequencing strategy has allowed extensive tumour sampling, with >1,200 biopsies sequenced, enabling robustness in terms of spatial sampling.

#### Parallel evolution significance testing

All genes with ≥ 10 subclonal mutations across the cohort were tested for evidence of parallel evolution (qualifying genes: *BAP1, CSMD3, KDM5C, MUC16, MTOR, PBRM1, PTEN, SETD2. TSC1, TP53*). For each gene the observed number of parallel mutations across the 100 case cohort was compared to a null distribution of the expected number of subclonal mutations co-arising in different tumour regions within the same case due to chance. To simulate the null distribution the mutation frequency of each gene per biopsy region was calculated, based on total number of unique subclonal mutations for that gene (cohort wide) divided by the total number of biopsies sequenced (cohort wide). This probability was then used in a simple Bernoulli trials model simulated for each patient, with the number of trials based on the number of biopsy regions sequenced per case. This model allows for the fact that cases with a large number of sampled regions have high chance of co-arising mutations in different biopsy regions by chance rather than due to parallel evolution. The total count of co-arising mutations by chance was calculated across the 100 case cohort (using the specific number of biopsy regions per case) and then compared to the observed number parallel events. Significance was determined through 1000 permutations per gene, with resulting p-values corrected for multiple testing using the Benjamini–Hochberg procedure.

#### Detection of allelic imbalance

Heterozygous SNPs called using germline variants were identified using VarScan v2.4.1 in mpileup2snp mode. SNPs used must be called in all regions of the tumour and have a B-allele frequency (BAF, total variant base / total reference bases at a position) of between 0.35 and 0.65 in the germline sample. Mean absolute deviation (MAD) from 0.5 calculated for all heterozygous SNPs on each arm in all samples: mean (abs(arm_hz_BAF – 0.5)). The germline MAD was then subtracted from all tumour region MADs for each patient’s disease for all chromosome arms. Copy neutral allelic imbalance was then called if: 1) There is no copy number event (gain or loss) associated with the chromosome arm in a sample but there is a MAD of >= 0.1. 2) There is no copy number event associated with the chromosome arm in a sample but its MAD is >= the median MAD of gain/loss events in this sample and is also >= 0.05. 3) If a patient’s disease has the same chromosome arm exhibiting copy neutral allelic imbalance in 2 or more regions by the above the two criteria, the same chromosome arm in the other regions is re-examined using the lowest quartile MAD of gain/loss events in each region as a cut off and has a MAD of >=0.05.

Calculating clonality of copy neutral allelic imbalance (CNAI): Only regions with at least one chromosome arm exhibiting a MAD score of greater than 0.05 were considered for this analysis. Regions with no MAD score greater than 0.05 are marked on the patient specific supplementary figures “low purity” (Data S3). Copy neutral allelic imbalance calls are shown as diamonds in the patient specific copy number plots attached in this email. The CNAI occurrences in each patient were then grouped into the following categories: Clonal CNAI – All regions of the tumour have no copy number gains or losses associated with this chromosome arm but all have been classified as exhibiting CNAI. Clonal loss and CNAI – All regions of the patient’s disease have either a loss being called or exhibit CNAI for this chromosome arm.

#### Detection of mirrored subclonal allelic imbalance (MSAI)

In order to detect mirrored subclonal allelic imbalance (MSAI) allele counts were generated using AlleleCounter (https://github.com/cancerit/alleleCount) (see companion paper Mitchell et al., 2018). The counts from whole exome sequenced samples were analysed using ASCAT (Van Loo et al., 2010) to generate copy number calls. Whole-genome samples were analysed using Battenberg (Nik-Zainal et al., 2012) to generate copy number calls (see companion paper, Mitchell et al., 2018). Heterozygous SNPs among the 1000 genomes positions (Abecasis et al., 2010) used as input for ASCAT/Battenberg analyses were identified by isolating those which had a B-allele frequency (BAF) of between 0.3 and 0.7 (calculated by variant reads over total reads) in the germline sample for each patient. The BAFs of these heterozygous SNPs were then used with the segmentation and copy number calls produced for each region by either ASCAT or Battenberg analyses to detect MSAI events for each patient’s disease using the method outlined previously (Jamal-Hanjani et al., 2017).

Using the heterozygous SNPs present in the targeted regions detected by Driver Panel sequencing we identified allelic imbalance (AI) at the level of chromosome arms. In some cases the AI was not associated with a copy number gain or loss relative to the sample’s ploidy and was classified as copy neutral allelic imbalance (CNAI) (STAR Methods). In total, we identified 18 cases where one or more chromosome arms demonstrated clonal CNAIs (34 events total) and 8 patients where, at least one chromosome arm was always affected by either loss relative to ploidy or CNAI (13 events total). 5 of these 8 patients also demonstrated instances of ubiquitous arm level CNAI in all regions.

#### Validation of MSAI

Validation of MSAI was achieved using Polymorphic microsatellite markers specific to the chromosome and chromosome region being investigated. Once a polymorphic marker is identified, patient DNA is amplified in the PCR, incorporating a fluorescent primer into the PCR fragment that can be accurately measured for size and fluorescent intensity. Measurement of Fluorescent units under each allele peak can be used to compare and contrast variation between alleles within and between different tumour regions and the normal sample using the formula (At/Bt)/(An/Bn).

#### Co-occurrence testing

Co-occurrence of driver events in each tumour was conducted based on the driver tree clones as determined above. Analysis was conducted on the most frequent driver mutational events (*BAP1, PBRM1, SETD2, VHL*, Figure 1B*)*, the most frequent SCNAs (3p loss, 5q gain) and SCNA events with established clinically prognostic value (loss 4q, loss 9p, loss 14q and gain 8q) (Ito et al., 2016, Kojima et al., 2009, La Rochelle et al., 2010, Monzon et al., 2011, Perrino et al., 2015). For each event pairing tumour clones were assessed to determine if the given two events were found to co-occur together in the same clone. Analysis was first conducted using only the “MRCA” clone per case (n=100), to ensure independence of observations at the patient level (for bilateral/multi-focal cases the first/left tumour was taken in each case). Analysis was then repeated using “MRCA plus subclonal” clones (total n=306 across all tumors, with the set of subclones defined as unique terminal tree nodes). R package ‘cooccur’ (Griffith et al., 2016)was used to compare observed event co-occurrence frequencies to those expected by chance under a probabilistic model. The distribution of observed and expected values is shown in Figure S2. Values were plotted as enrichment scores calculated as log_2_(observed count/expected count). Only patterns found to be significant in both the “MRCA” and “MRCA plus subclonal” were considered significant overall. Correction for multiple testing was conducted using the Benjamini–Hochberg procedure.

#### Most recent common ancestor (MRCA) and ki67 analysis

The estimated time of MRCA was calculated using multi-region whole genome sequencing data as detailed in the companion paper by Mitchell et al. (2018). From the total n=33 cases with WGS data, MRCA timing analysis was successful in n=31 cases, from which known *VHL* wildtype cases (n=2) were excluded on account of their distinct aetiological and phenotypic profile. Of the n=29 cases analysed, n=23 overlapped with the renal TRACERx Renal 101 cohort presented here, and n=6 were additional ccRCC patients recruited to the TRACERx Renal study. The association between time from MRCA to tumour diagnosis and number of clonal driver events was assessed using a linear model, adjusting for the total clonal mutation burden per tumour. The association between tumour region ki67 % of cells stained as positive and number of clonal driver events was assessed using a linear mixed effect (LME) model, to account for the non-independence of multiple samples from individual patients, using all cases with available data in the TRACERx Renal 101 cohort after exclusion of known *VHL* wildtype tumours.

#### Event ordering analysis

The ordering of driver events was based on the clonal hierarchy of each tumour, as determined by driver tree reconstruction method detailed above. Due to dense spatial sampling (median 7 biopsies per tumour, range [3-75]) the driver tree ordering was typically robust, with evidence of sequential waves of clonal expansion between events usually confirmed across multiple biopsy regions. The set of sequential event paths (*i.e.* event A > event B > event C) for each tumour was captured starting with the events in the MRCA clone. For each MRCA event, evolutionary sequences were traced through each node of the tree until a terminal clone was reached. All possible sequential paths (trajectories) between MRCA and terminal clone events were recorded. To reduce risk of multiple testing we limited further analyses to those trajectories containing the most frequent (“core”) ccRCC driver events: *VHL, PBRM1, BAP1, SETD2,* PI3K/AKT/mTOR pathway mutations or driver SCNAs. The list of trajectories was further reduced to ensure pairings of events were counted only once per case, (e.g. in the case of K243 where a single *PBRM1* mutation precedes 10 *SETD2* mutations, this is counted only once) and PI3K/AKT/mTOR pathway mutations interacting with SCNAs were not considered due to the nonspecific many-to-many relationship. The final list of trajectories was analysed using R package Trajectory Miner (Gabadinho et al., 2011) to identify recurrent patterns of event pairs enriched for occurrence is a consistent direction. Event pairings observed in ten or more cases were then tested for significance in a specific ordering direction using a Binomial test, with null expected p=0.5, to reflect an equally balanced 50%:50% distribution of event ordering by random chance. As expected, *VHL* was found to be significantly enriched as an early event preceding all other alterations, consistent with its known timing as a universally clonal event (data not shown in figure). All p-values were corrected for multiple testing using the Benjamini–Hochberg procedure.

#### Evolutionary subtype classification

Based on the evolutionary analysis in Figures 4 a rule based classification was devised in order to assign cases into subgroups and allow for comparison against phenotypic and clinical outcomes. Cases were assigned to groups based on the following series of rules (applied in a hierarchical manner in the order listed): i) presence of ≥ 2 *BAP1, PBRM1, SETD2* or *PTEN* clonal mutational events meant assignment to “multiple clonal driver” group (the selection of these four genes is based on the timing results observed in Figure 4B) , ii) presence of a tumour clone/subclone with a *BAP1* mutational driver event, and no other “core” mutational driver events aside from *VHL* in that same clone/subclone, meant assignment to the “*BAP1* driven” group, iii) presence of a tumour clone/subclone with *PBRM1* mutation followed by a *SETD2* mutation, meant assignment to the “*PBRM1*->SETD2” group, iv) presence of a tumour clone/subclone with *PBRM1* mutation followed by a PI3K pathway mutation, meant assignment to the “*PBRM1->*PI3K” group, v) presence of a tumour clone/subclone with *PBRM1* mutation followed by a driver SCNA event, meant assignment to the “*PBRM1*->SCNA” group, vi) absence of *VHL* mutation or methylation meant assignment to “*VHL* wildtype” group, vii) presence of *VHL* as the only “core” mutational driver event meant assignment to the “*VHL* monodriver” group. For bilateral/multi-focal cases the evolutionary subtype was assigned based on the first/left tumour in each case. To test the stability and validity of the rule based classification an unsupervised clustering analysis was additionally performed, using R function daisy, with the distance matrix computed using Gower’s formula on account of the mixture of continuous and binary data types. Clustering was conducted based on the following measures: wGII (minimum and maximum regional values per tumour), tumour size (mm), clone number, ITH index, number of clonal driver events and presence/absence of the six observed evolutionary patterns (*BAP1* lone driver clone/subclone, *PBRM1->SETD2* clone/subclone, *PBRM1->*PI3K clone/subclone, *PBRM1->*SCNA clone/subclone, *VHL* mutational status, *VHL* as the only “core” mutational driver event). Clustering was performed using a partitioning around medoid method, with cluster number from 2 to 15 considered, and a 10 cluster solution resulting as the optimal solution. Overall high concordance in cluster assignment was observed between the rule based and unsupervised methods, and in the unsupervised method three additional subgroups were identified (Figure S3, the groups are referred to just by cluster number due to currently unclear evolutionary aetiology): cluster 5 which was characterised by low clone number (median=2) and small size (mean=6.7cm), cluster 7 which exhibited high wGII, and cluster 9 with branched structure (median 11 clones) and large size (mean=10.9cm).

#### Survival analysis

Survival analysis was conducted using the Kaplan-Meier method, with p-value determined by a log-rank test. Progression free survival (PFS) was defined as the time to recurrence or relapse, or if a patient had died without recurrence, the time to death. In the TRACERx cohort, overall survival (OS) was measured as cancer specific death. For the TCGA cohort, all death events were included in the PFS/OS analyses (consistent with the original author’s analysis of the data, on account of a lack of cause of death data). Hazard ratio and multivariate analysis adjusting for clinical parameters was determined through a Cox proportional hazards model.

#### Downsampling simulation

Empirical error rates were determined by exhaustive consideration of all pairs of biopsies from a given tumour sample and, for each pair, comparing the number of variants detected in one or more of the full set of biopsies not found in either member of that pair ("False negative") or determined to be subclonal in the full set but detected in both samples in that pair ("illusion of clonality"). Each tumour is then represented by the mean value of each of these estimates across all pairs. We acknowledge that, despite dense sampling, the variant set found across all biopsies per tumour clearly may also be missing very rare low frequency driver events itself.

### Data and Software Availability

The accession number for the Sequencing data reported in this paper is European Genome-Phenome Archive (EGA) hosted by the European Bioinformatics Institute (EBI): EGAS00001002793. Additional genomic and clinical data are provided via this link: https://bitbucket.org/tracerxrenal/cell-paper-data-2018/src.

### Additional Resources

Clinical trial registry number: https://clinicaltrials.gov/ct2/show/NCT03226886

TRACERx Renal study website, detailing investigators, sponsors and collaborators: http://TRACERxRenal.co.uk/studies/renal/

## Consortia

The members of TRACERx Renal Consortium are: Tim O’Brien, David Nicol, Ben Challacombe, Archana Fernando, Steve Hazell, Ashish Chandra, Jose I. Lopez, James Larkin, Martin Gore, Lisa Pickering, Sarah Rudman, Simon Chowdhury, Karen Harrison-Phipps, Mary Varia, Catherine Horsfield, Alexander Polson, Gordon Stamp, Marie O’Donnell, William Drake, Peter Hill, David Hrouda, Eric Mayer, Jonathon Olsburgh, Gordon Kooiman, Kevin O’Connor, Michael Aithcison, Maxine Tran, Nicos Fotiadis, Hema Verma, and Grant Stewart.

## Supplemental Information

Table S1. TRACERx Renal Cohort: Clinical Characteristics, Evolutionary Subtypes, Progression Patterns, and Survival Outcomes, Related to Figure 1

Table S2. TRACERx Renal Driver Panels and Detected Somatic Alternations, Related to Figure 1 and STAR Methods

Table S3. TRACERx Renal Cohort: Multiregional Ki67 Immunohistochemistry Staining Analysis, Related to Figure 4

Table S4. TCGA KIRC Cohort: Multivariate Survival Analysis and Processing Notes, Related to Figure 7 and STAR Methods

Table S5. Comparison of Clone Numbers Detectable from Driver Panel and Whole Exome Sequencing in the Same Cases, Related to STAR Methods The left panel shows the raw clone numbers for each case, per sequencing data type. The right panel shows the correlation of number of tumor clones identified using Renal Driver panel (x axis) and whole exome sequencing (y axis). The shaded area represents the confidence interval.

Data S1. TRACERx Renal Cohort: VHL Summary, Related to Figure 1 and STAR Methods *VHL* mutations as confirmed by Sanger sequencing, and *VHL* methylation results.

Data S2. Mutation Heatmap and Driver Trees for a Subset of TRACERx Renal Cohort, Related to Figures 1 and 2 Mutation heatmap and driver trees, for individual cases as referenced in the Results section.

Data S3. Copy Neutral Allelic Imbalance, MSAI, and SCNA Profiles for Bilateral and Multifocal Tumors, Related to Figures 1 and 3 and STAR Methods Page 1 shows copy neutral allelic imbalance data. Pages 2-17 shows case level MSAI results. Page 18 shows a summary of MSAI results. Pages 19-20 shows MSAI validation results. Page 21 shows SCNA profiles for bilateral and multifocal cases, with each row represents an individual tumour region; blue represents copy number loss, red represents copy number gain.

Data S4. TRACERx Renal Cohort: Correlation of Driver Events Versus Clinical Variables and Tumor Size versus Number of Clones, Related to Figure 1 and STAR Methods Shown on page 1 are boxplots illustrating comparison of number of variants, ITH score or number of clones classified variously by Tumour Size (in cm), Overall Stage, Grade and Tumour Necrosis status. Drivers refer to "driver events", i.e. non-synonymous SNVs, DNVs, or small INDELS (Muts) in "Driver Genes" or driver copy number events (SCNA). Clonal/Subclonal Drivers refer to events detected in all/not-all primary regions respectively. Driver ITH refers to the ITH index (# of subclonal variants/# of clonal variants) restricted to driver events. P-values refer to pairwise comparisons of groups as indicated and were performed using a Wilcoxon test in all cases. n values indicate the size of the baseline group when no p value is presented, or the comparison group when associated with a p value. Non-significant p values are included for completeness. Shown on page 2 are local polynomial curve fits (using the locpoly function in the R package KernSmooth) and display the results: rows correspond to the fitting of curves of fixed degree, 1 (i.e. linear) in the first row and increasing by 1 until degree 5 (i.e. quintic) curves are applied in the bottom row. Columns correspond to fixed fit bandwidths, which is to say, the size of the window across which the curve is fit, with window sizes of 1, 5, 10, 15 and 20 applied. These local fits are illustrated with the green curve, while a red, global linear least squares fit is also displayed for comparison.
