# Supplementary figures and images for: Deterministic Evolutionary Trajectories Influence Primary Tumor Growth: TRACERx Renal
Source: Cell. 2018 Apr 19;173(3):595–610.e11. doi: 10.1016/j.cell.2018.03.043 (PMC5938372; doi:10.1016/j.cell.2018.03.043)

| Case | Clones_Exome | Clones_Panel_Matched |
|------|--------------|----------------------|
| K376 | 1            | 1                    |
| K064 | 2            | 1                    |
| K065 | 2            | 1                    |
| K143 | 3            | 2                    |
| K027 | 5            | 3                    |
| K099 | 5            | 3                    |
| K386 | 4            | 4                    |
| K098 | 3            | 7                    |
| K130 | 5            | 4                    |
| K139 | 7            | 4                    |
| K128 | 4            | 5                    |

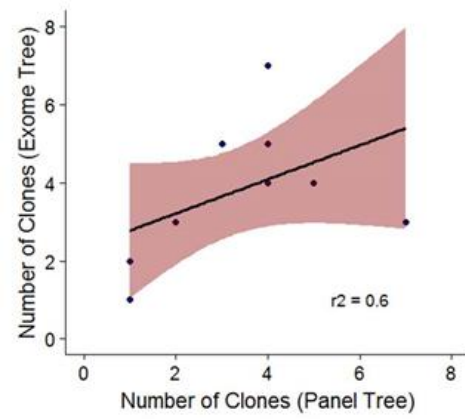

Supplement: Table S5. Comparison of Clone Numbers Detectable from Driver Panel and Whole Exome Sequencing in the Same Cases, Related to STAR Methods — The left panel shows the raw clone numbers for each case, per sequencing data type. The right panel shows the correlation of number of tumor clones identified using Renal Driver panel (x axis) and whole exome sequencing (y axis). The shaded area represents the confidence interval. [file mmc5.pdf]

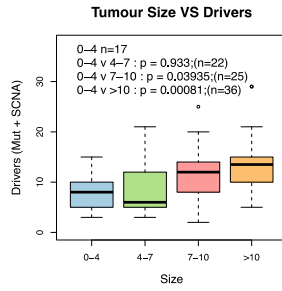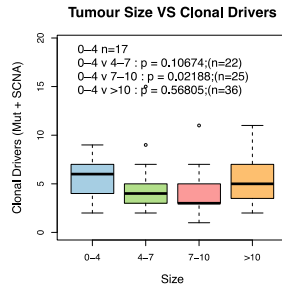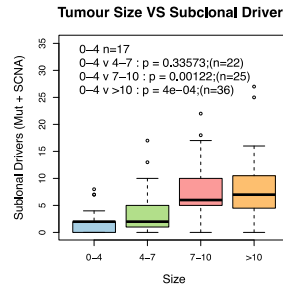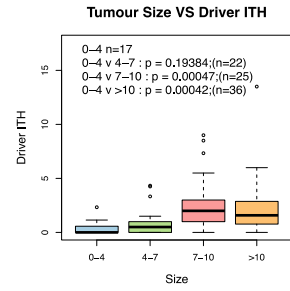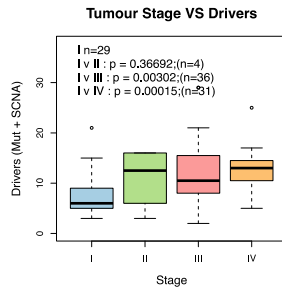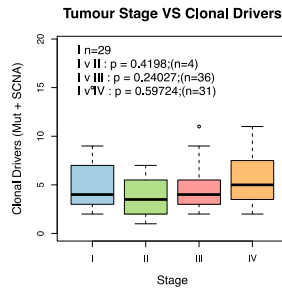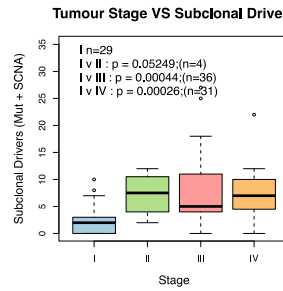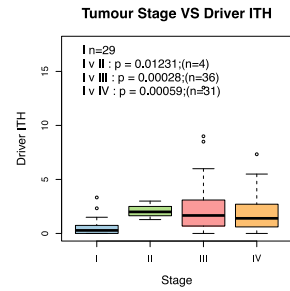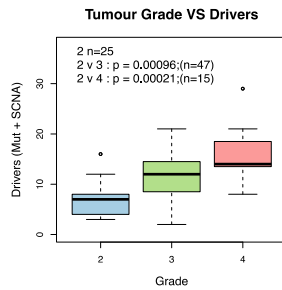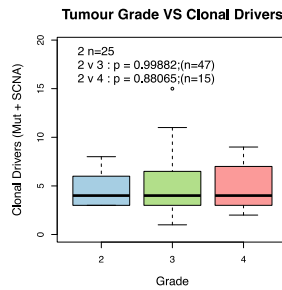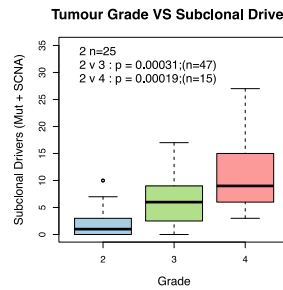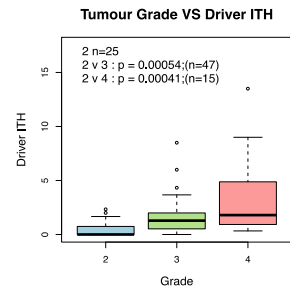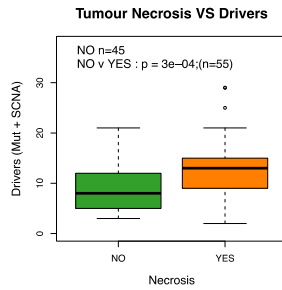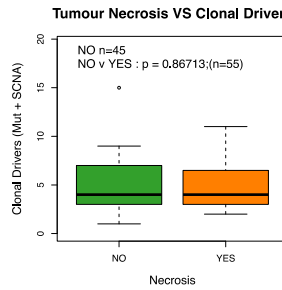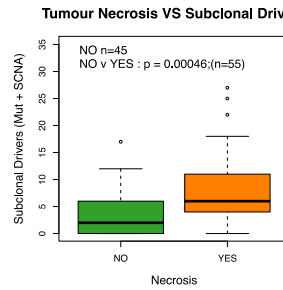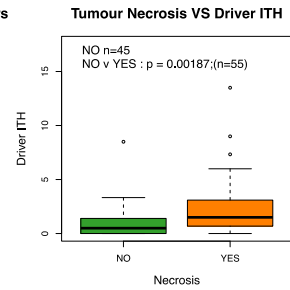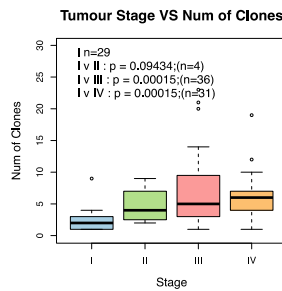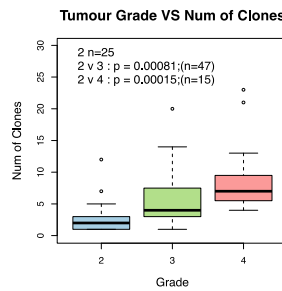

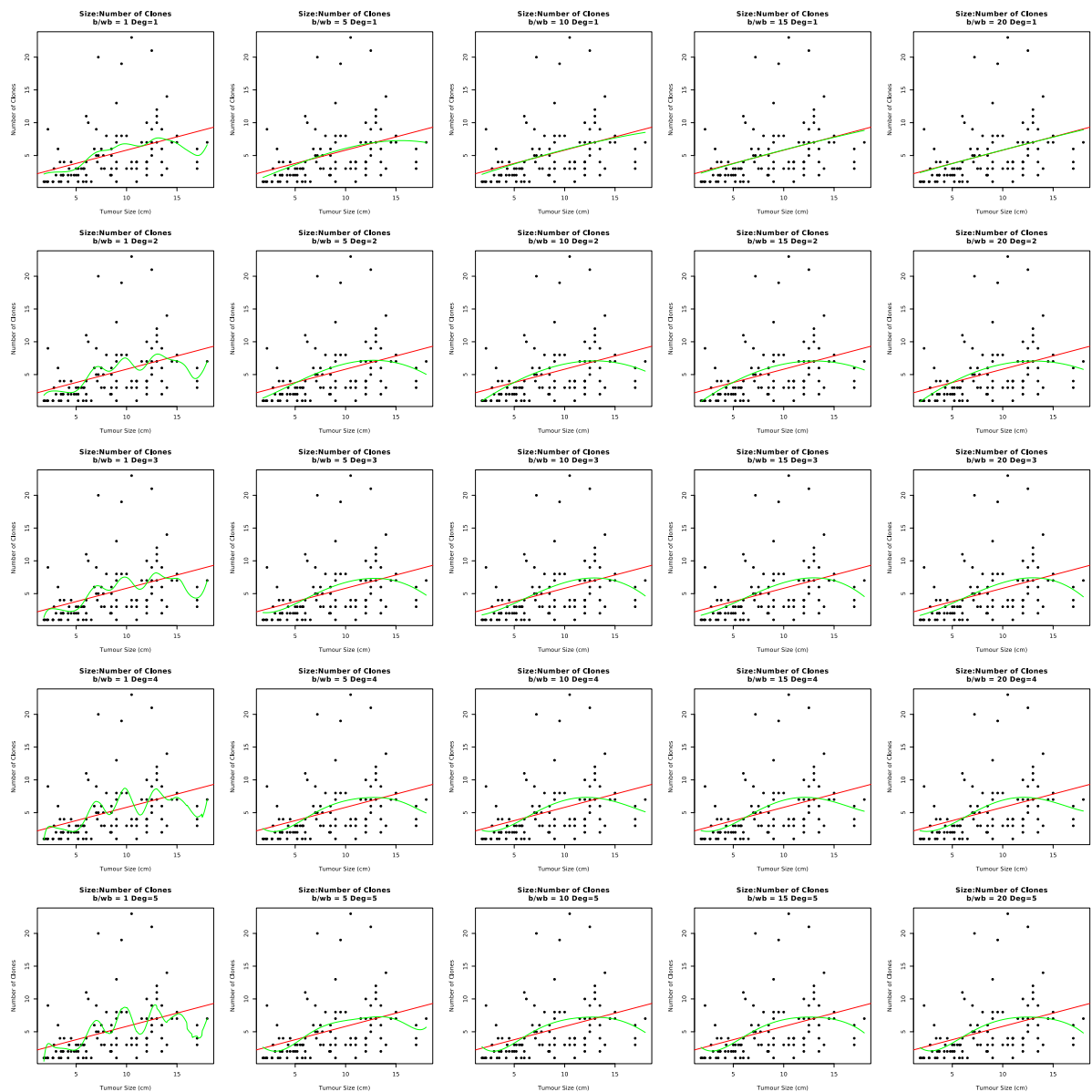

Supplement: Data S4. TRACERx Renal Cohort: Correlation of Driver Events Versus Clinical Variables and Tumor Size versus Number of Clones, Related to Figure 1 and STAR Methods — Shown on page 1 are boxplots illustrating comparison of number of variants, ITH score or number of clones classified variously by Tumour Size (in cm), Overall Stage, Grade and Tumour Necrosis status. Drivers refer to "driver events", i.e. non-synonymous SNVs, DNVs, or small INDELS (Muts) in "Driver Genes" or driver copy number events (SCNA). Clonal/Subclonal Drivers refer to events detected in all/not-all primary regions respectively. Driver ITH refers to the ITH index (# of subclonal variants/# of clonal variants) restricted to driver events. P-values refer to pairwise comparisons of groups as indicated and were performed using a Wilcoxon test in all cases. n values indicate the size of the baseline group when no p value is presented, or the comparison group when associated with a p value. Non-significant p values are included for completeness. Shown on page 2 are local polynomial curve fits (using the locpoly function in the R package KernSmooth) and display the results: rows correspond to the fitting of curves of fixed degree, 1 (i.e. linear) in the first row and increasing by 1 until degree 5 (i.e. quintic) curves are applied in the bottom row. Columns correspond to fixed fit bandwidths, which is to say, the size of the window across which the curve is fit, with window sizes of 1, 5, 10, 15 and 20 applied. These local fits are illustrated with the green curve, while a red, global linear least squares fit is also displayed for comparison. [file mmc9.pdf]
