# Supplementary material for: Deterministic Evolutionary Trajectories Influence Primary Tumor Growth: TRACERx Renal
Source: Cell. 2018 Apr 19;173(3):595–610.e11. doi: 10.1016/j.cell.2018.03.043 (PMC5938372; doi:10.1016/j.cell.2018.03.043)
Supplement: Data S1. TRACERx Renal Cohort: VHL Summary, Related to Figure 1 and STAR Methods — VHL mutations as confirmed by Sanger sequencing, and VHL methylation results. [file mmc6.pdf]

VHL validation by Sanger sequencing

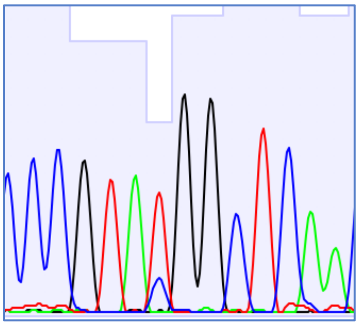

K079 VHL_3:10183793:T:C

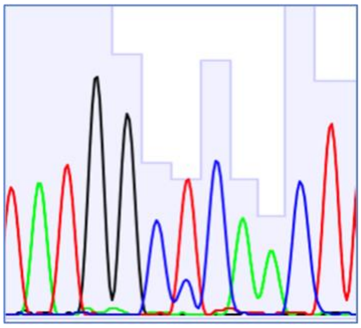

K119 VHL_3:10183797:T:C

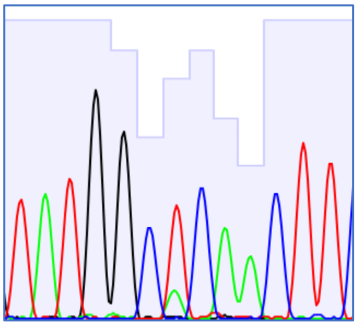

K165 VHL_3:10183797:T:A

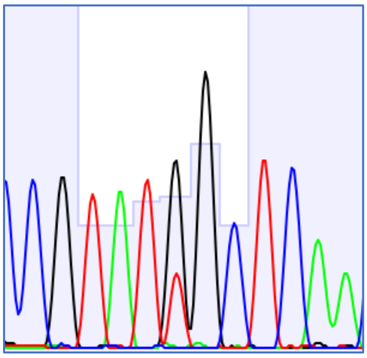

K180 VHL_3:10183794:G:T

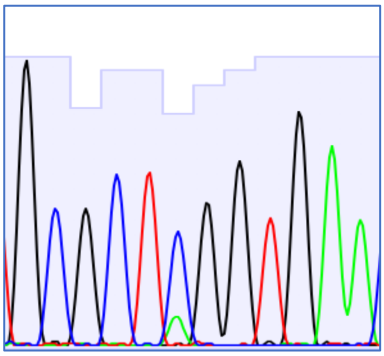

K191 VHL_3:10183725:C:A

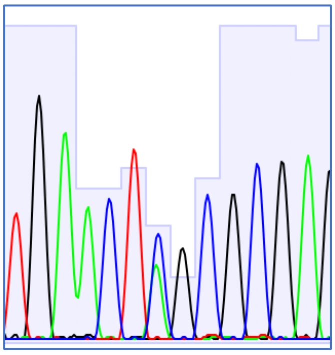

K229VHL_3:10183734:C:A

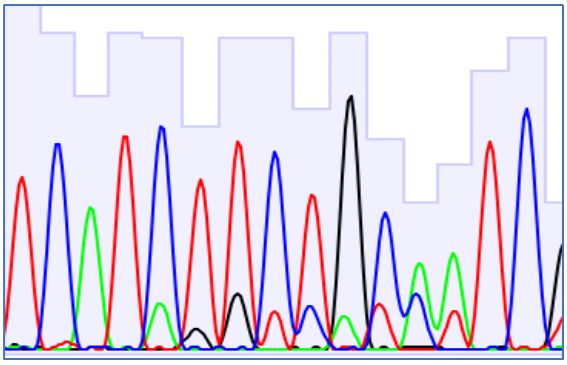

K220 VHL_3:10183748:10183748:-:AGGTC

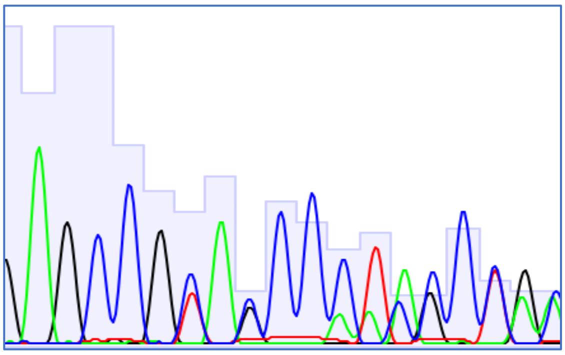

K182 VHL_3:10183846:10183846:-:GGCCG

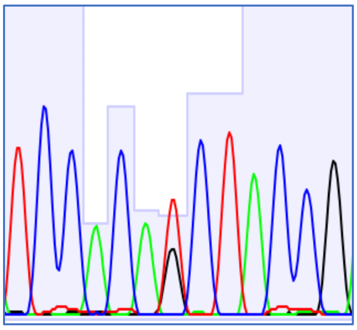

K237 VHL_3:10183863:G:T

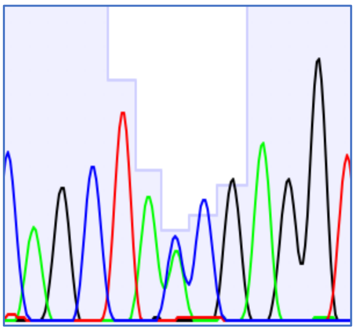

K249 VHL_3:10183867:C:A

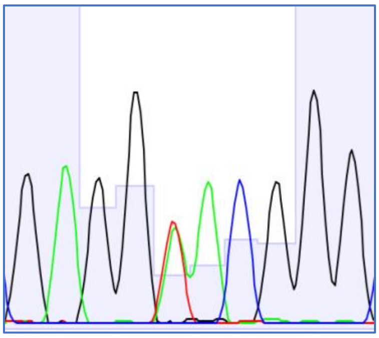

K272 VHL_3:10183873:T:A

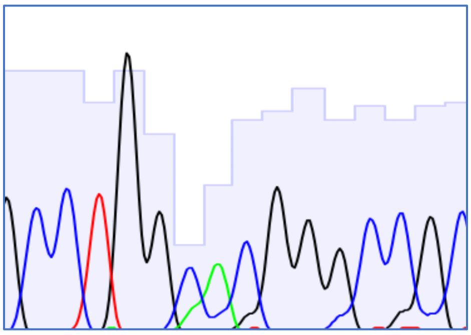

K250 VHL_3:10183843:10183843:C:-

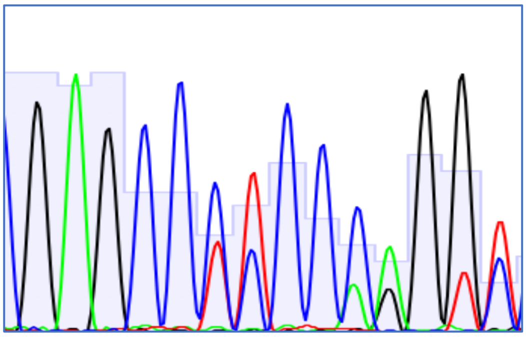

K334_L VHL_3:10183742:10183742:C:-

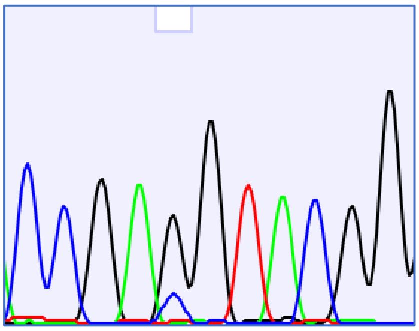

K276 VHL_3:10183873:T:A

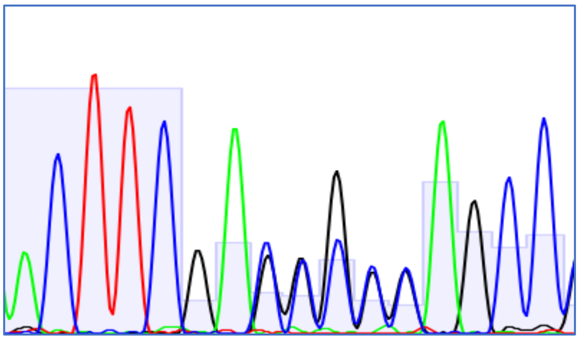

K376 VHL_3:10183804:10183809:CGACG:-

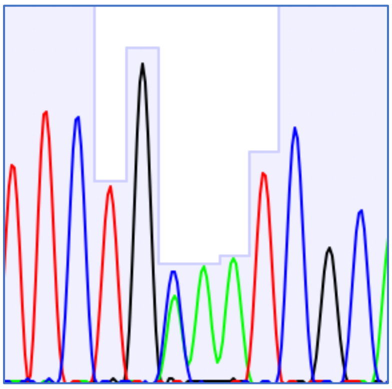

K448 VHL_3:10183762:C:A

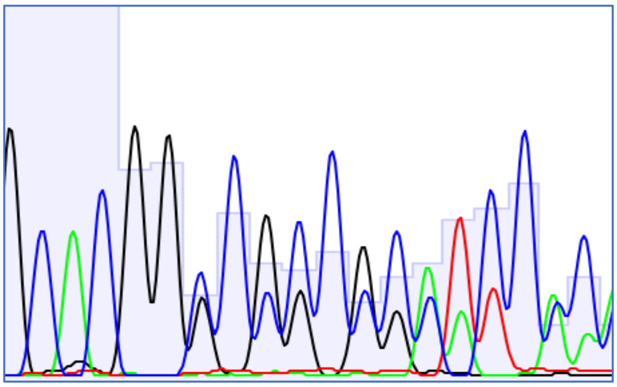

K097_R VHL_3:10183846:10183846:G:-

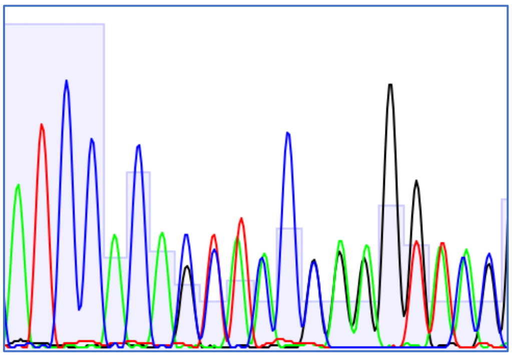

K104 VHL_3:10183863:10183863:G:-

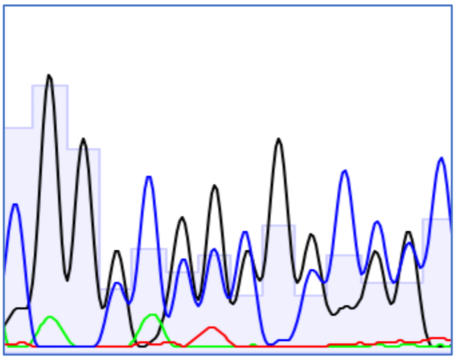

K130 VHL_3:10183712:10183712:C:-

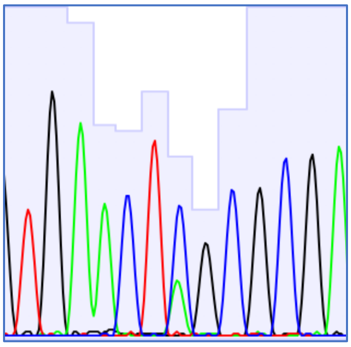
K138 VHL_3:10183734:C:A

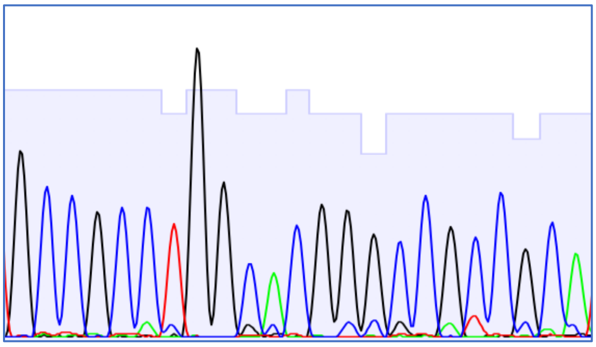
K143 VHL_3:10183837:10183841:GCCTG:-

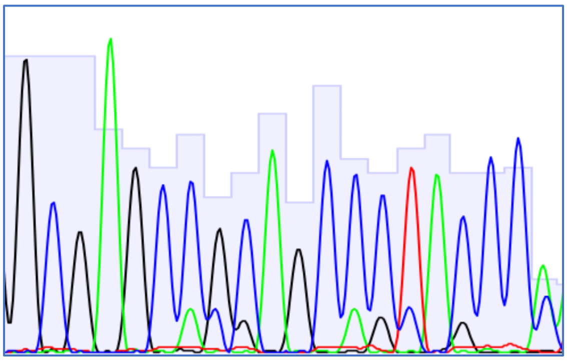
K150 VHL_3:10183809:10183809:-:CGAGC

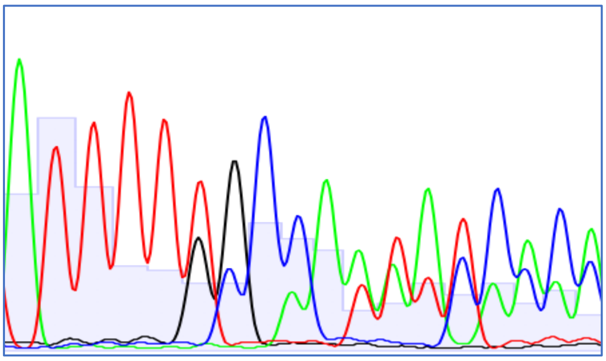
K065 VHL_3:10188297:10188297:T:-

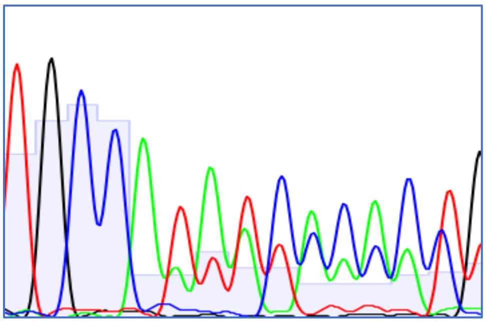
K107 VHL_3:10188305:10188305:A:-

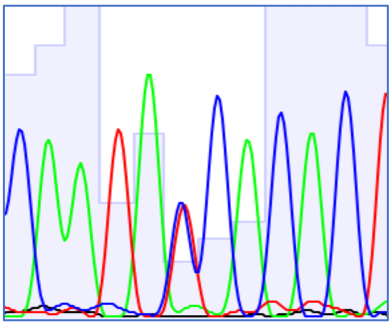
K114 VHL_3:10188309:T:C

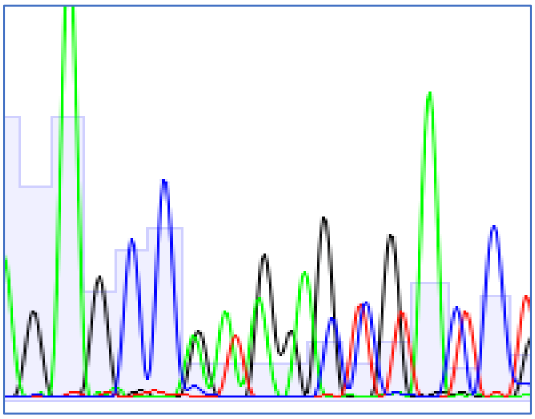

K124 VHL_3:10188203:10188206:CTTT:-

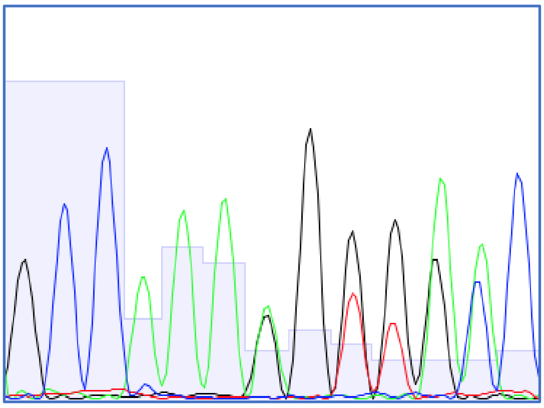

K153 VHL_3:10188203:10188203:-:T

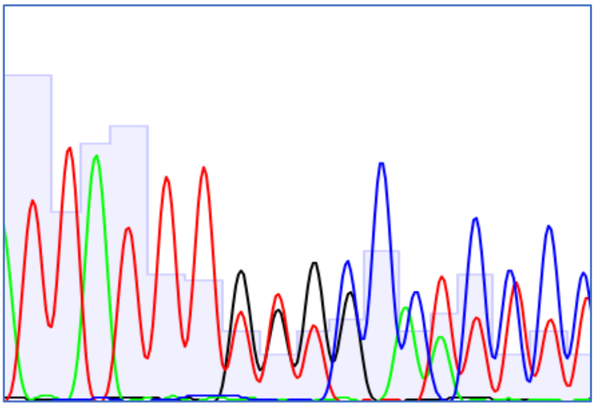

K176 VHL_3:10188252:10188252:A:-

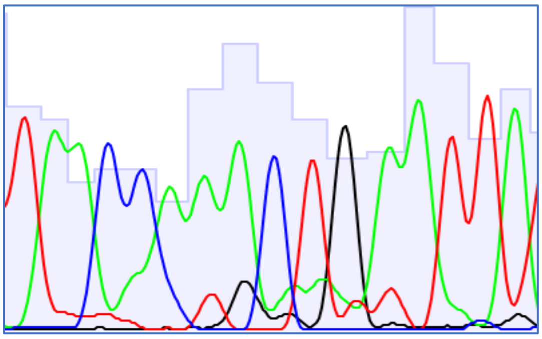

K226 VHL_3:10188251:10188253:CAA:-

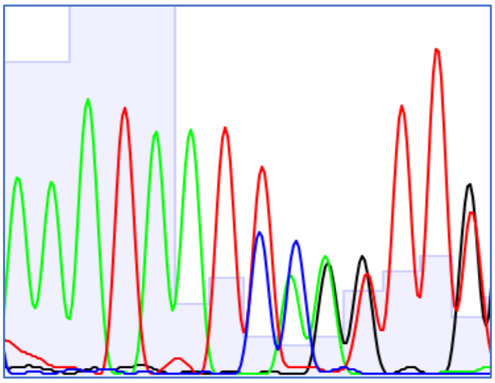

K450 VHL_3:10188258:10188258:A:-

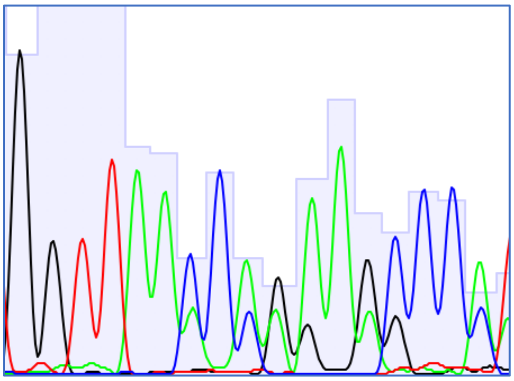

K023 VHL_3:10188245:10188245:-:T

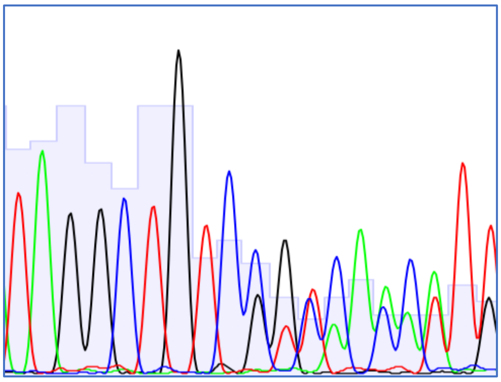

K146 VHL_3:10188287:10188287:G:-

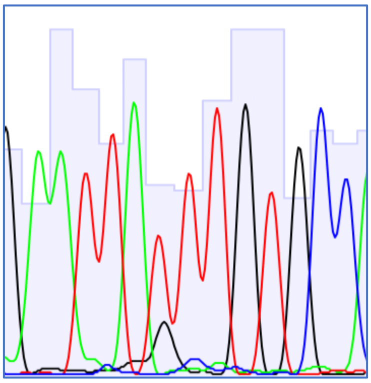

K158 VHL_3:10188263:T:G

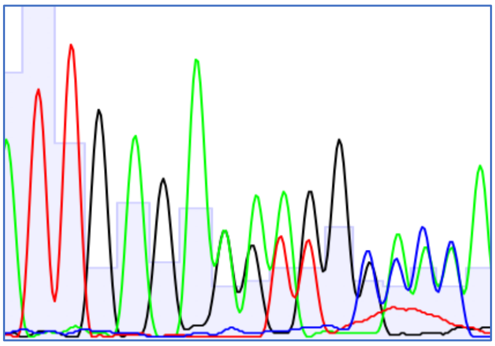

K162 VHL_3:10188273:10188273:-:T

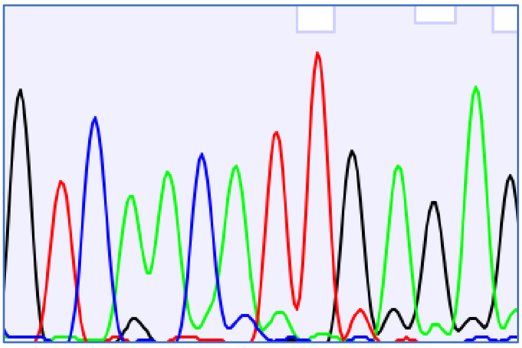

K328 VHL_3:10188283:10188283:-:C

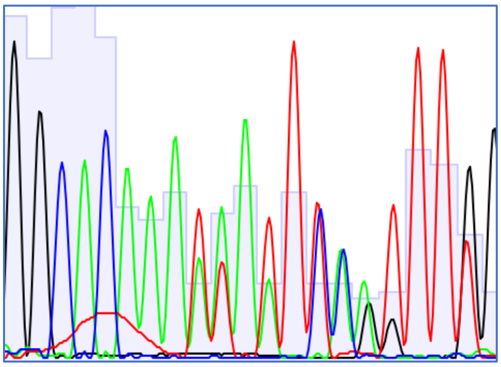

K263 VHL_3:10188262:10188262:-:T

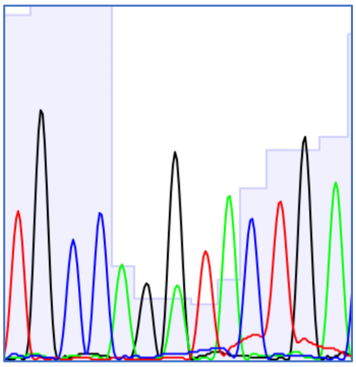

K265 VHL_3:10188321:G:A

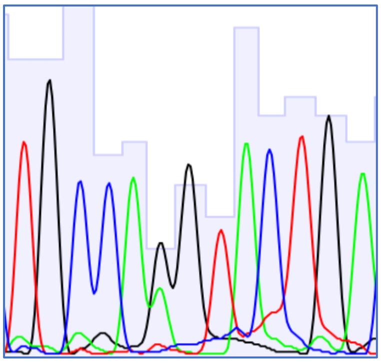

K306 VHL_3:10188320:G:A

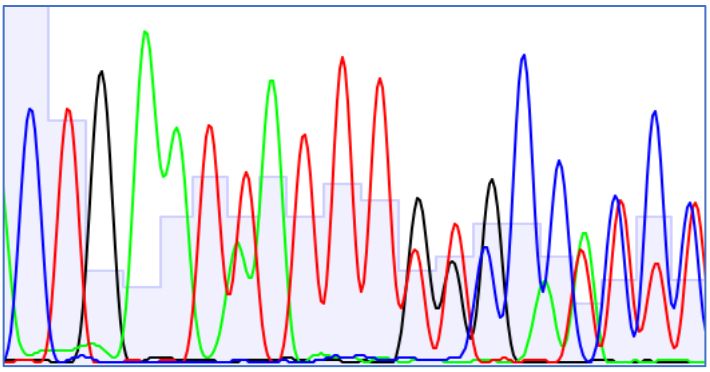

K323 VHL_3:10188266:10188266:G:-

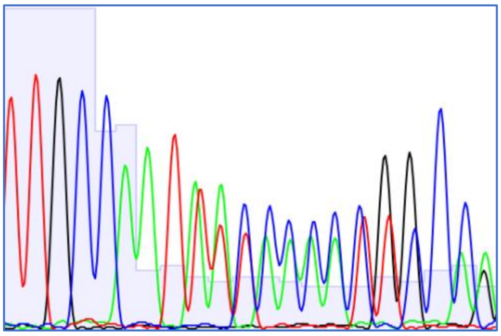

K386 VHL_3:10188306:10188306:-:T

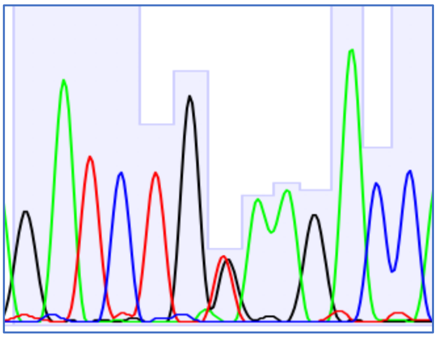

K059 VHL_3:10191572:G:T

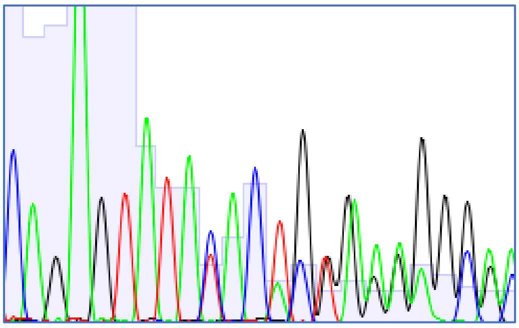

K123 VHL_3:10191472:10191472:-:TA

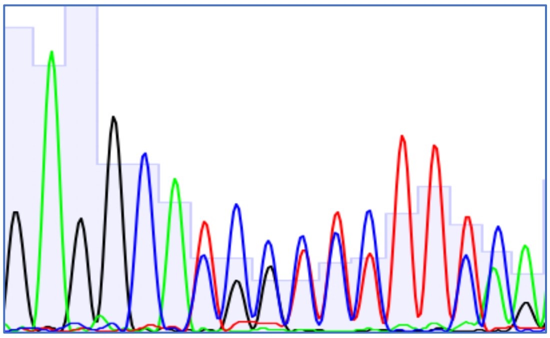

K136 VHL_3:10191490:10191490:A:-

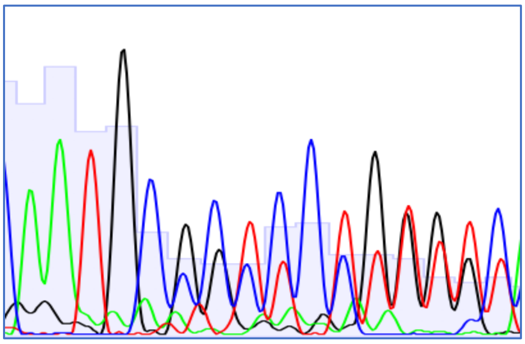

K234 VHL_3:10191620:10191620:-:G

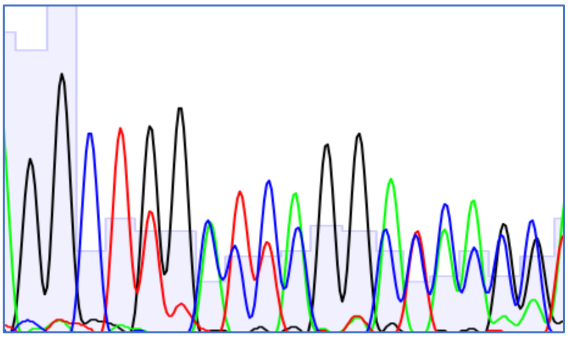

K243 VHL_3:10191513:10191518:TAGTC:-

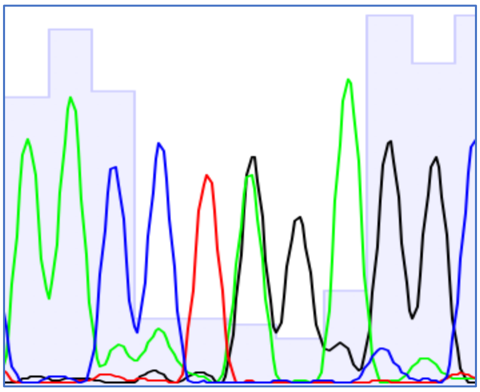

K252 VHL_3:10191497:C:T

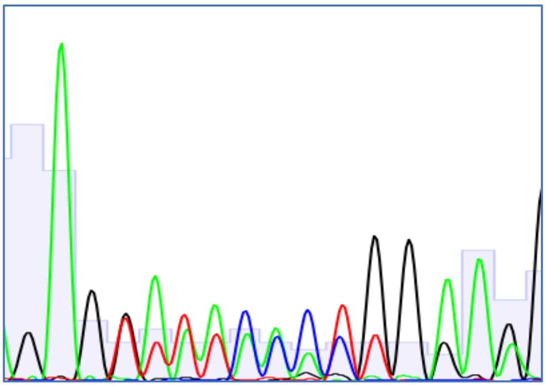

K352 VHL_3:10191476:10191476:-:C

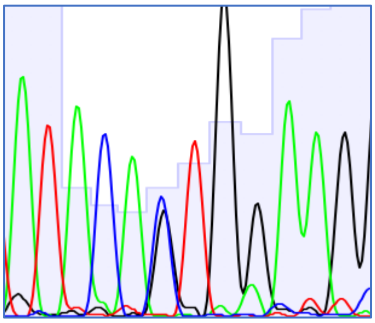

K360 VHL_3:10191470:G:C

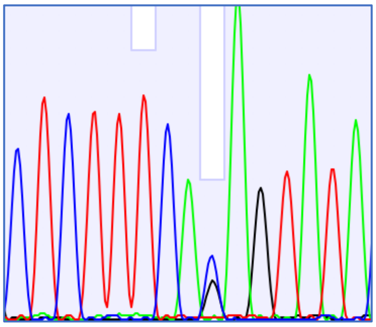

K520 VHL_3:10191479:C:G

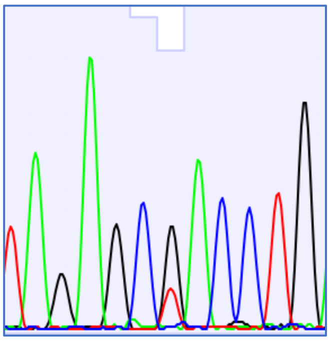

K523 VHL_3:10191555:C:A

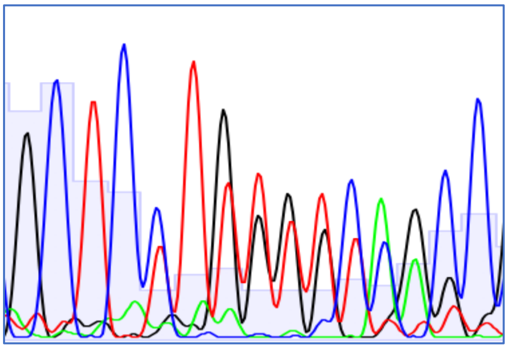

K027 VHL_3:10191614:10191614:-:A

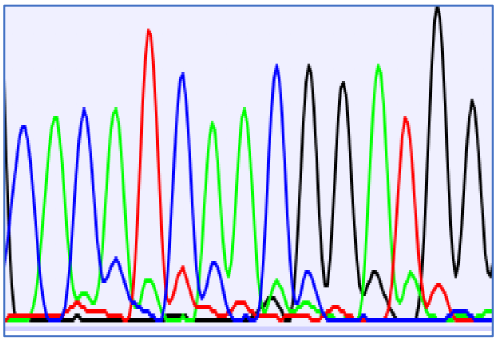

K032 VHL_3:10191627:10191627:-:A

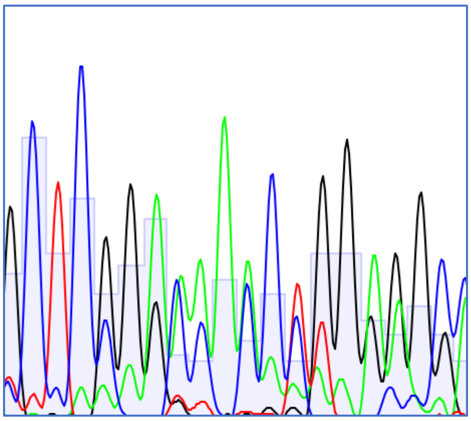

K099 VHL_3:10191507:10191507:G:-

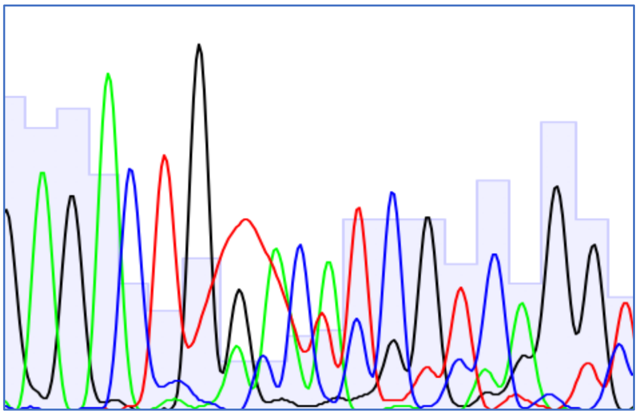

K128 VHL_3:10191541:10191541:G:-

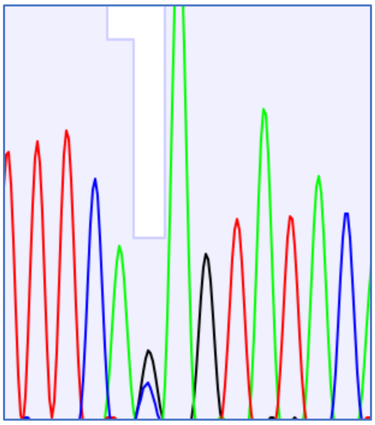

K167 VHL_3:10191479:C:G

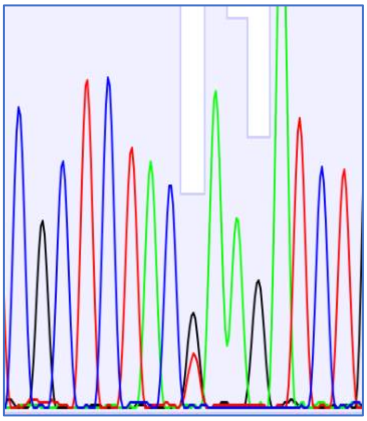

K207 VHL_3:10191563:G:T

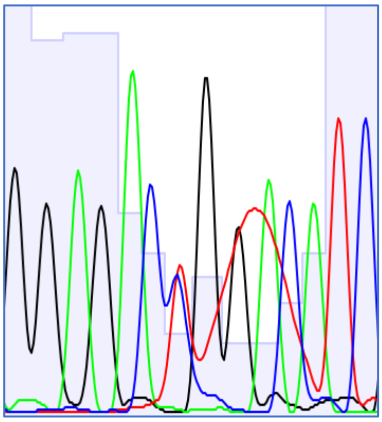

K208 VHL_3:10191540:T:C

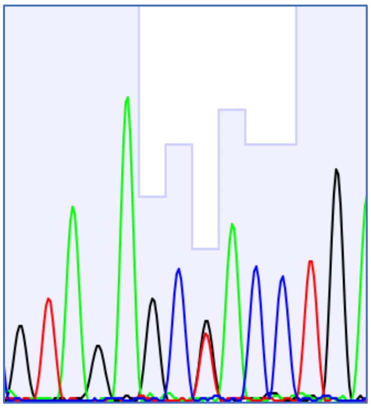

K242 VHL_3:10191555:C:A

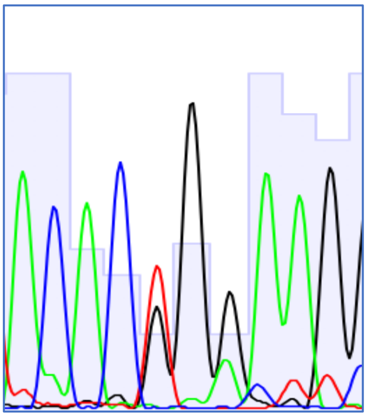

K245 VHL_3:10191469:A:C

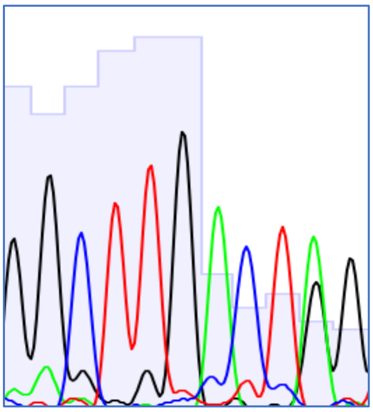

K265 VHL_3:10191513:T:C

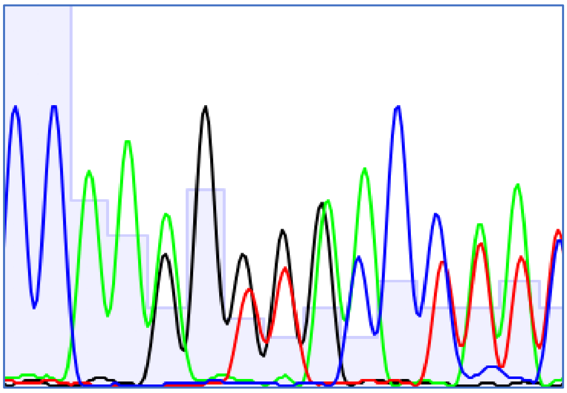

K256 VHL_3:10188204:10188204:T:-

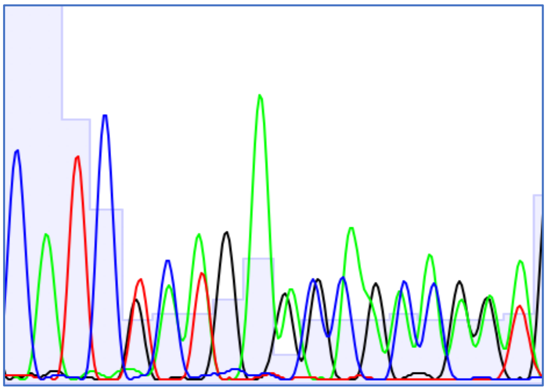

K334 VHL_3:10188215:10188217:AGA:-

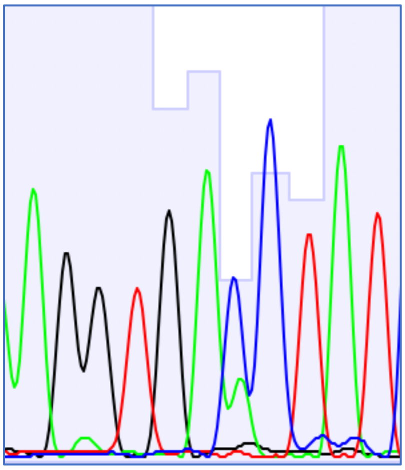

K352 VHL_3:10188198:G:T

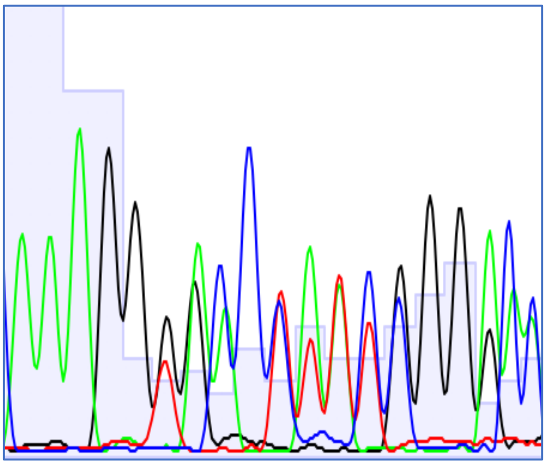

K375 VHL_3:10188201:10188201:A:-

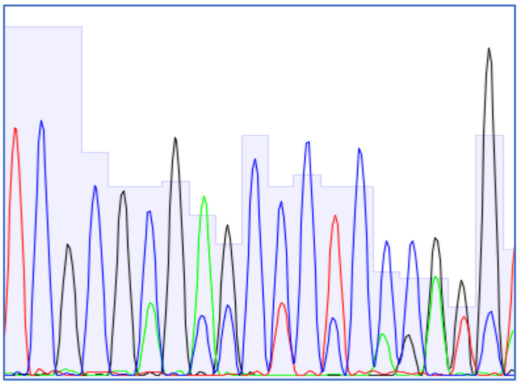

K039 VHL_3:10183734:10183735:CG:-

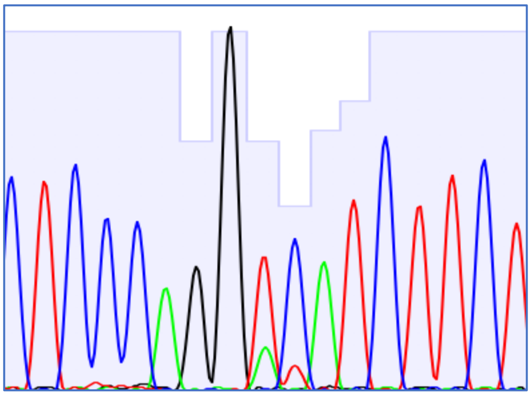

K156 VHL_3:10183752:10183753:TC:AT

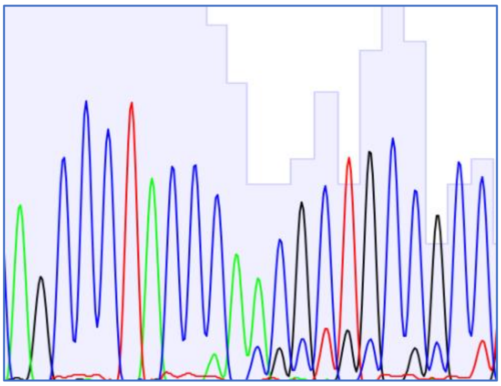

K326 VHL_3:10183825:10183825:C:-

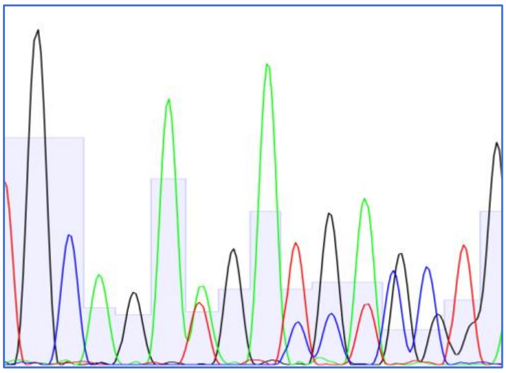

K184 VHL_3:10183755:10183757:TCT:-

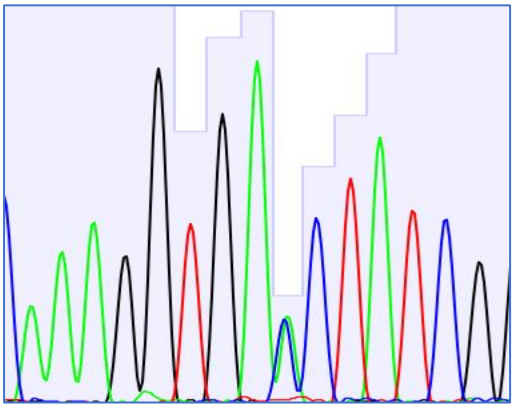
K216 VHL_3:10188198:G:T

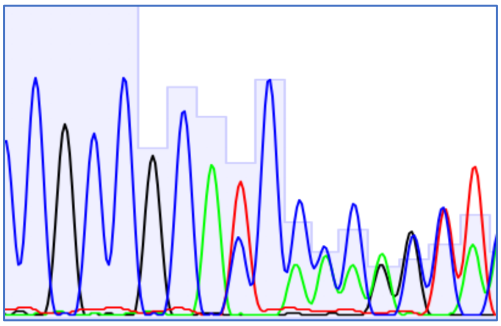
K217 VHL_3:10183857:10183857:T:-

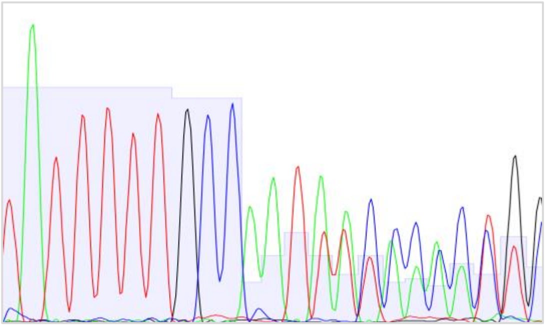
K386 VHL_3:10188306:10188306:-:T

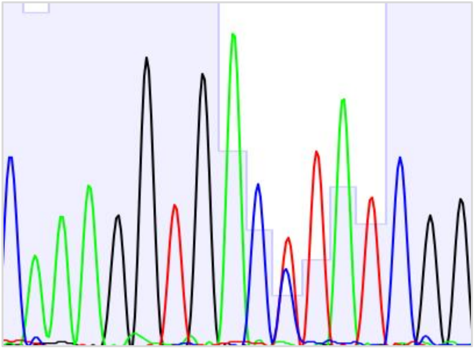
K118 VHL_3:10188197:G:A

VHL methylation Profiles

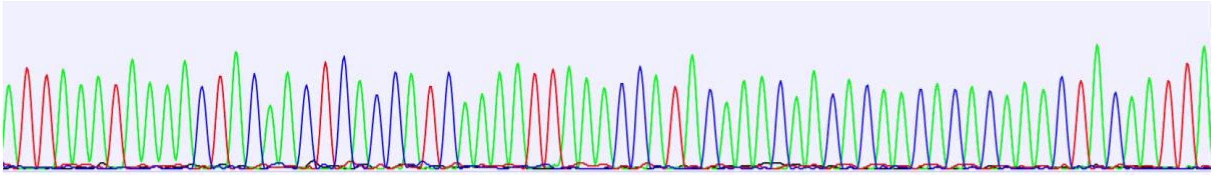

K064_N

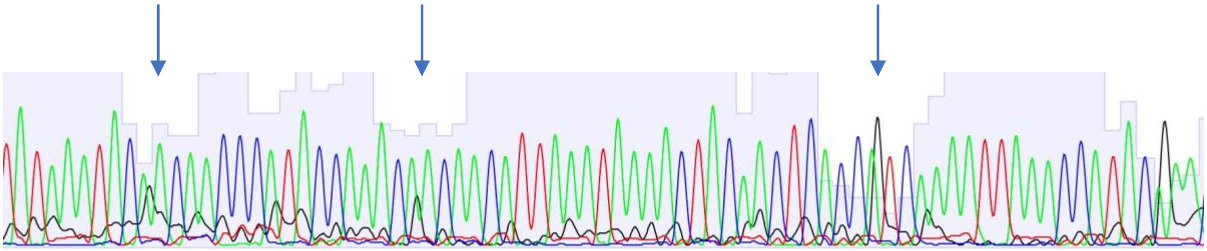

K064_R1

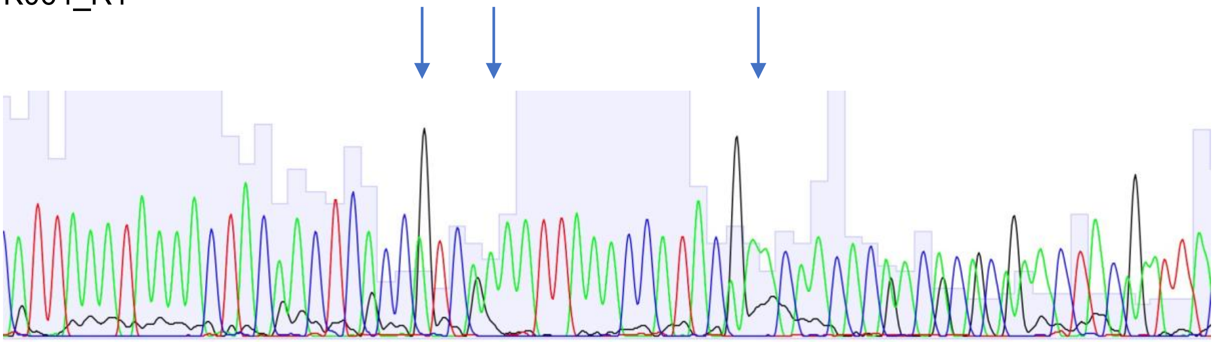

K064_R2

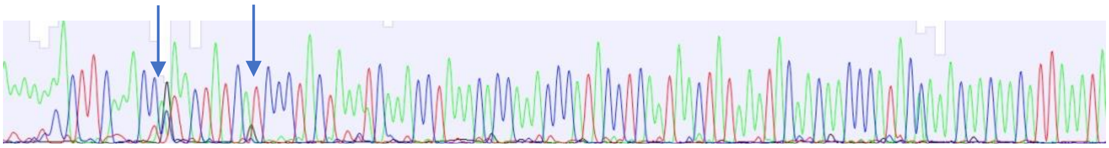

K222_R2

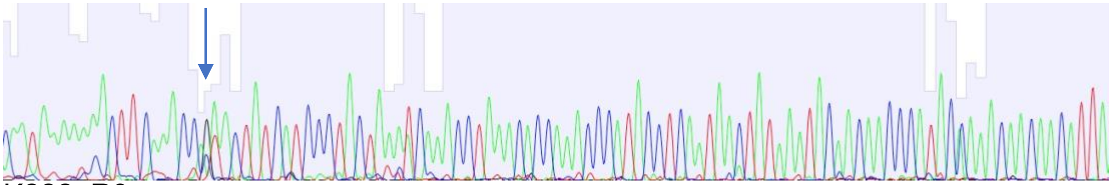

K222_R3

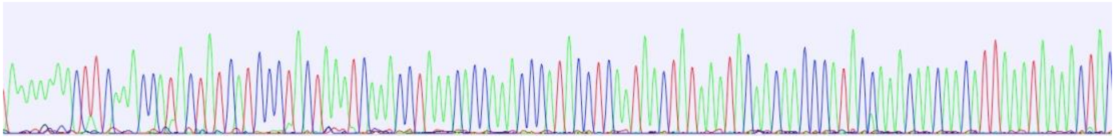

K222_N

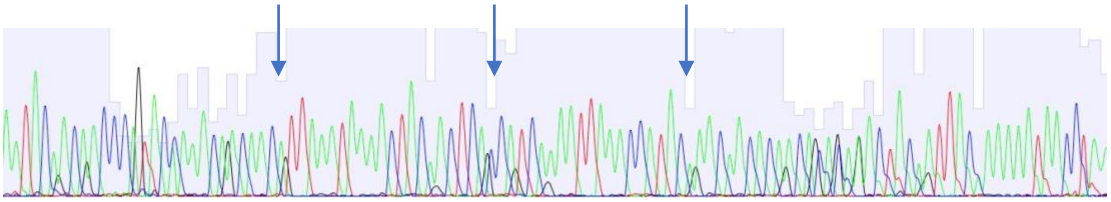

K233_R3

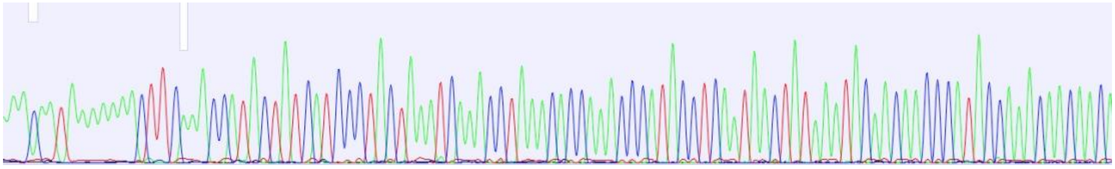

K233_N

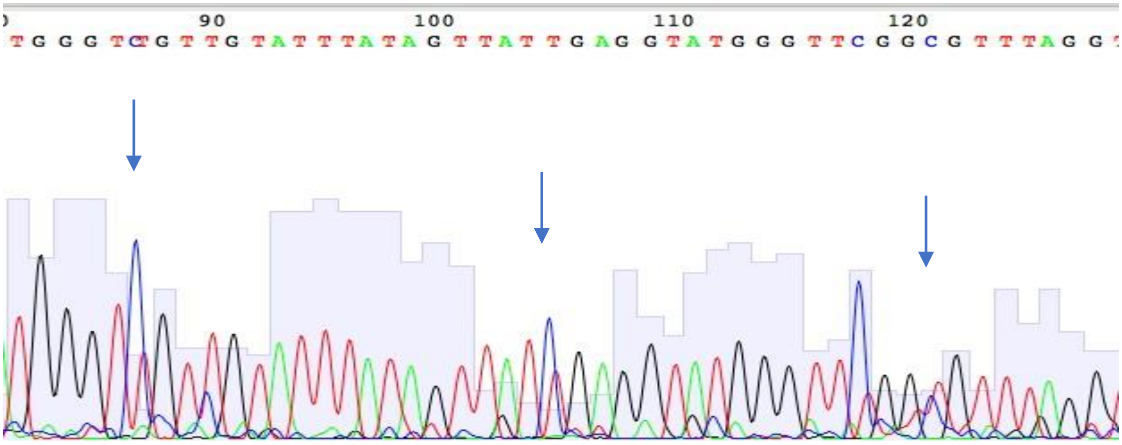

K239_R3

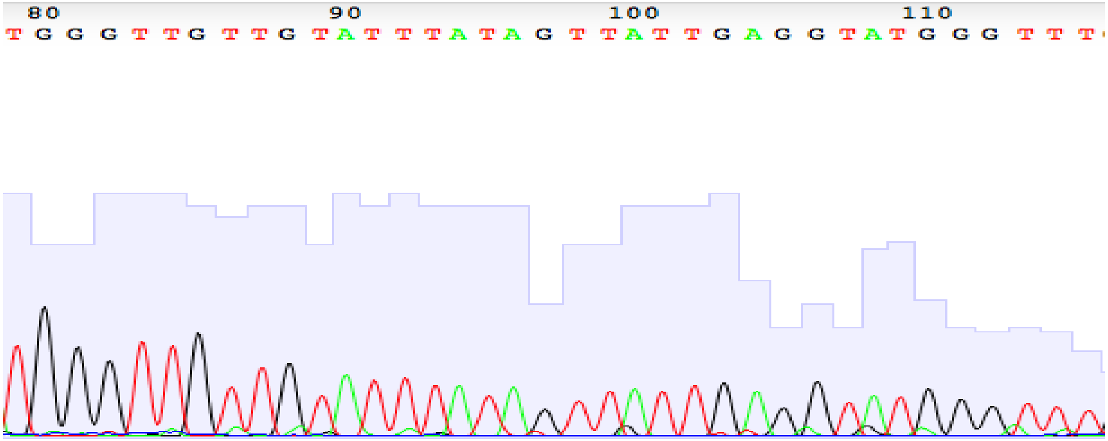

K239_N

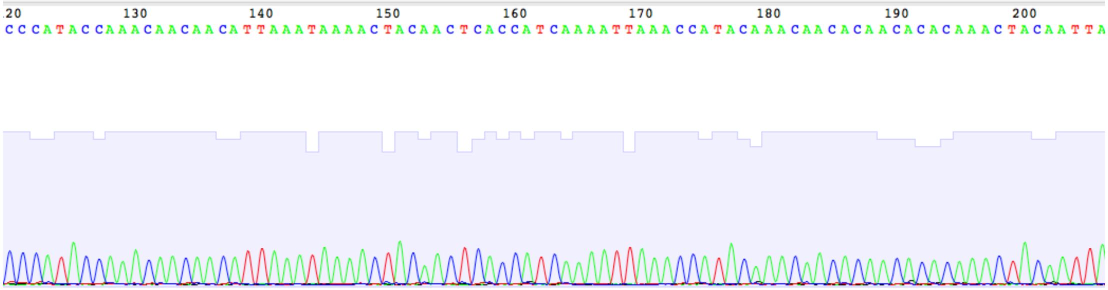

K271_R1

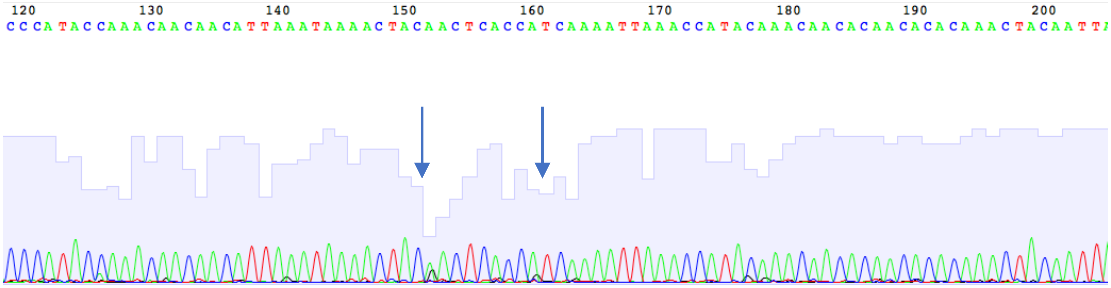

K271_R2

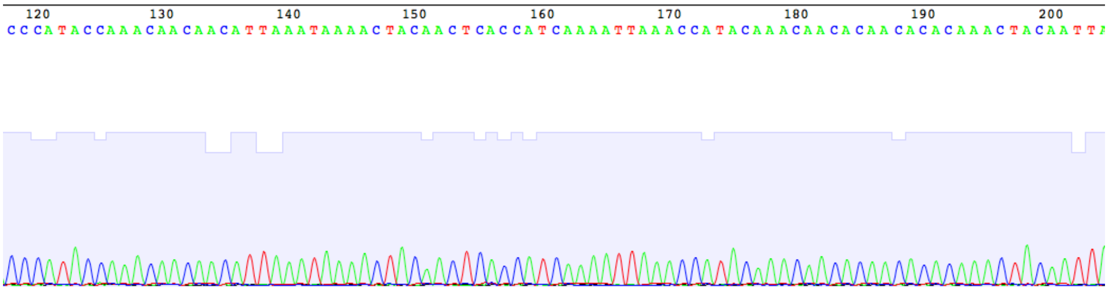

K271_N

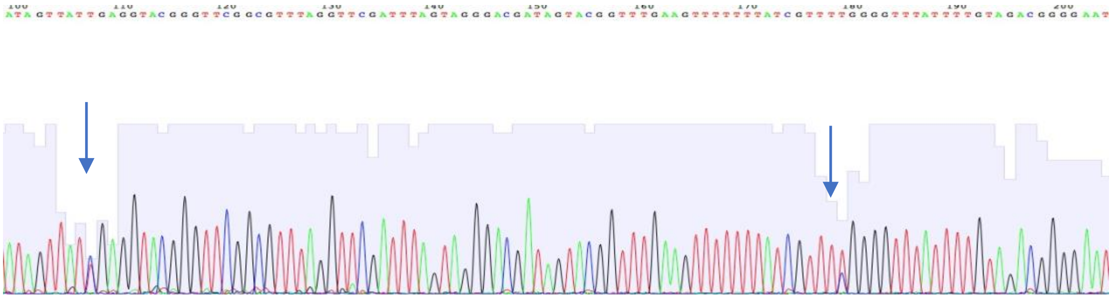

K354_R4

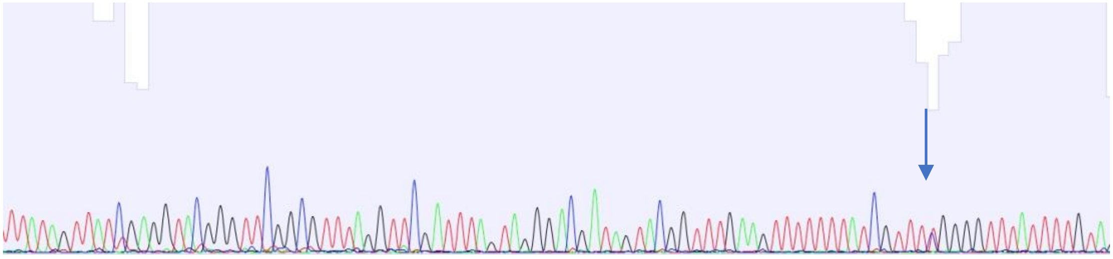

K354_R3

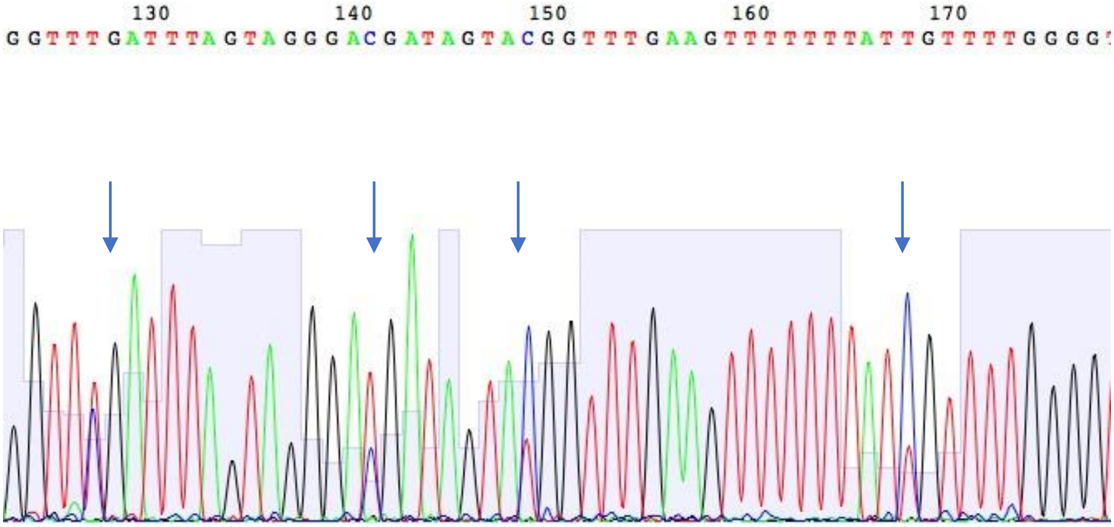

K379_R22

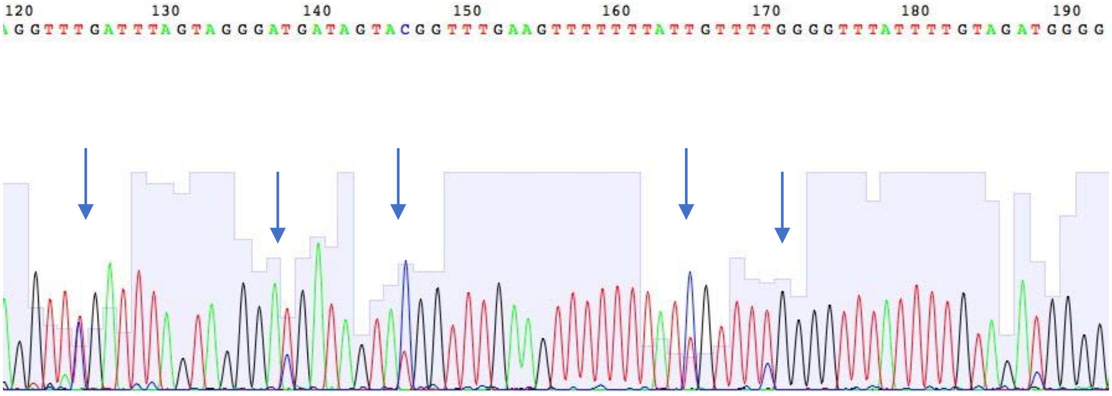

K379_R23

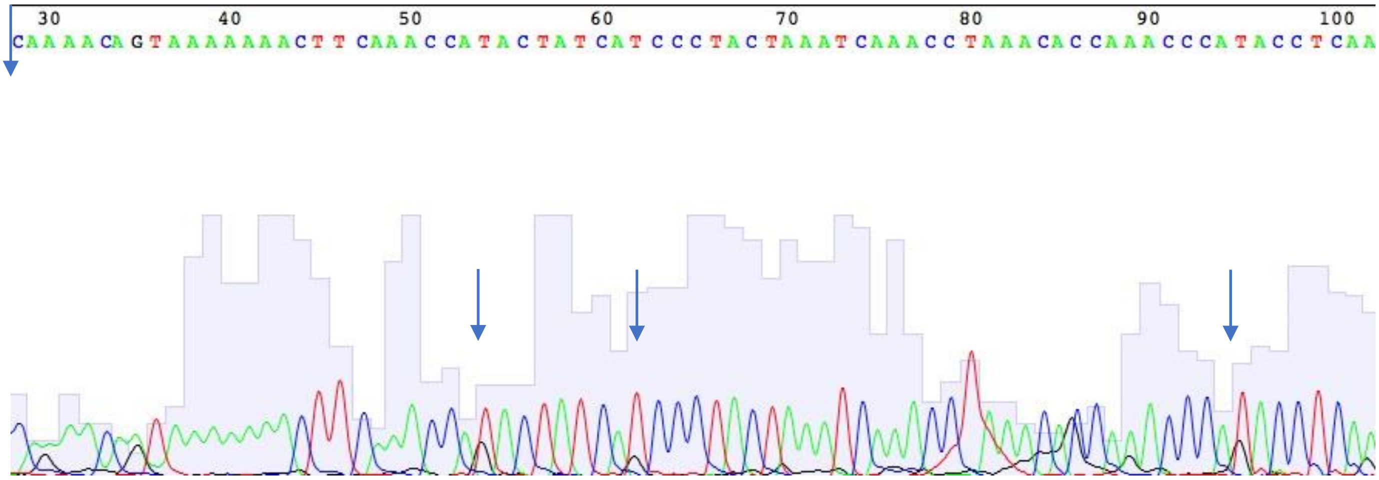

K163_Tumour

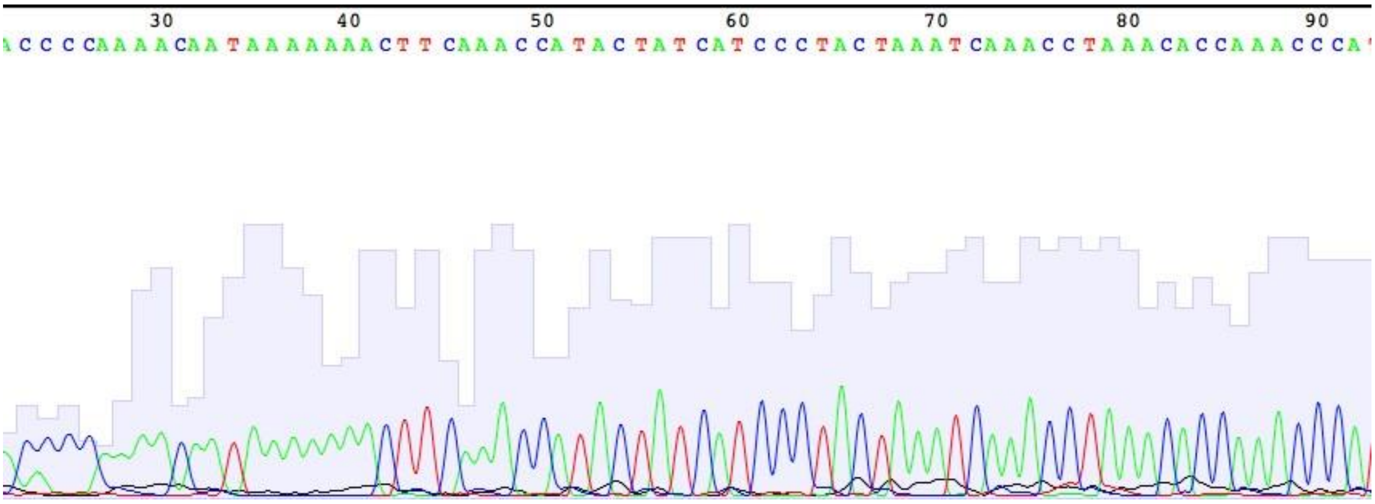

K163_N

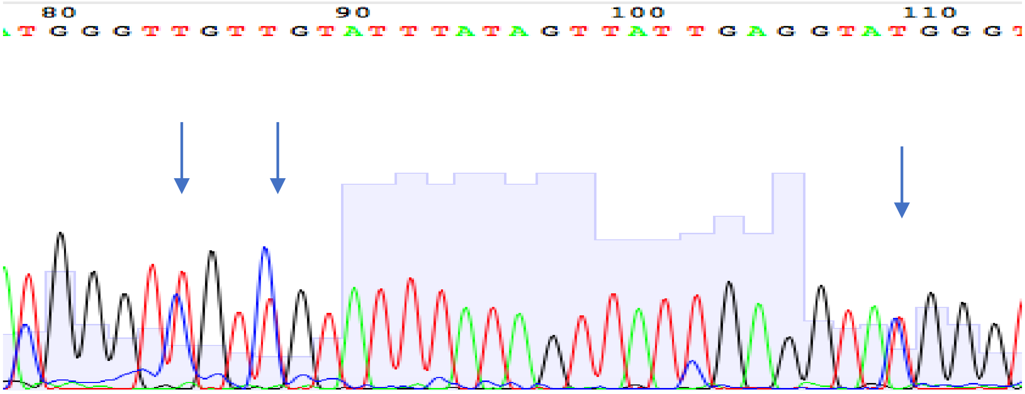

K029_R3

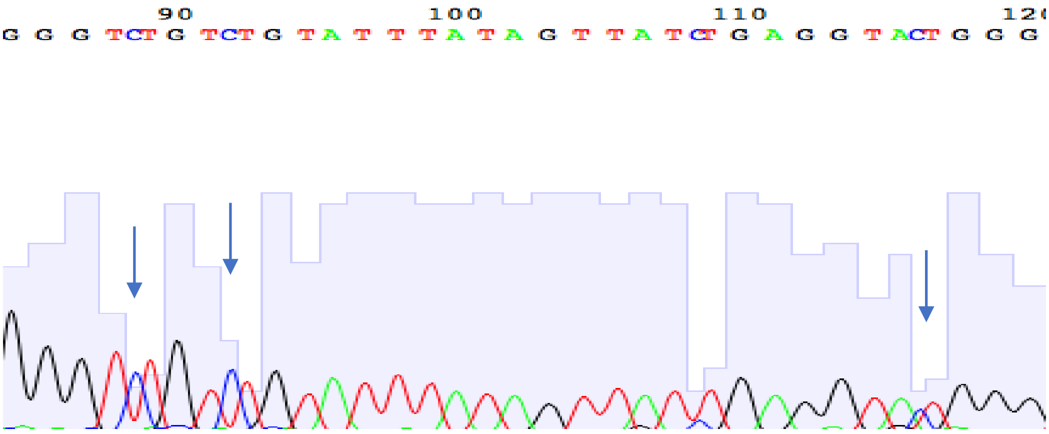

K029_R9

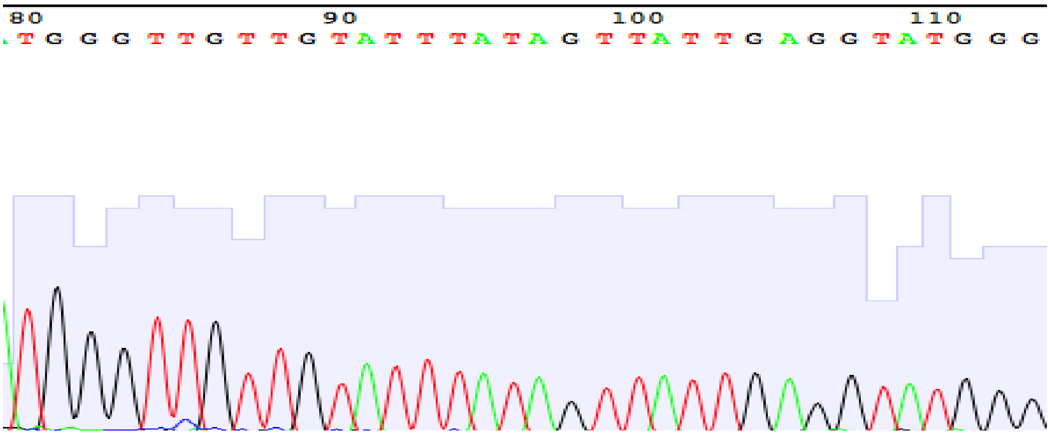

K029_N

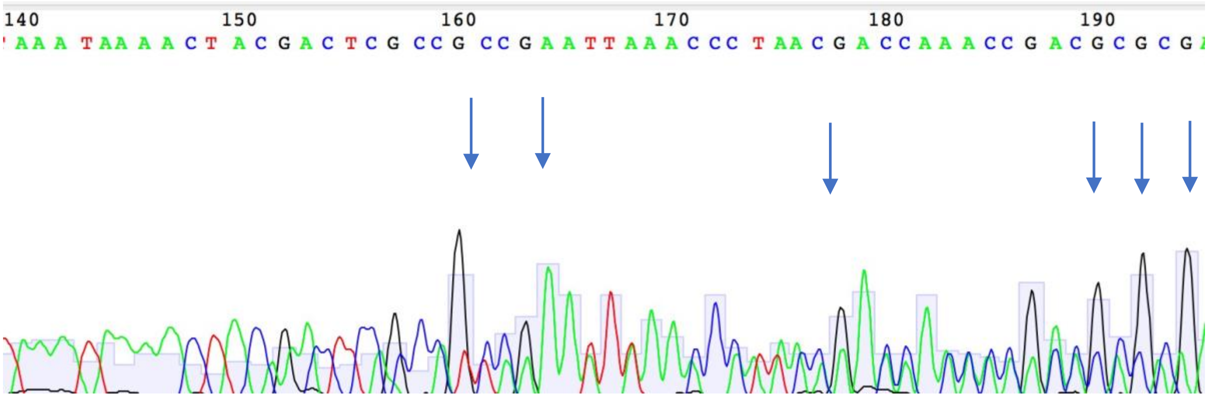

K070_R4

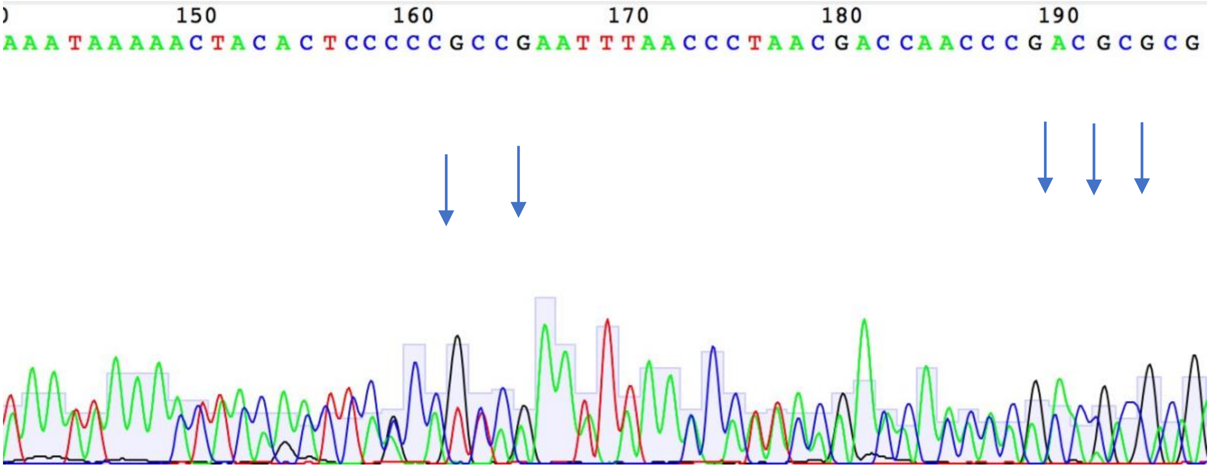

K070_R9

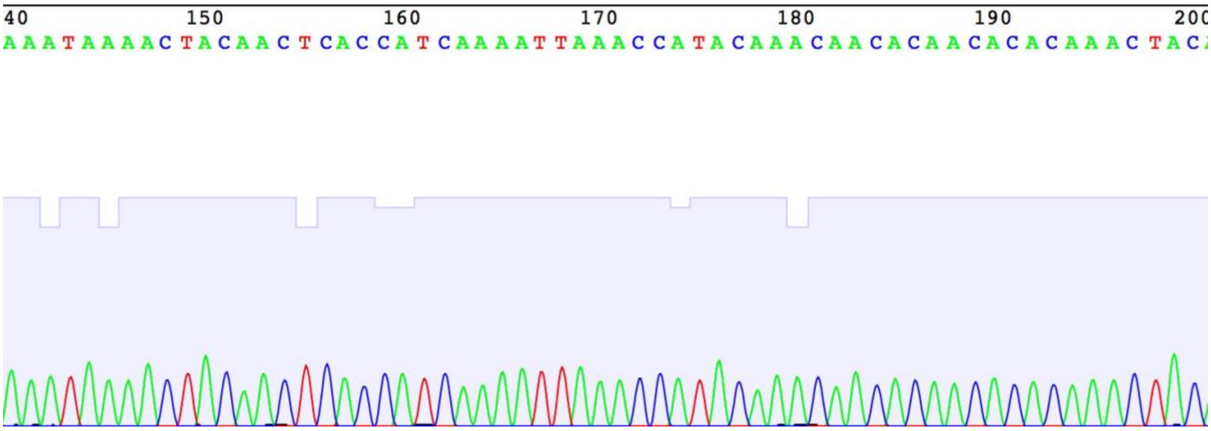

K070_N

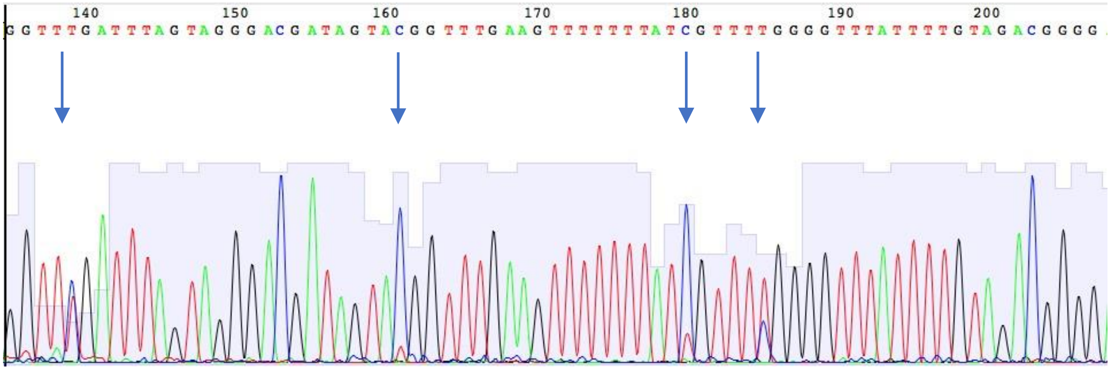

K098_R1

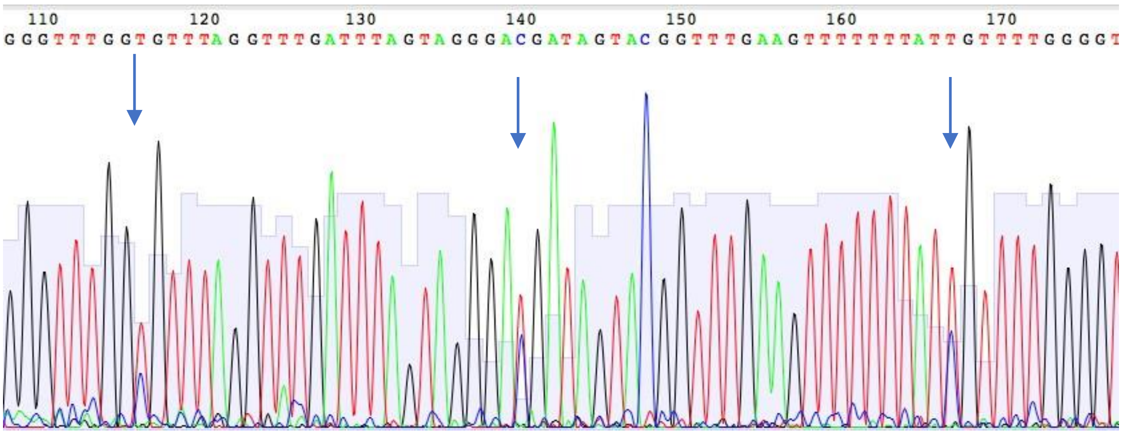

K108_R3

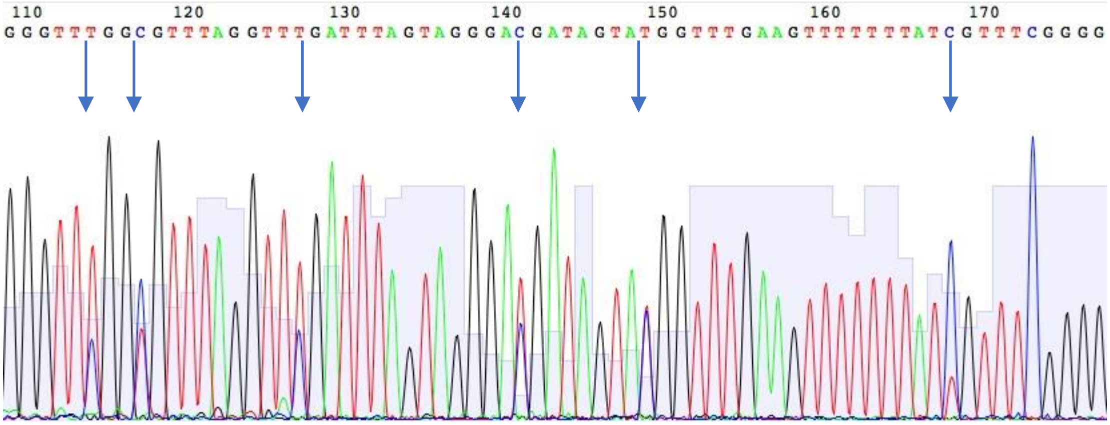

K108_R6

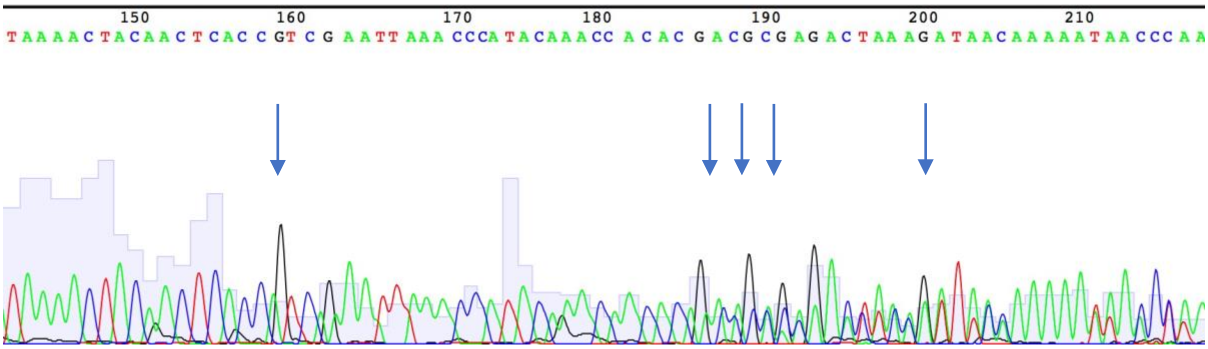

K135_Tumour

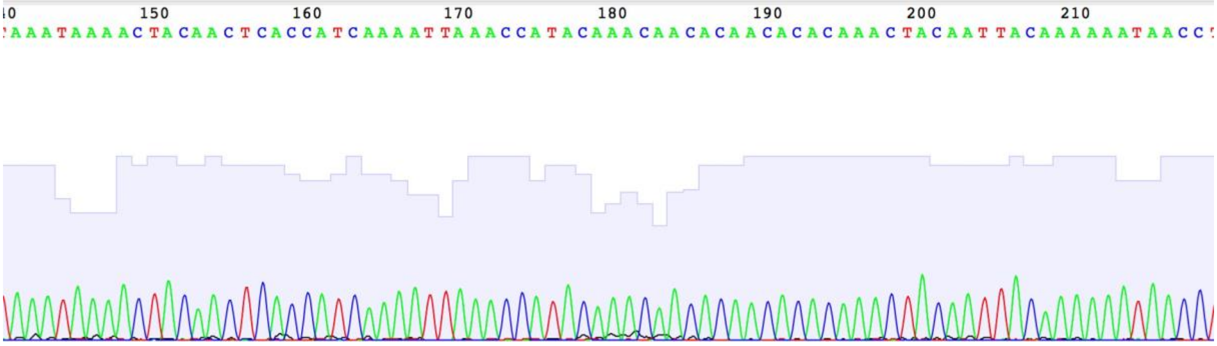

K135_Normal

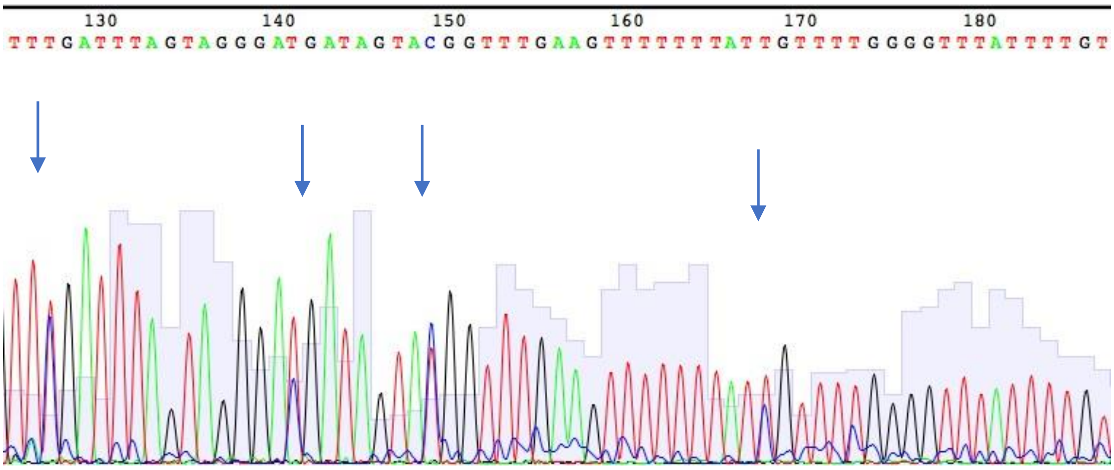

K177_R2

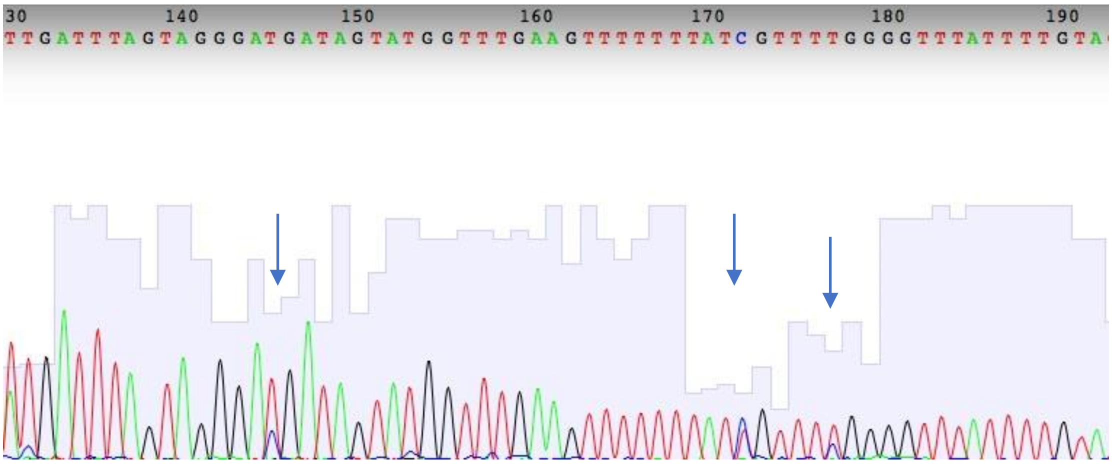

K177_R4

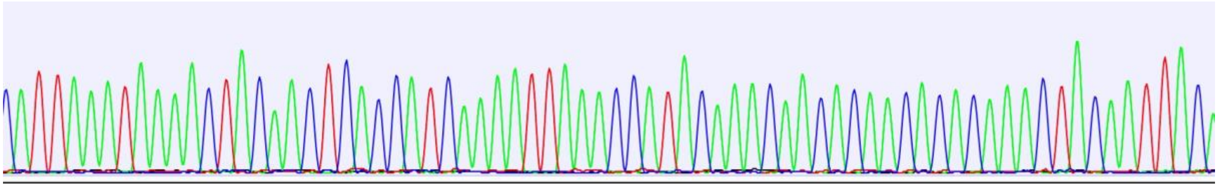
K304_N

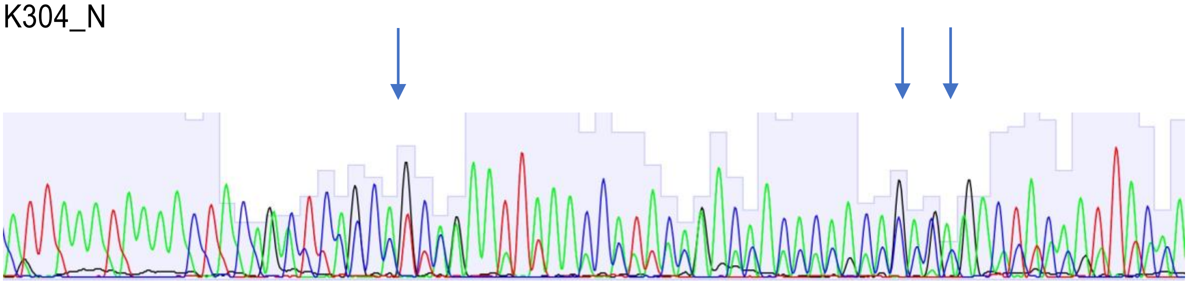
K304_R3

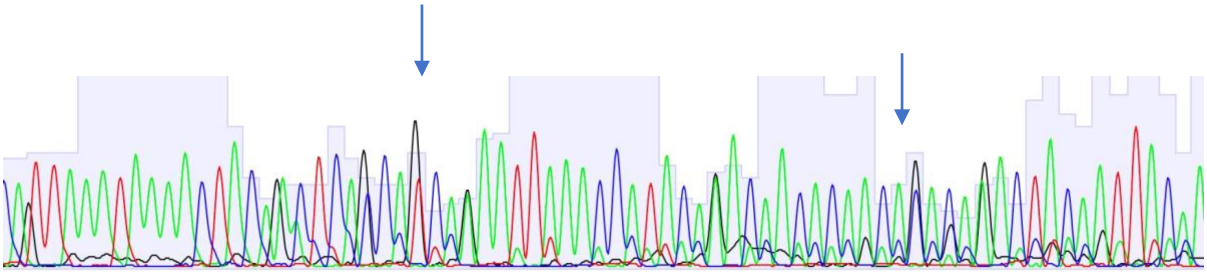
K304_R4

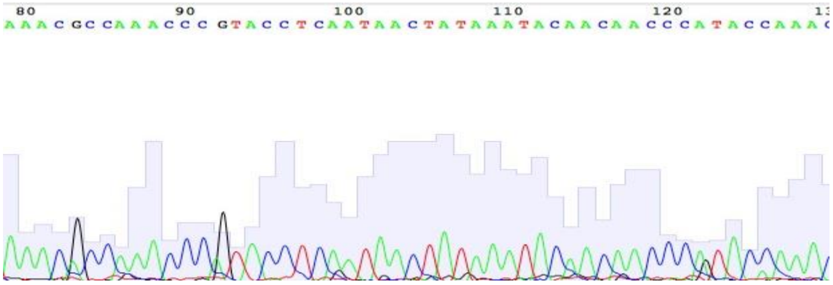
K308_R2

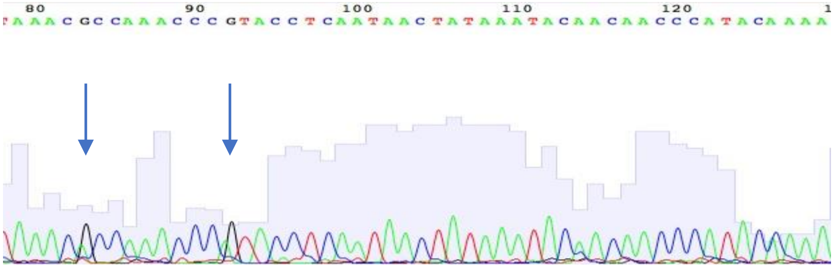
K308_R3

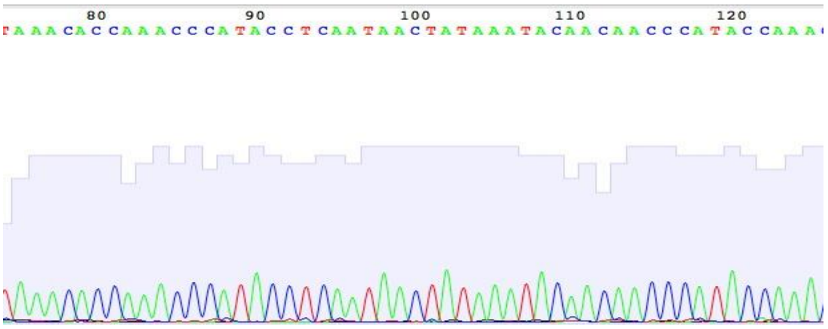
K308_N

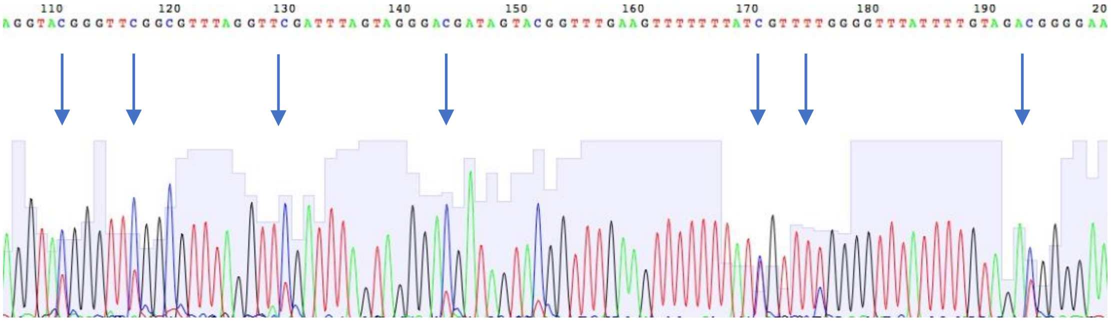
K463_R8

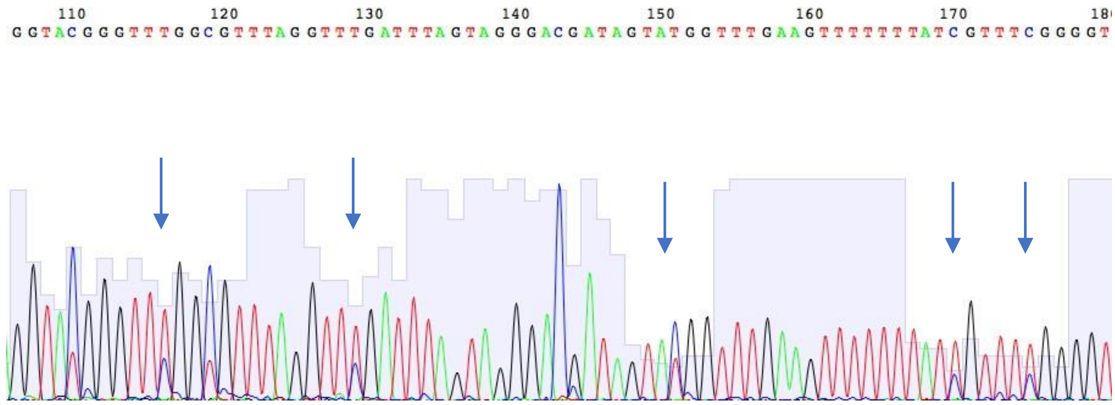

K463_R17

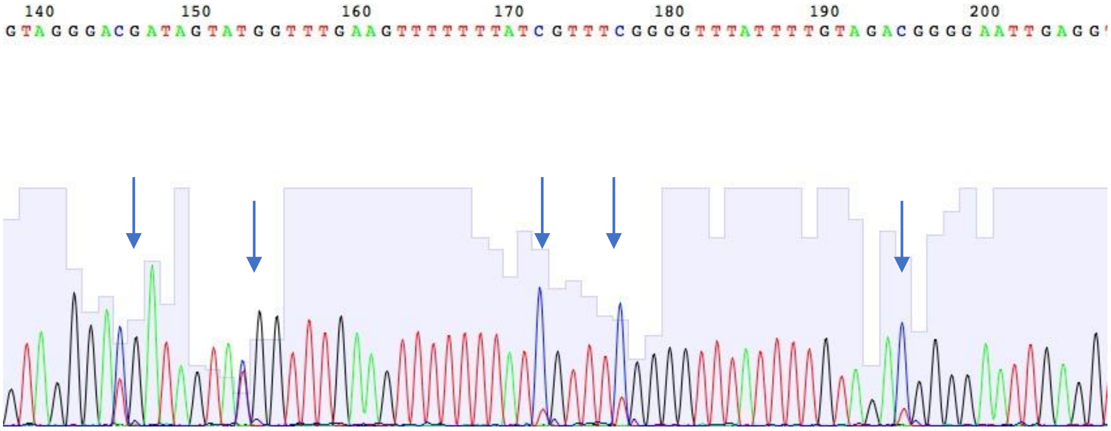

K463_R3

Sanger sequence trace of the VHL INDEL identified in the right kidney tumour of patient K097 and VHL methylation in the left kidney tumour.

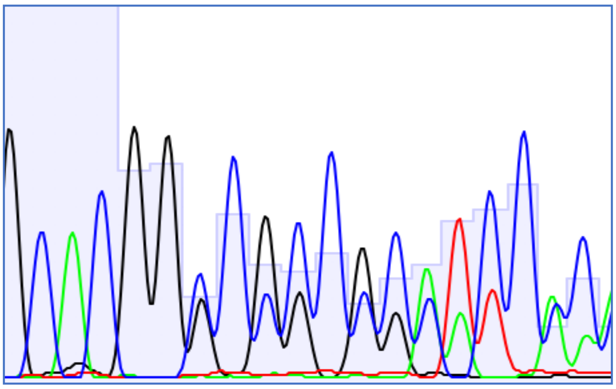

K097_Right Kidney VHL_3:10183846:10183846:G:-

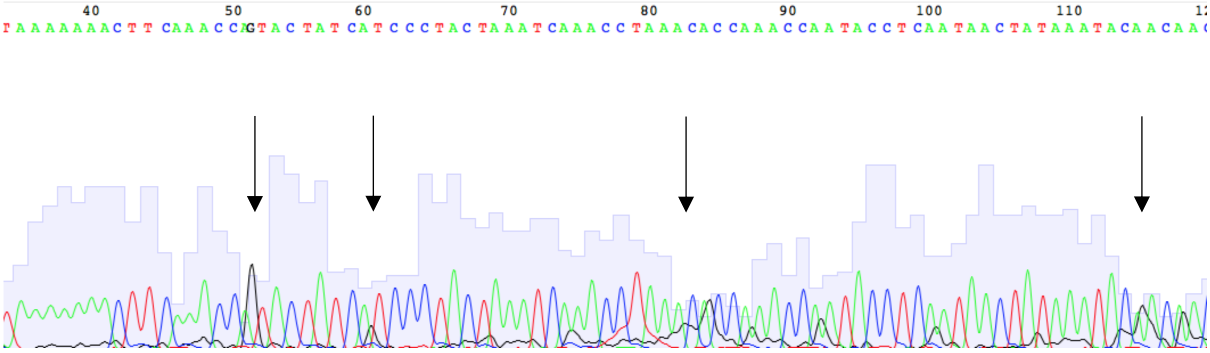

K097_Tumour_methylation – Left Kidney

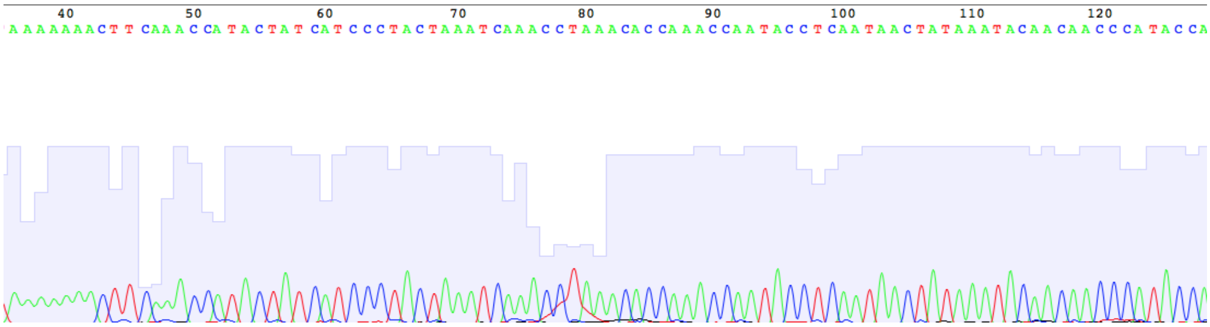

RK97_Normal Kidney Tissue

VHL mutations in left K150 tumours (historical)

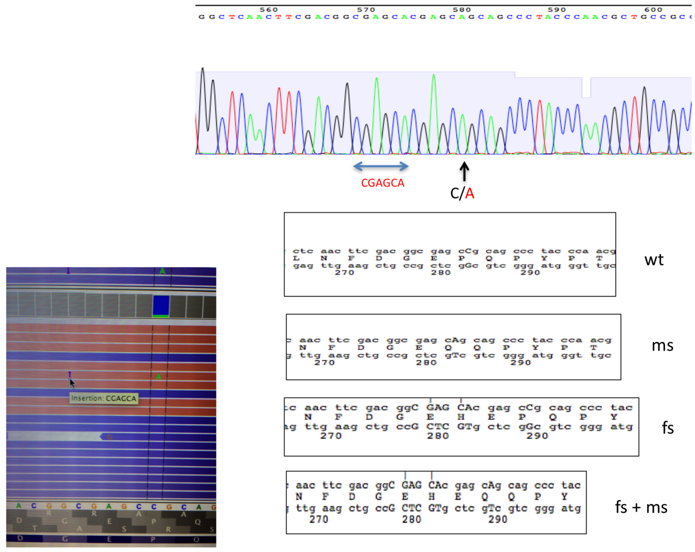

Oligonucleotide sequences for VHL exon amplification and methylation specific PCR

Oligonucleotide name	Oligonucleotide Sequence 5’-3’
VHL_X1_F	gtggaaatacagtaacgagttgg
VHL_X1_R	caggcaaaaattgagaactgg
VHL_X2_F	aacctttgcttgtcccgata
VHL_X2_R	caggcaaaaattgagaactgg
VHL_X3a_F	ctgccactgaggatttggtt
VHL_X3a_R	ccatcaaaagctgagatgaaa
VHL_methylation_1F	gagtttttttaggttatttttgtaat
VHL_methylation_1R,	tcaccctaaatatatatcctacctcaaaa
VHL_methylation_2F	cccctctaaaatttaatattttt
VHL_methylation_2R	ggttaaggttgtagtgagttaagtt
