# Supplementary material for: Deterministic Evolutionary Trajectories Influence Primary Tumor Growth: TRACERx Renal
Source: Cell. 2018 Apr 19;173(3):595–610.e11. doi: 10.1016/j.cell.2018.03.043 (PMC5938372; doi:10.1016/j.cell.2018.03.043)
Supplement: Data S2. Mutation Heatmap and Driver Trees for a Subset of TRACERx Renal Cohort, Related to Figures 1 and 2 — Mutation heatmap and driver trees, for individual cases as referenced in the Results section. [file mmc7.pdf]

Presence and absence of mutational events for each tumour region are presented in heatmap (light gray means absence), with clonal events are presented as colored box and subclonal events are presented as colored triangles. Driver phylogenetic trees were build based on the presence or absence of mutational events (Please see Star Methods).

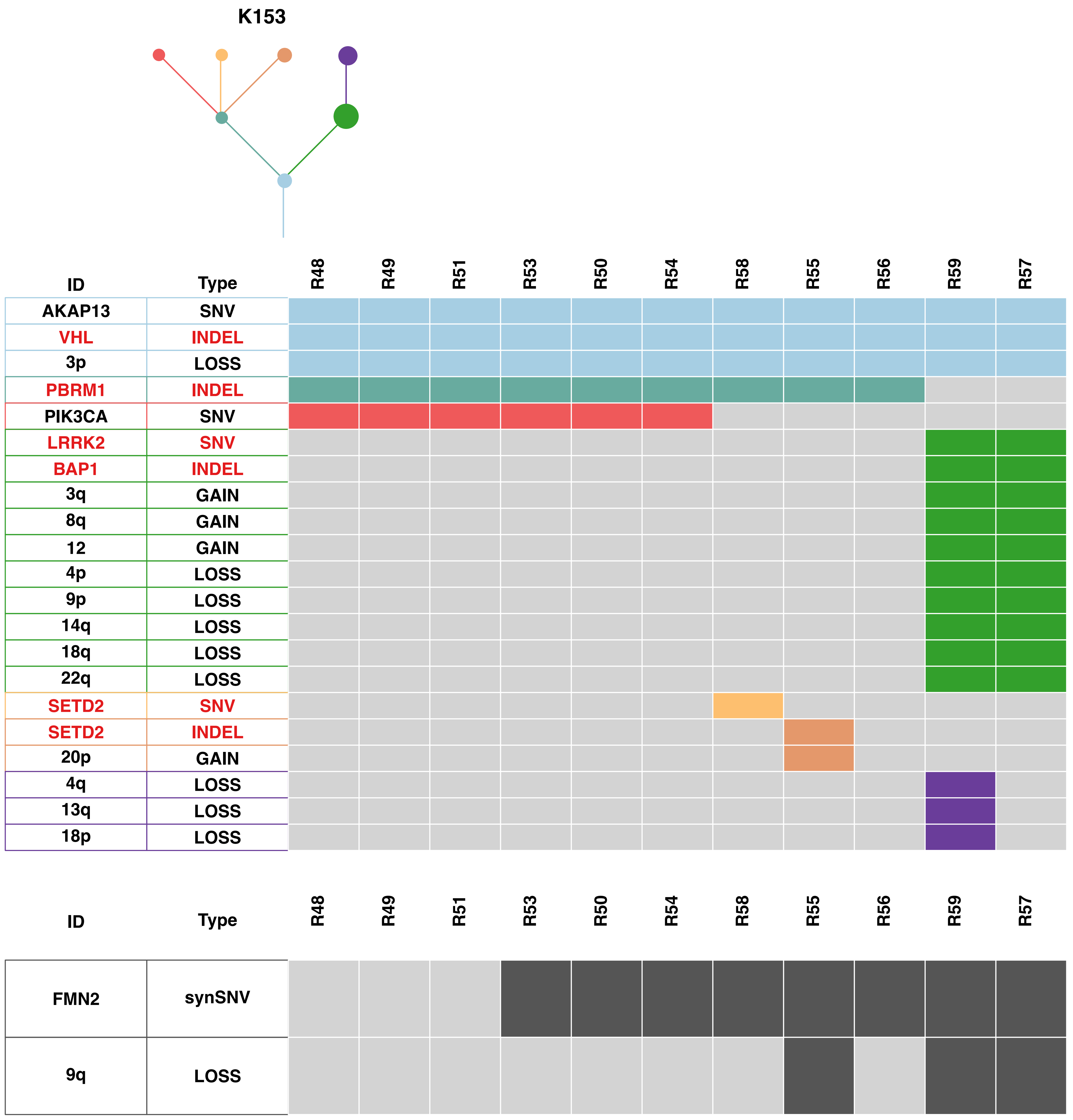

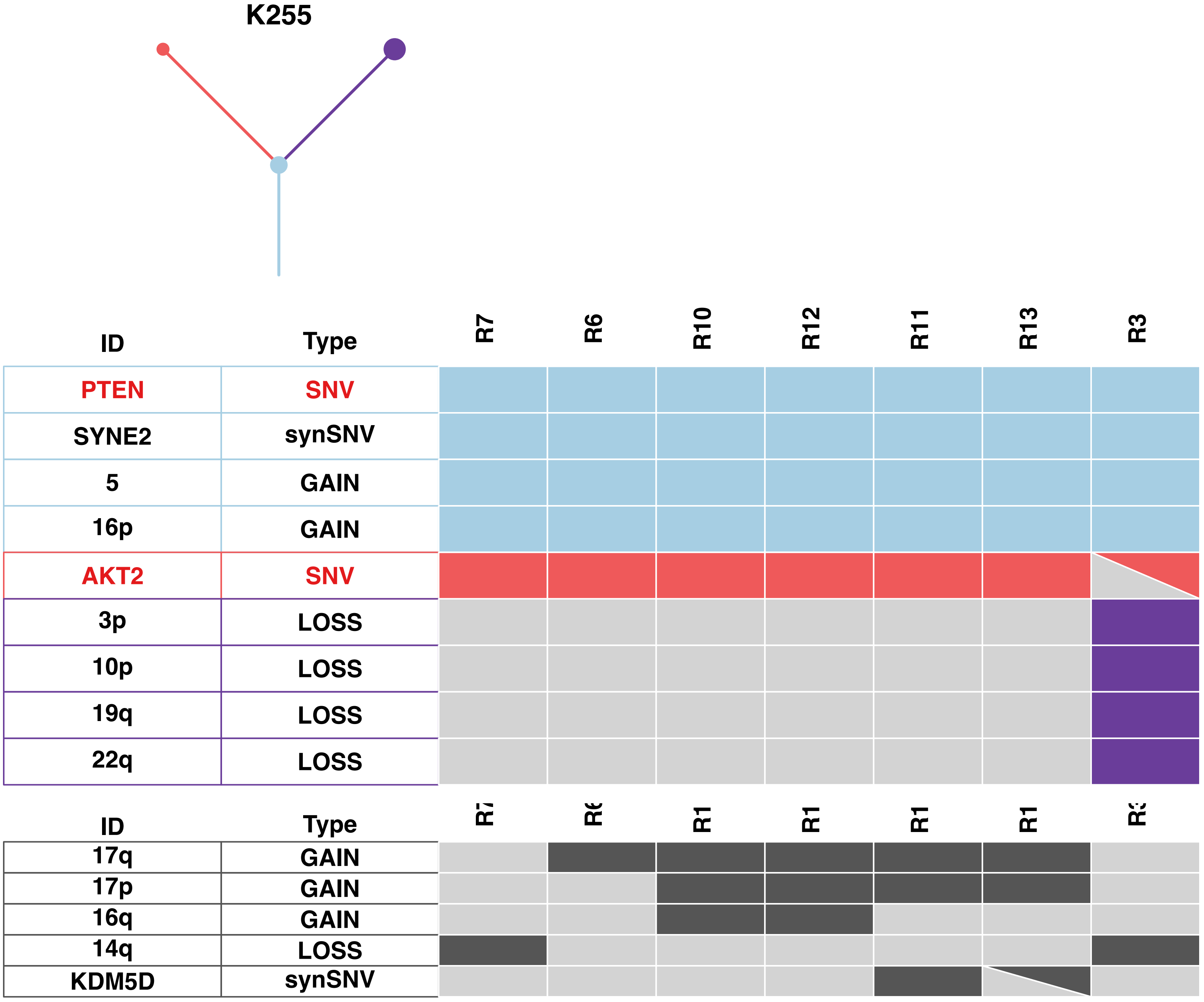
K255
ID
Type
R7
R6
R10
R12
R11
R13
R3
PTEN
SNV
SYNE2
synSNV
5
GAIN
16p
GAIN
AKT2
SNV
3p
LOSS
10p
LOSS
19q
LOSS
22q
LOSS
ID
Type
R1
R1
R1
R1
17q
GAIN
17p
GAIN
16q
GAIN
14q
LOSS
KDM5D
synSNV

K448

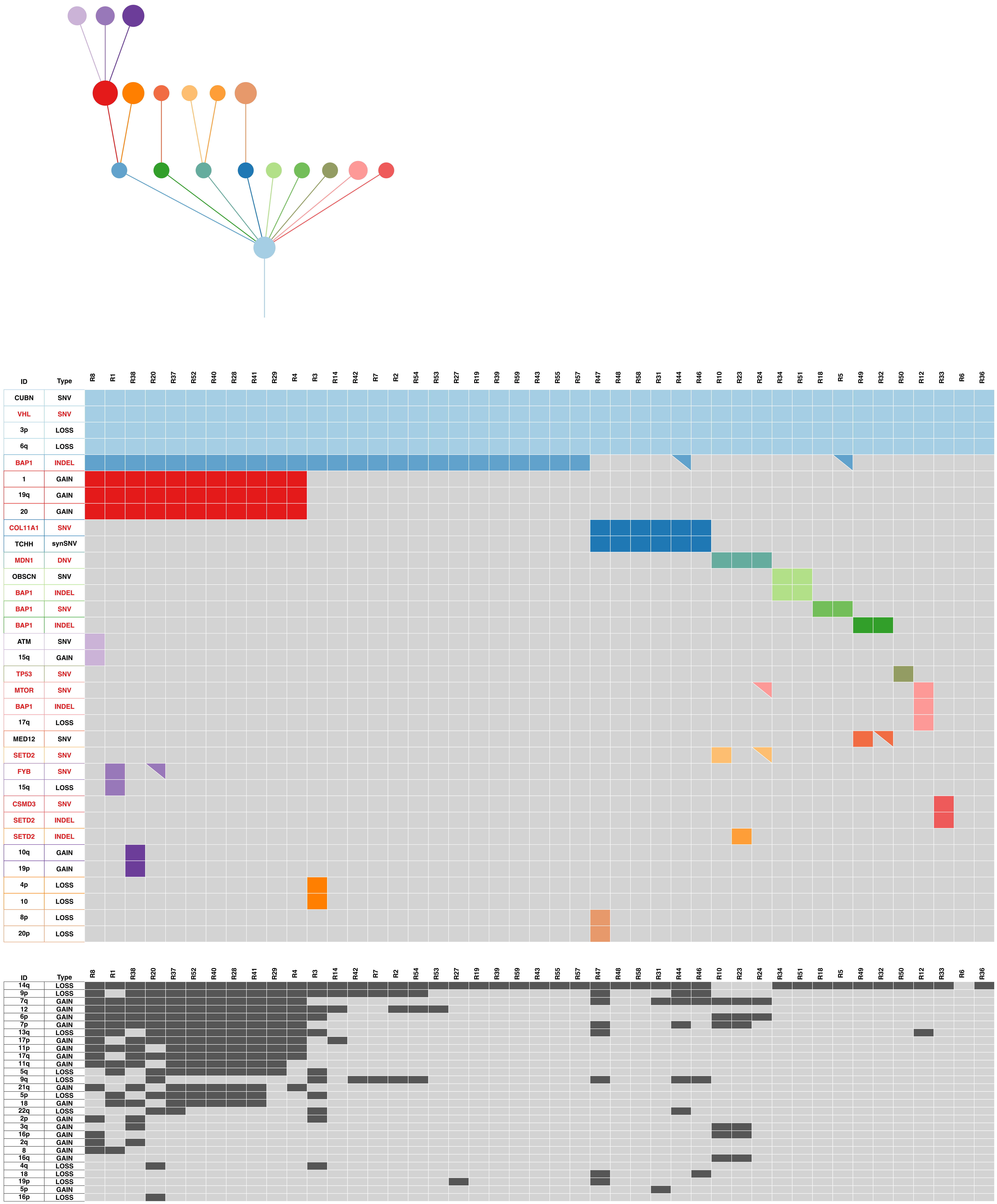
ID
Type
R8
R1
R38
R20
R37
R52
R40
R28
R41
R29
R4
R3
R14
R42
R7
R2
R54
R53
R27
R19
R39
R59
R43
R55
R57
R47
R48
R58
R31
R44
R46
R10
R23
R24
R34
R51
R18
R5
R49
R32
R50
R12
R33
R6
R36
CUBN SNV
VHL SNV
3p LOSS
6q LOSS
BAP1 INDEL
1 GAIN
19q GAIN
20 GAIN
COL11A1 SNV
TCHH synSNV
MDN1 DNV
OBSCN SNV
BAP1 INDEL
BAP1 SNV
BAP1 INDEL
ATM SNV
15q GAIN
TP53 SNV
MTOR SNV
BAP1 INDEL
17q LOSS
MED12 SNV
SETD2 SNV
FYB SNV
15q LOSS
CSMD3 SNV
SETD2 INDEL
SETD2 INDEL
10q GAIN
19p GAIN
4p LOSS
10 LOSS
8p LOSS
20p LOSS
ID
Type
14q LOSS
9p LOSS
7q GAIN
12 GAIN
6p GAIN
7p GAIN
13q LOSS
17p GAIN
11p GAIN
17q GAIN
11q GAIN
5q LOSS
9q LOSS
21q GAIN
5p LOSS
18 GAIN
22q LOSS
2p GAIN
3q GAIN
16p GAIN
2q GAIN
8 GAIN
16q GAIN
4q LOSS
18 LOSS
19p LOSS
5p GAIN
16p LOSS

K252

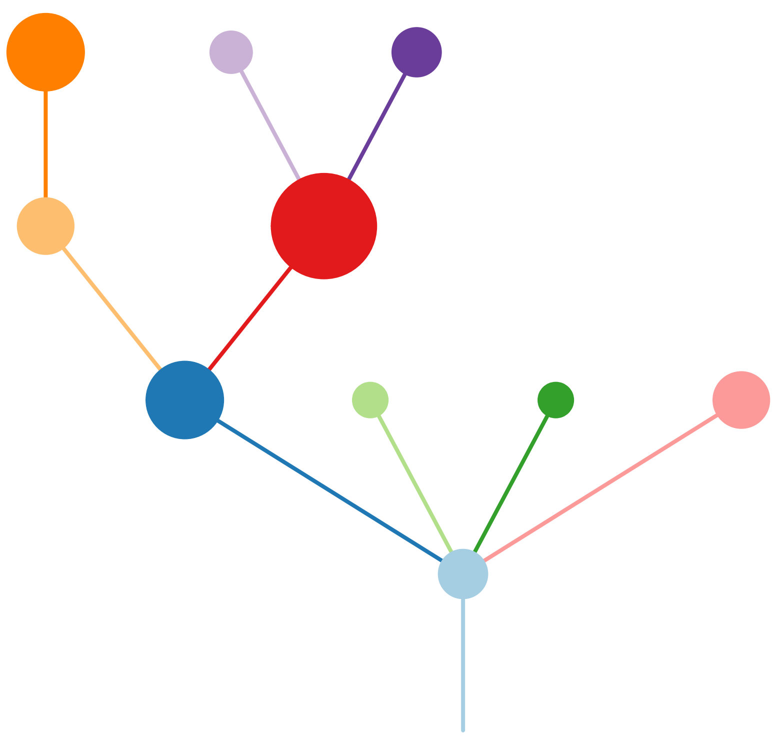

ID	Type	R1	R5	R10	R9	R4	R3	R8
VHL	SNV							
PBRM1	SNV							
7	GAIN							
6	LOSS							
4	LOSS							
10q	LOSS							
11	LOSS							
14q	LOSS							
17p	LOSS							
18p	LOSS							
DNAH10	synSNV							
TLN2	SNV							
TP53	SNV							
LRP2	SNV							
BAP1	SNV							
PLEC	SNV							
3p	GAIN							
8q	GAIN							
9	GAIN							
10p	GAIN							
12p	GAIN							
1p	LOSS							
8p	LOSS							
13q	LOSS							
18q	LOSS							
20p	LOSS							
2p	GAIN							
19q	GAIN							
20q	GAIN							
USH2A	SNV							
OBSCN	SNV							
BAP1	INDEL							
SYNE1	SNV							
BAP1	INDEL							
21q	GAIN							
9	LOSS							
17q	LOSS							
CSMD3	SNV							
SETD2	INDEL							
2q	GAIN							
17q	GAIN							
20p	GAIN							
10p	LOSS							
21q	LOSS							
22q	LOSS							
DNAH3	INDEL							
22q	GAIN							
12q	GAIN							
19q	LOSS							

ID	Type	R1	R5	R10	R9	R4	R3	R8
3	LOSS							
5	GAIN							
19p	LOSS							

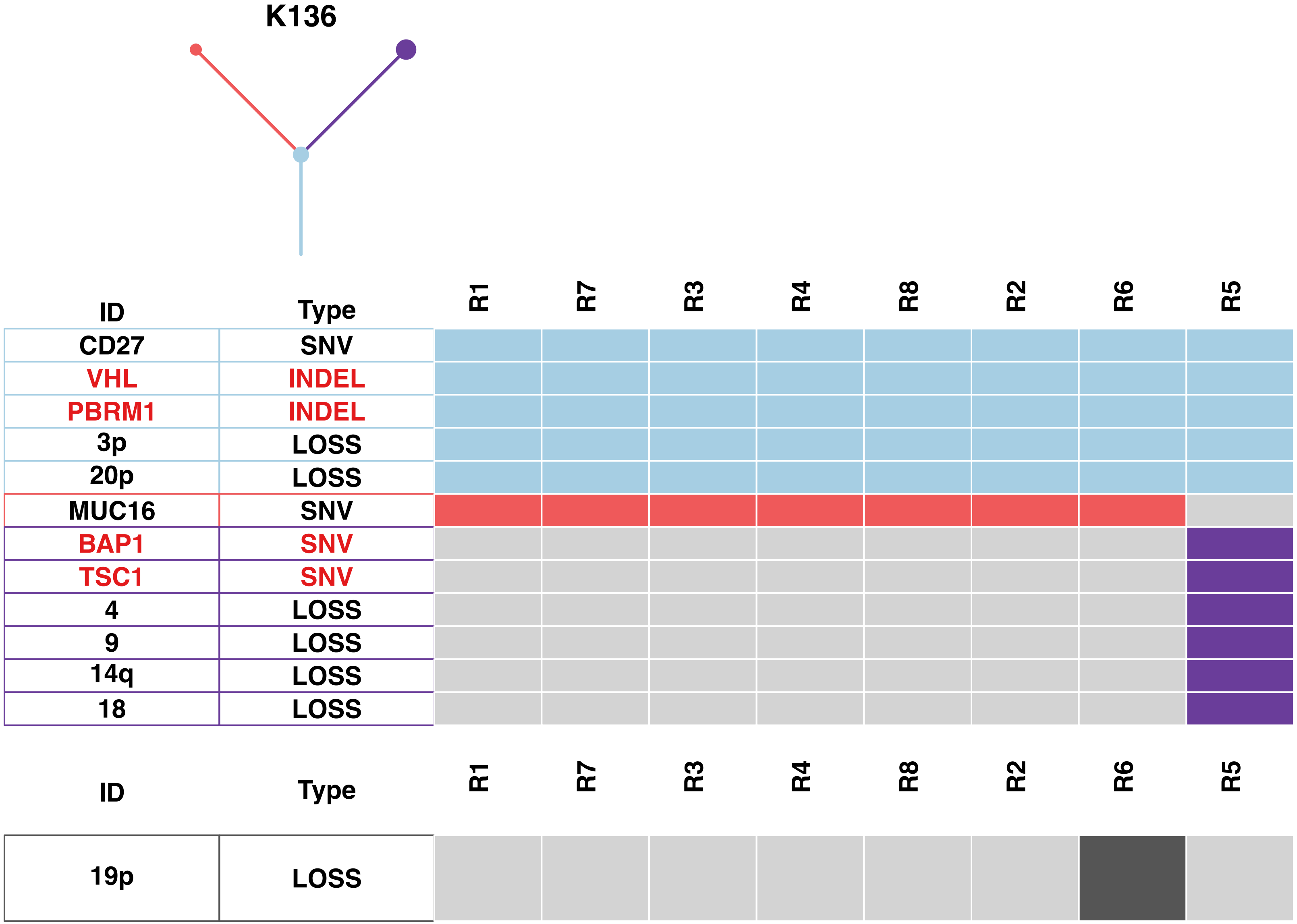
K136
ID
Type
R1
R7
R3
R4
R8
R2
R6
R5
CD27
SNV
VHL
INDEL
PBRM1
INDEL
3p
LOSS
20p
LOSS
MUC16
SNV
BAP1
SNV
TSC1
SNV
4
LOSS
9
LOSS
14q
LOSS
18
LOSS
ID
Type
R1
R7
R3
R4
R8
R2
R6
R5
19p
LOSS
