# Supplementary material for: Deterministic Evolutionary Trajectories Influence Primary Tumor Growth: TRACERx Renal
Source: Cell. 2018 Apr 19;173(3):595–610.e11. doi: 10.1016/j.cell.2018.03.043 (PMC5938372; doi:10.1016/j.cell.2018.03.043)
Supplement: Data S3. Copy Neutral Allelic Imbalance, MSAI, and SCNA Profiles for Bilateral and Multifocal Tumors, Related to Figures 1 and 3 and STAR Methods — Page 1 shows copy neutral allelic imbalance data. Pages 2-17 shows case level MSAI results. Page 18 shows a summary of MSAI results. Pages 19-20 shows MSAI validation results. Page 21 shows SCNA profiles for bilateral and multifocal cases, with each row represents an individual tumour region; blue represents copy number loss, red represents copy number gain. [file mmc8.pdf]

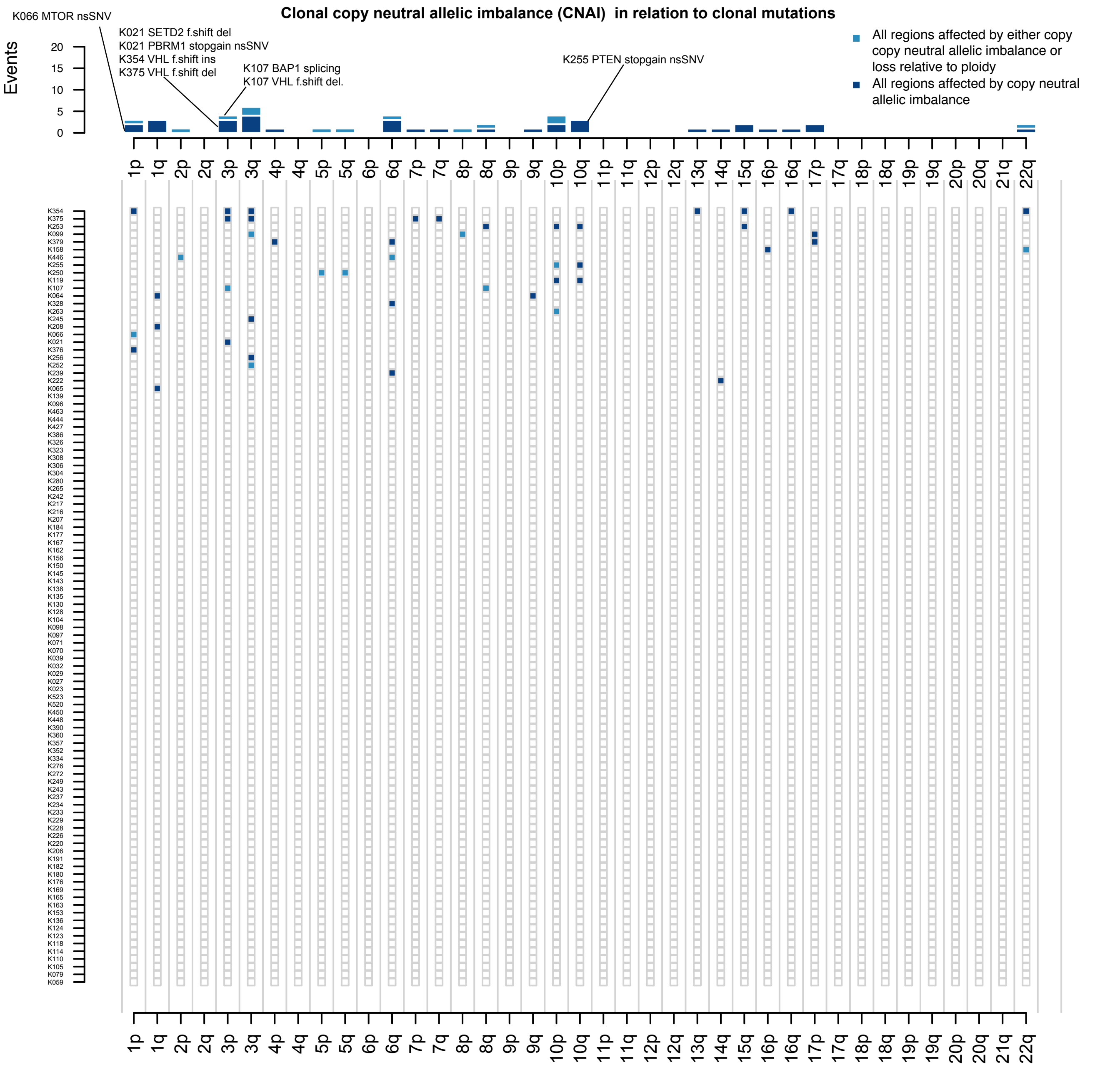

The top panel of this figure is bar chart depicting the number of ubiquitous (present in all regions sequenced for a patient) copy neutral allelic imbalance (CNAI) events in dark blue and the number of ubiquitous CNAI/loss in the cohort for all chromosome arms is indicated in light blue. Patients with co-occurring clonal mutations on these chromosomal arms are shown by lines to the corresponding chromosome arms. The bottom panel is a heatmap indicating which patients' disease from the cohort have either ubiquitous CNAI events (dark blue) or ubiquitous CNAI/loss events (light blue).

Patient K059

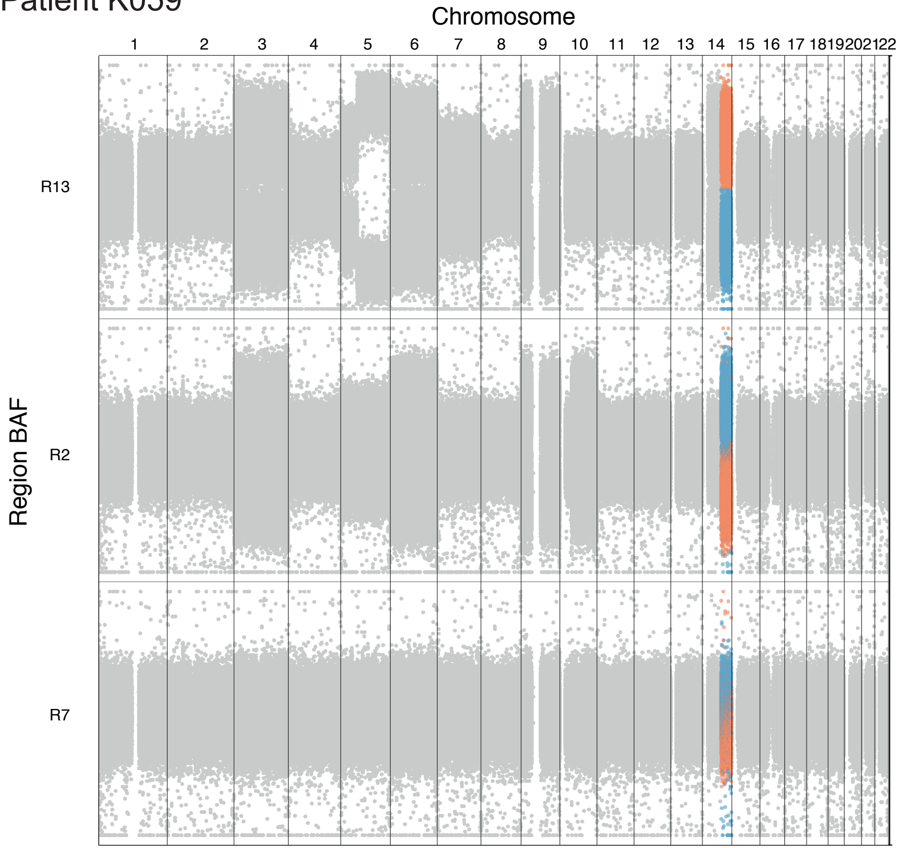

B-allele frequency profile of heterozygous SNPs across the genome (chromosomes 1-22) from all regions obtained from the multi-region whole genome sequencing from the patient's disease. Sections of BAF in regions that have mirrored subclonal allelic imbalance are highlighted in blue or orange. This legend applies to the ther patient specific figures of the same format in this document.

Patient K071

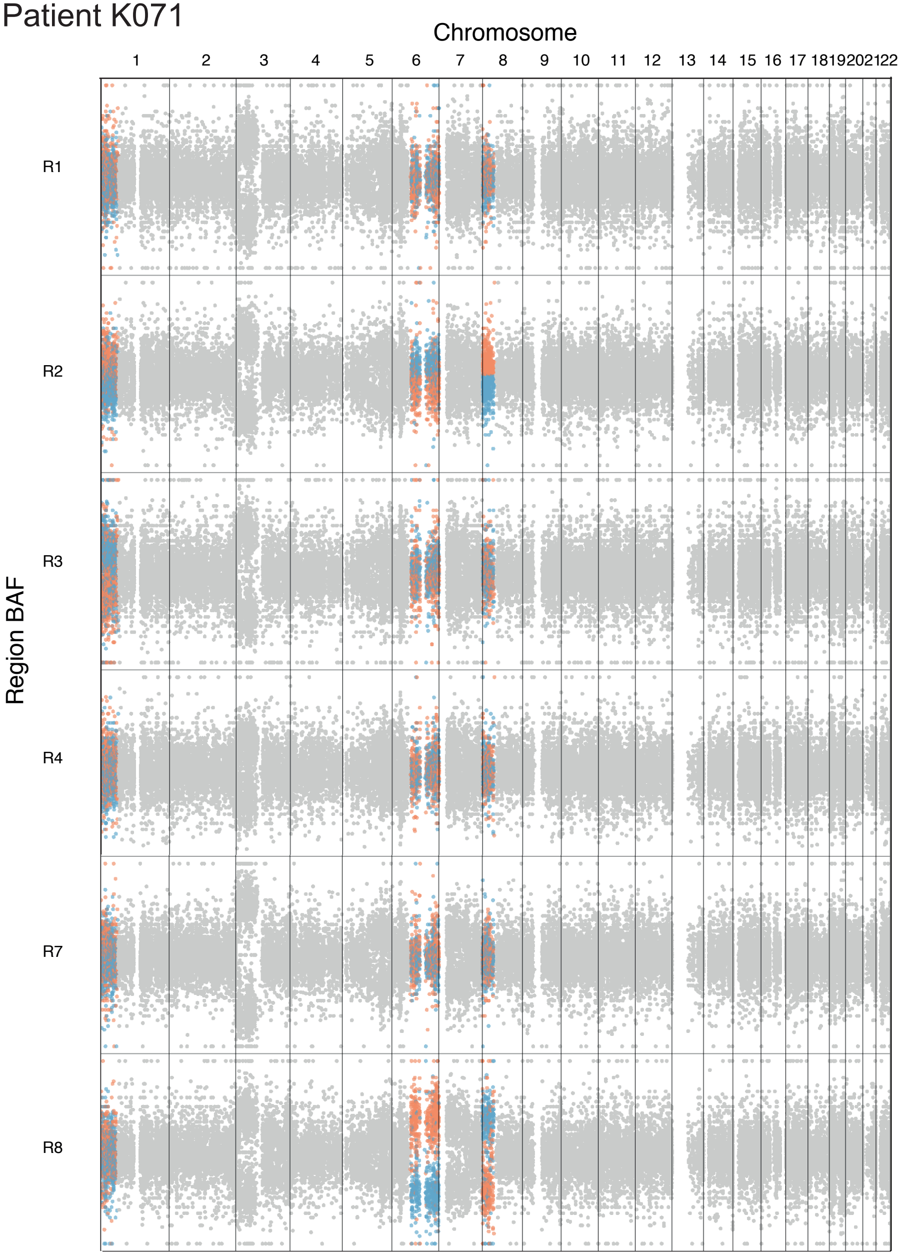

Patient K099

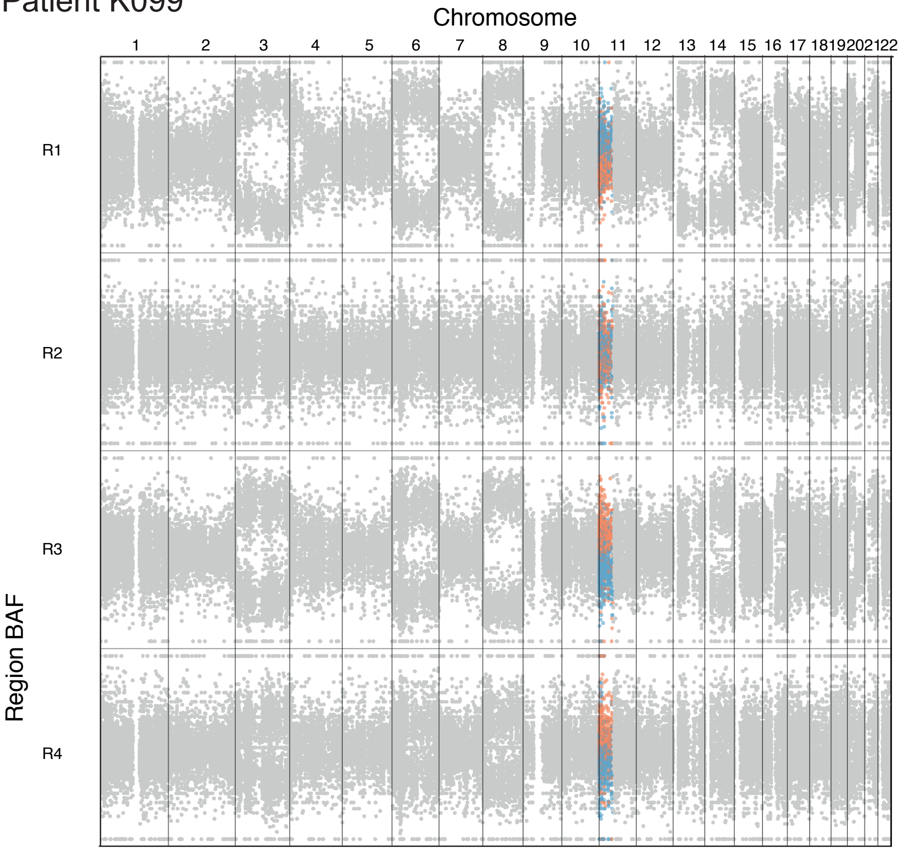

Patient K104 focal
chr8:30242747-47499801

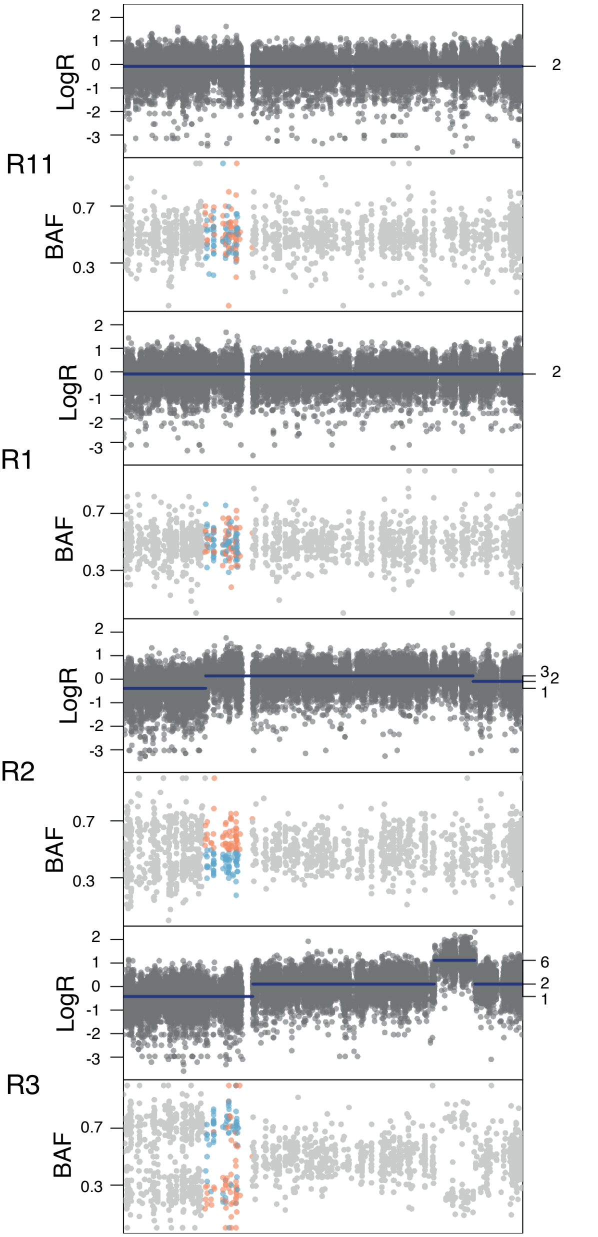

Shows the BAF and LogR (log ratio of the relative levels of sequencing coverage in the tumor region versus the germline normal) profile across part of chromosome 8 for all regions of the tumor samples that underwent whole exome sequencing from patient’s disease. Each region has a plot of LogR below with the total copy number assigned to each segment on right of the plot, it also has a BAF plot below. The BAF in regions that have mirrored subclonal allelic imbalance are highlighted in blue or orange. This legend applies to the ther patient specific figures of the same format in this document.

Patient K107

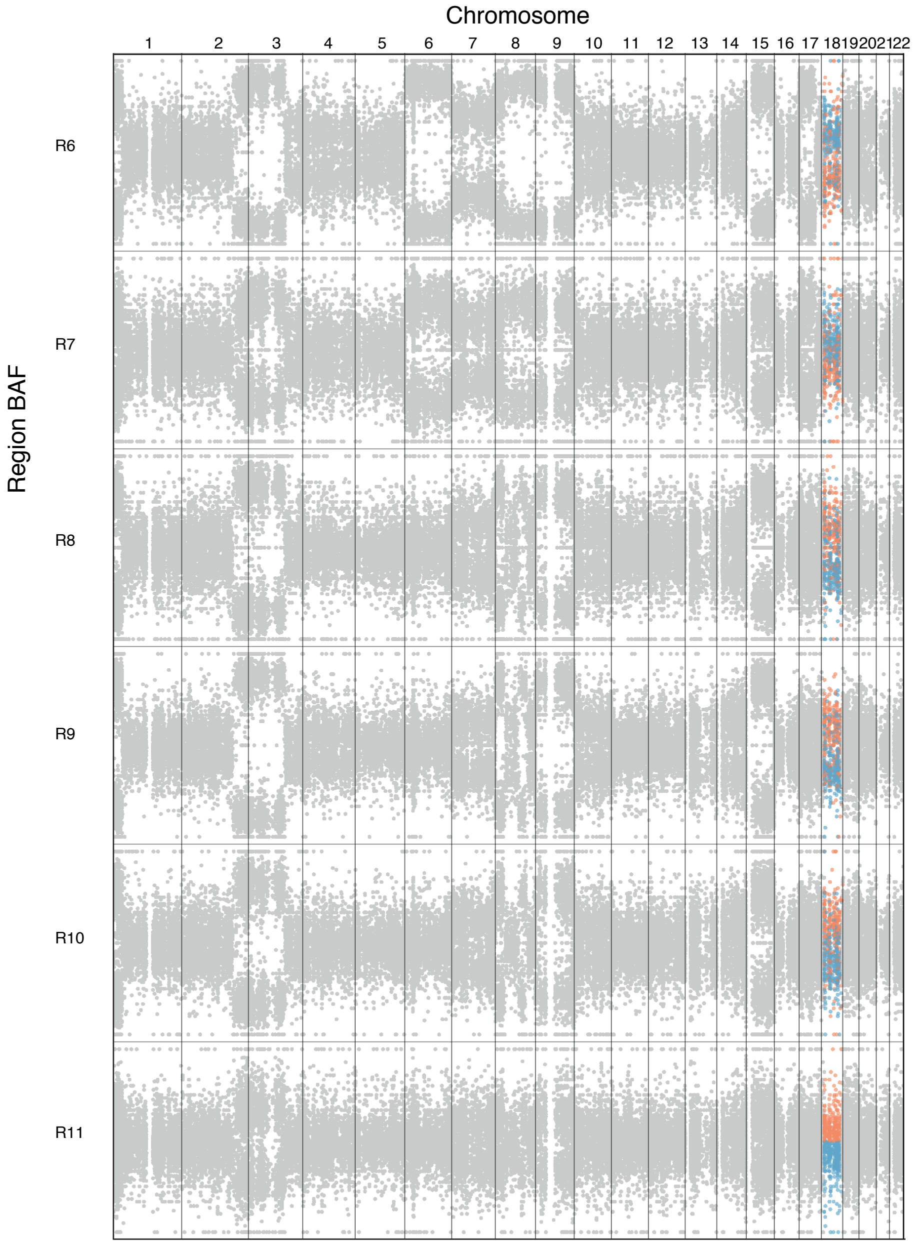

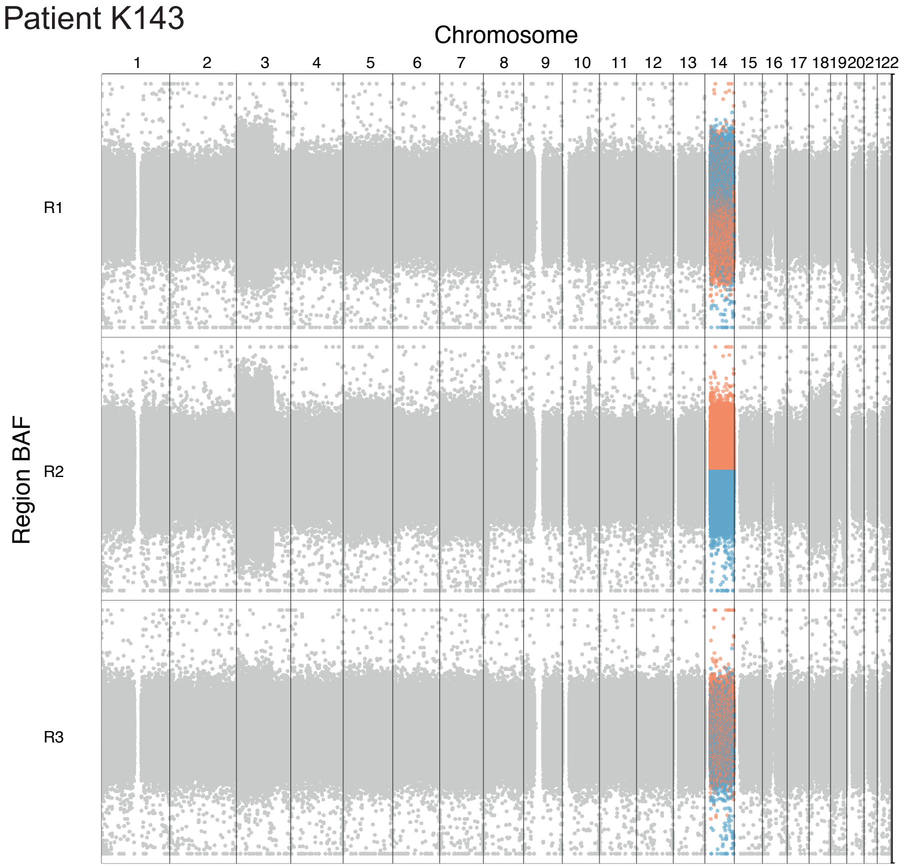
Patient K143
Chromosome
1
2
3
4
5
6
7
8
9
10
11
12
13
14
15
16
17
18
19
20
21
22
R1
R2
R3
Region BAF

Patient K150

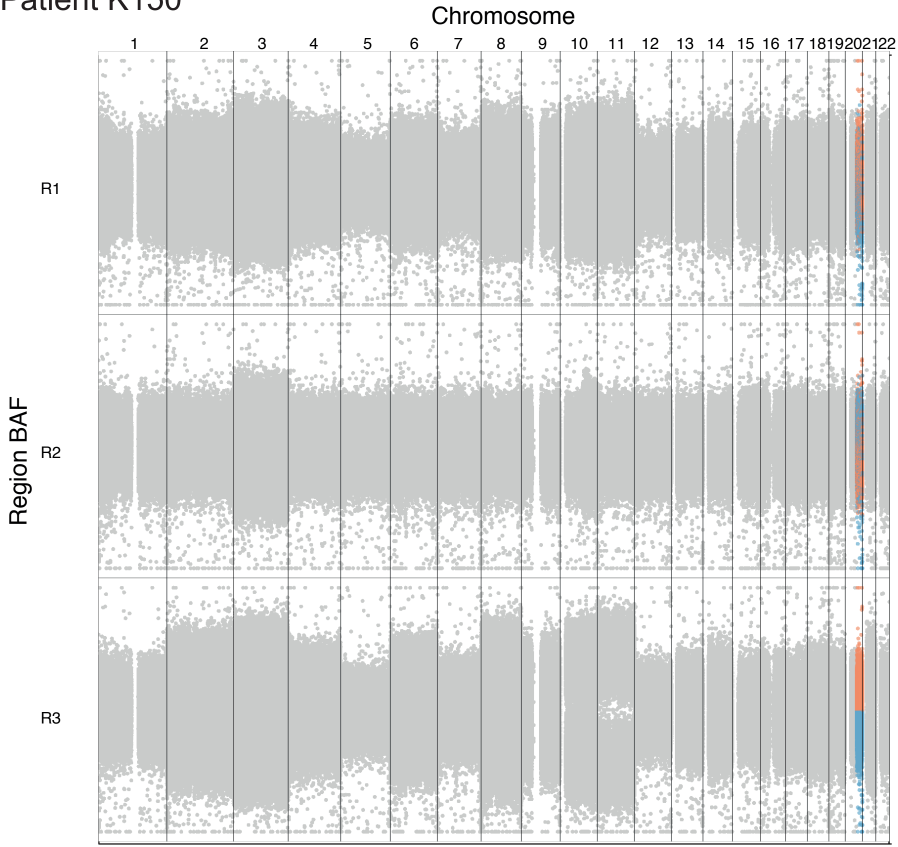

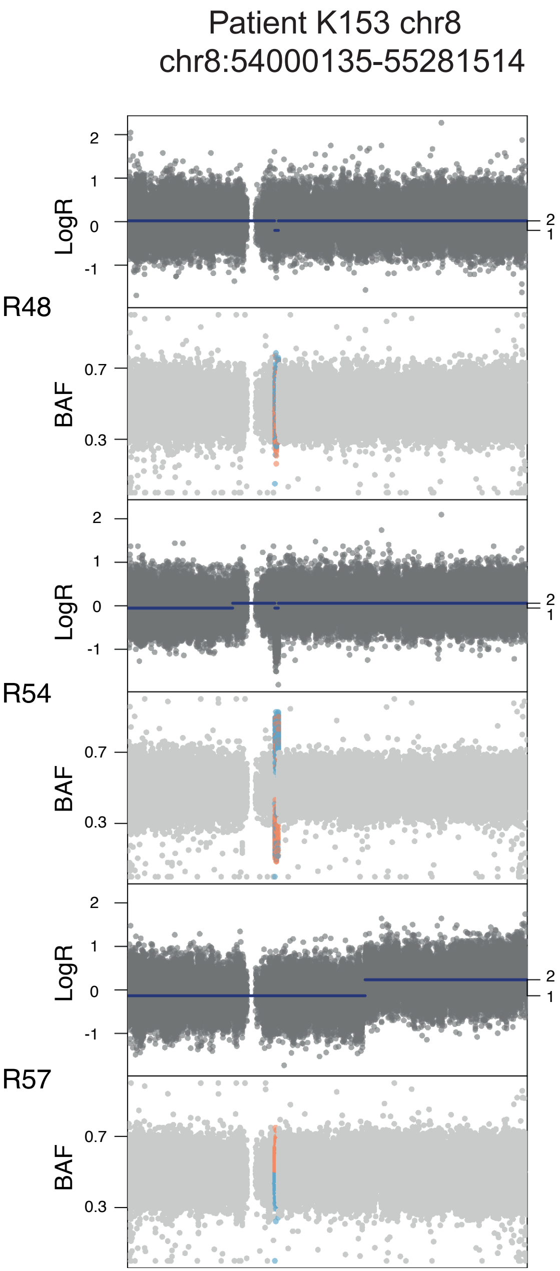
Patient K153 chr8
chr8:54000135-55281514
R48
R54
R57
LogR
BAF
2
1
0
-1
0.7
0.3

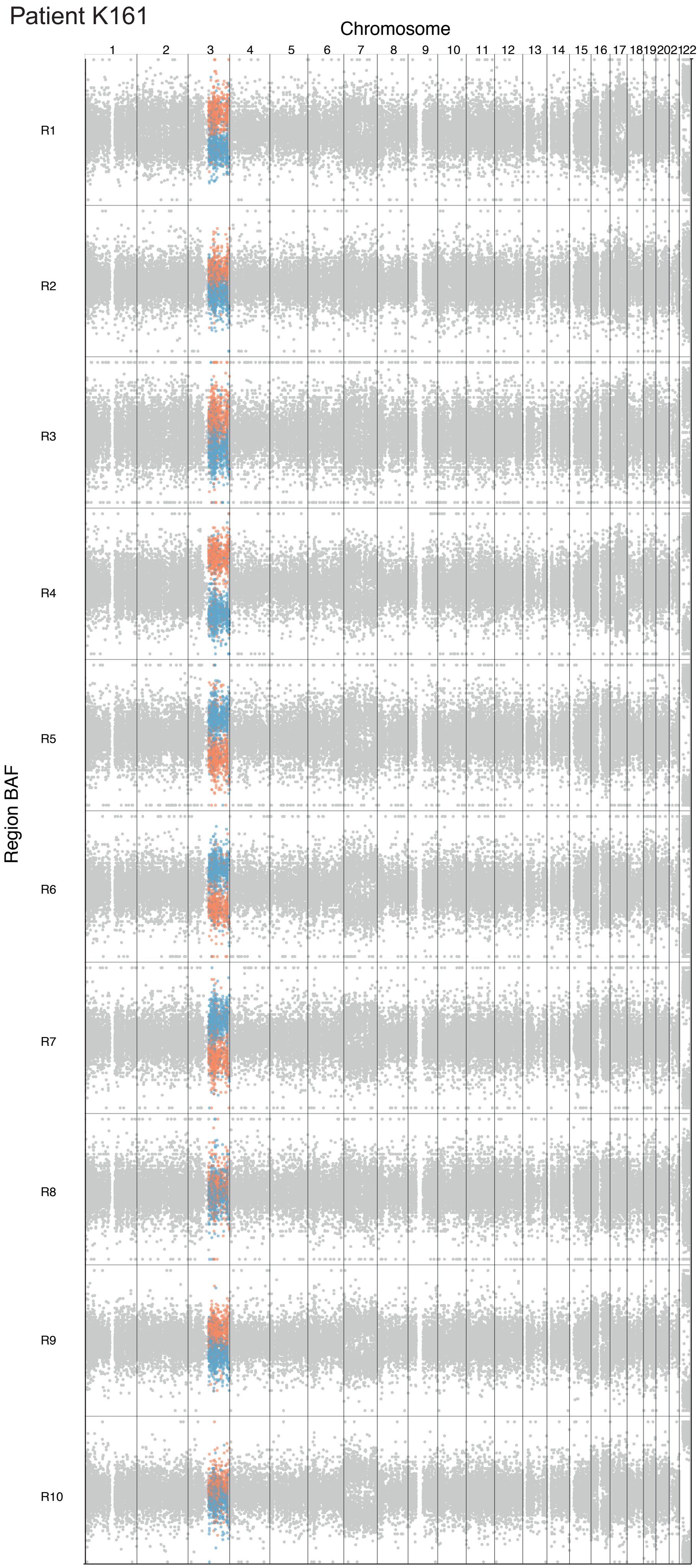
Patient K161
Chromosome
1
2
3
4
5
6
7
8
9
10
11
12
13
14
15
16
17
18
19
20
21
22
Region BAF
R1
R2
R3
R4
R5
R6
R7
R8
R9
R10

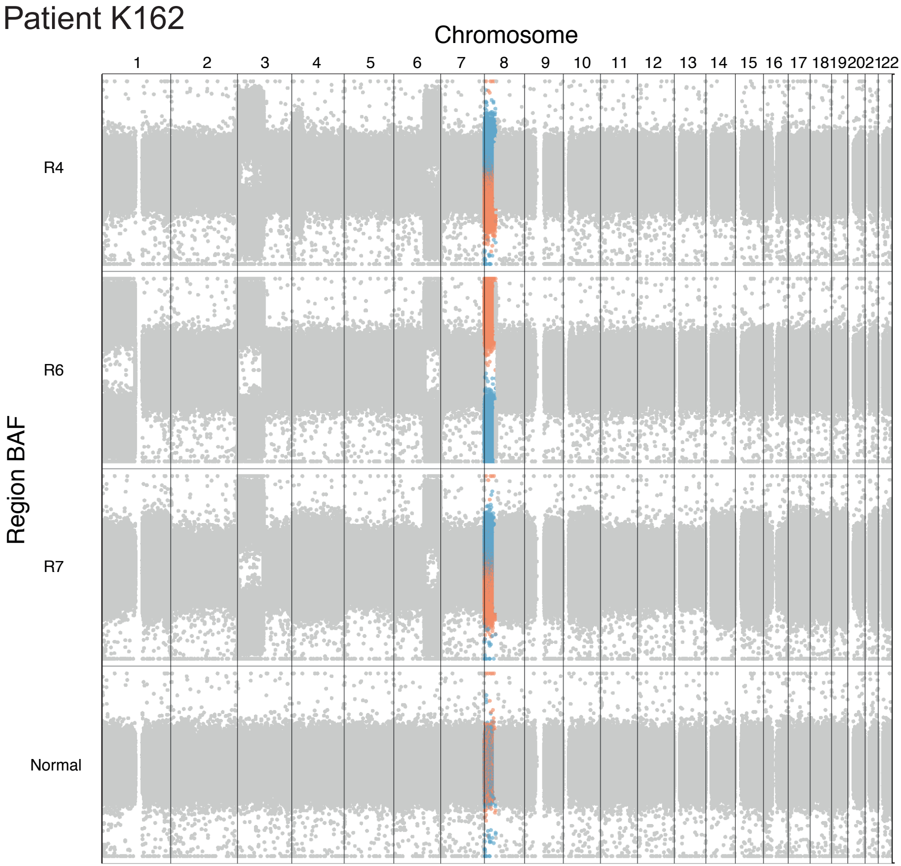
Patient K162
Chromosome
1
2
3
4
5
6
7
8
9
10
11
12
13
14
15
16
17
18
19
20
21
22
Region BAF
R4
R6
R7
Normal

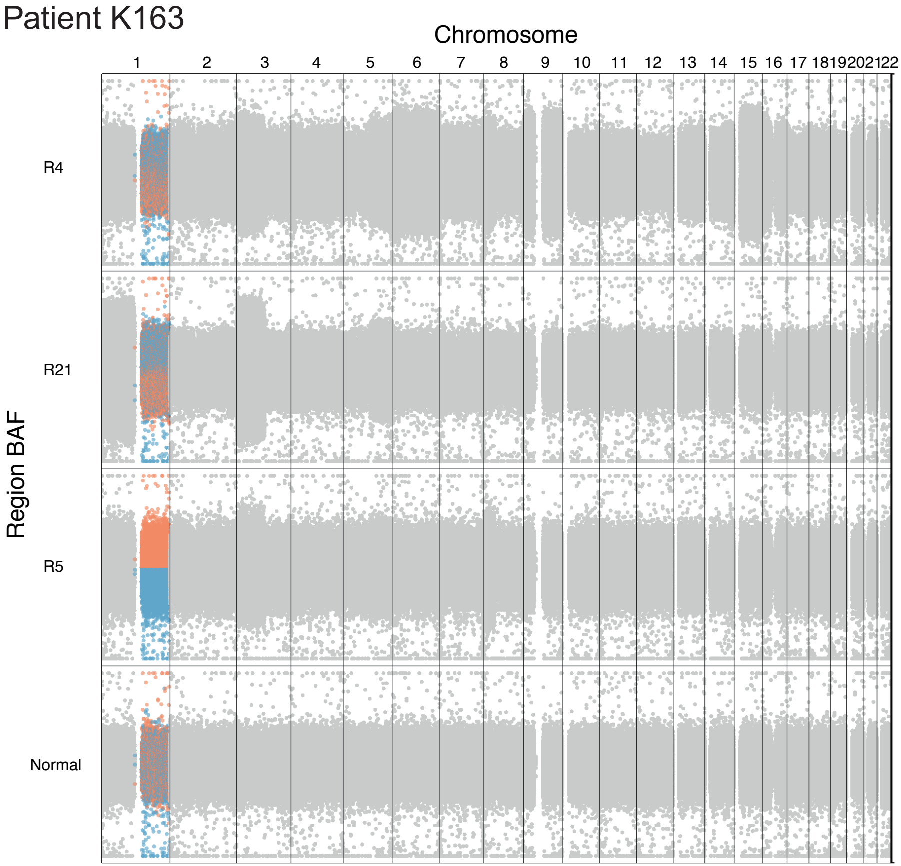
Patient K163
Chromosome
1
2
3
4
5
6
7
8
9
10
11
12
13
14
15
16
17
18
19
20
21
22
Region BAF
R4
R21
R5
Normal

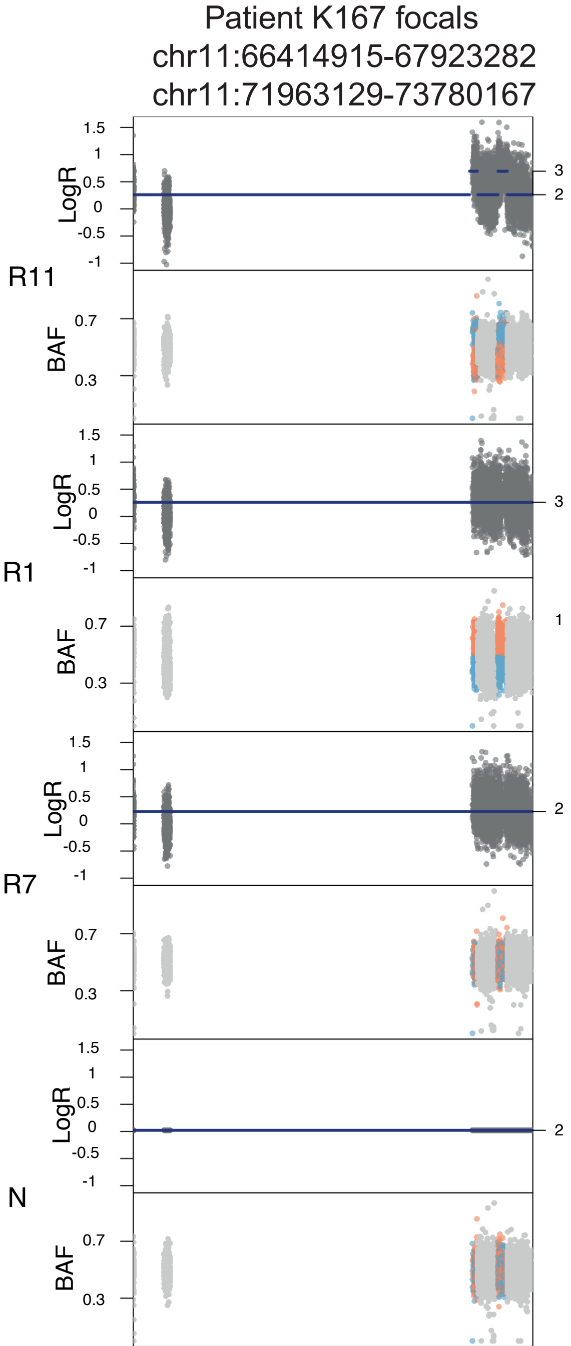

Patient K167 focals
chr11:66414915-67923282
chr11:71963129-73780167
R11
LogR
BAF
R1
LogR
BAF
R7
LogR
BAF
N
LogR
BAF
1.5
1
0.5
0
-0.5
-1
0.7
0.3
3
2
1

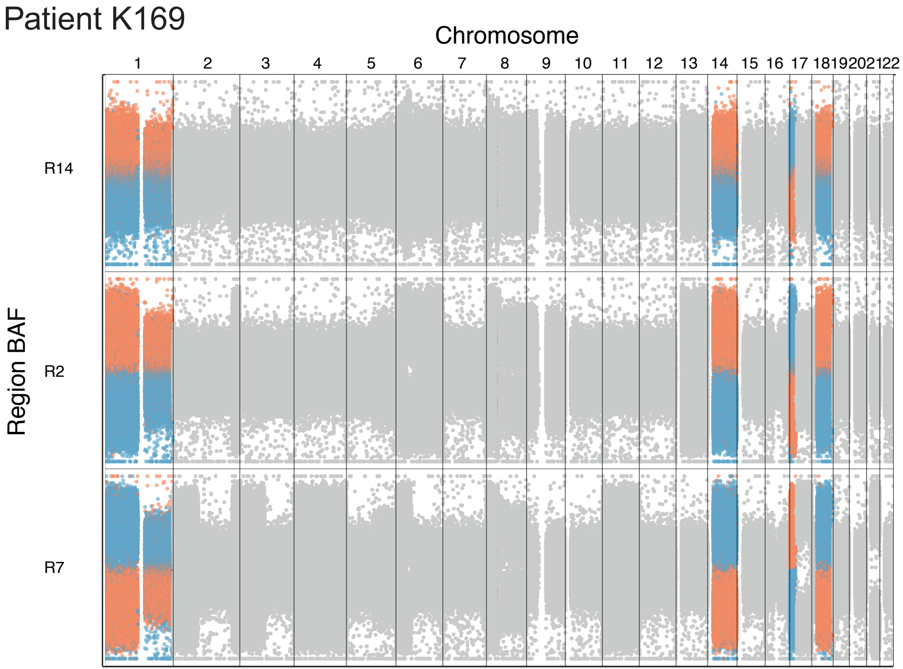
Patient K169
Chromosome
1
2
3
4
5
6
7
8
9
10
11
12
13
14
15
16
17
18
19
20
21
22
Region BAF
R14
R2
R7

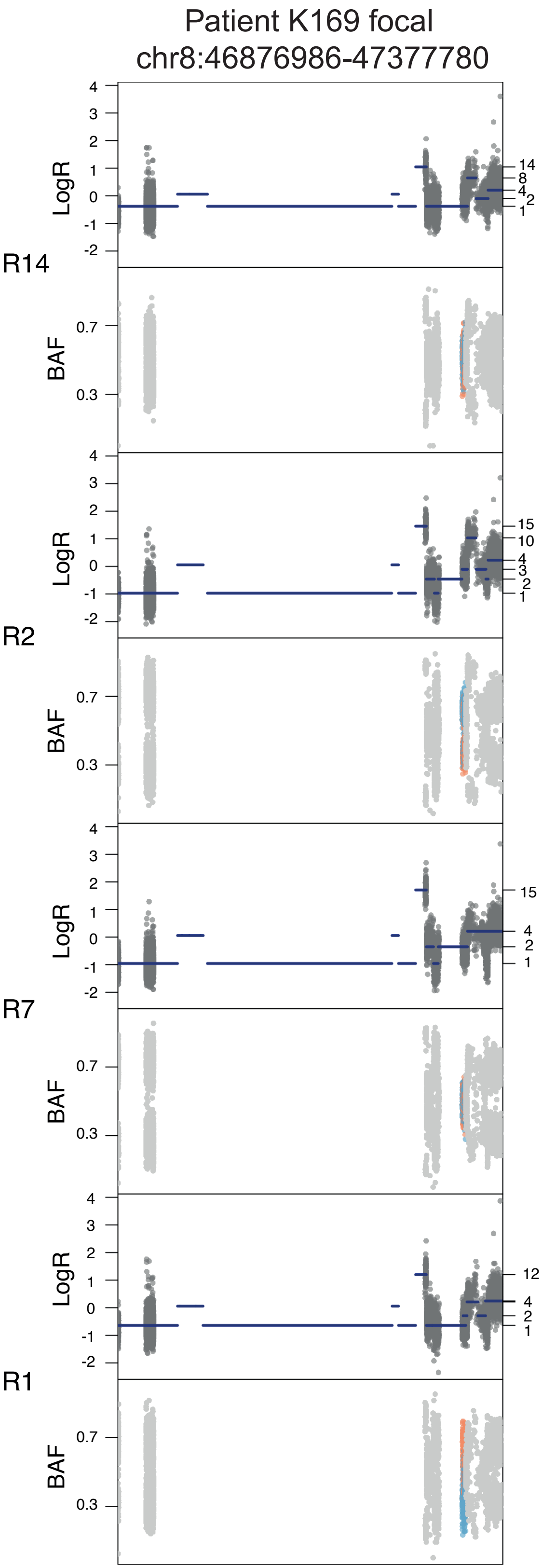
Patient K169 focal
chr8:46876986-47377780
R14
LogR
BAF
R2
LogR
BAF
R7
LogR
BAF
R1
LogR
BAF
14
8
4
2
1
15
10
3
12

Patient K178

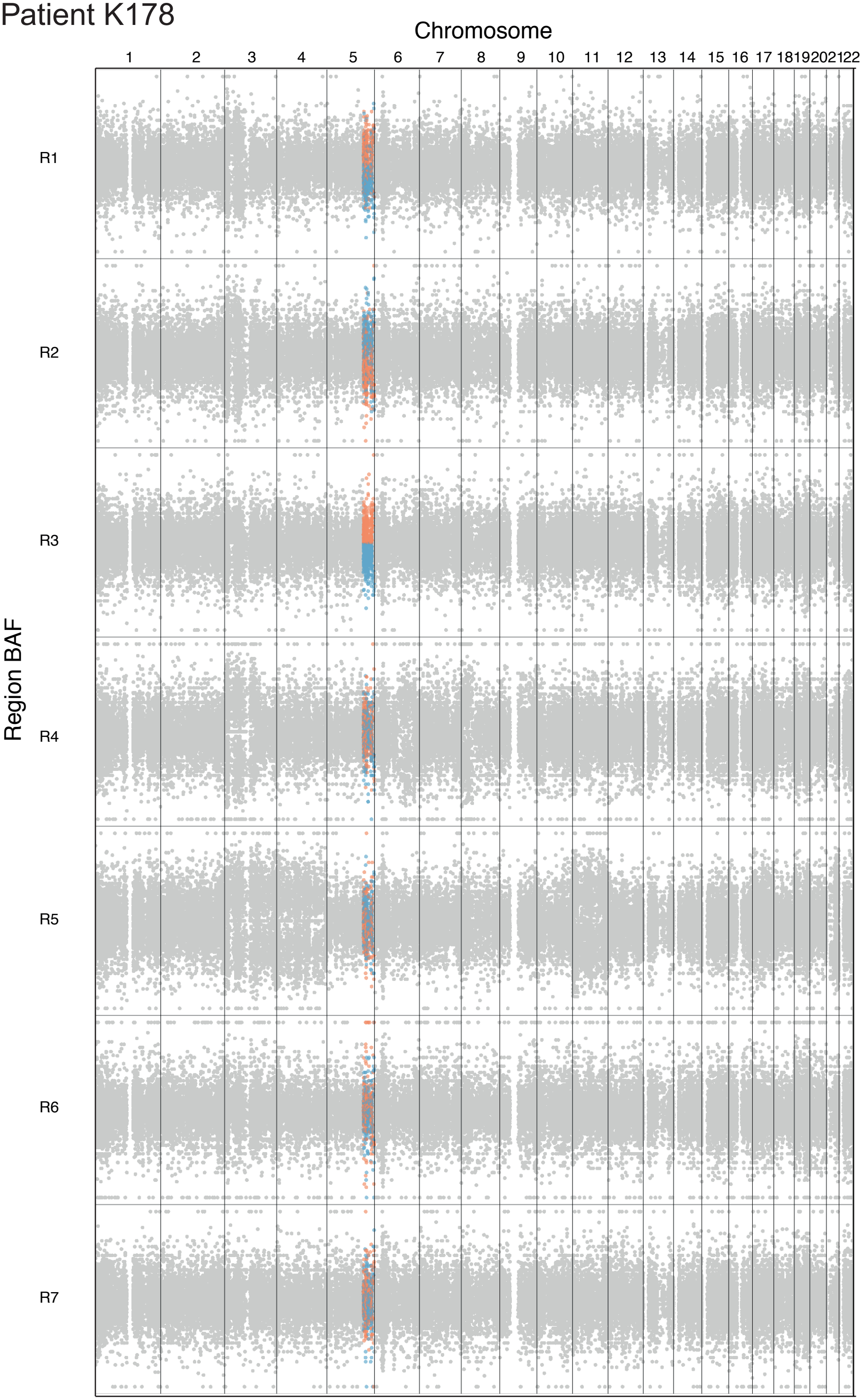

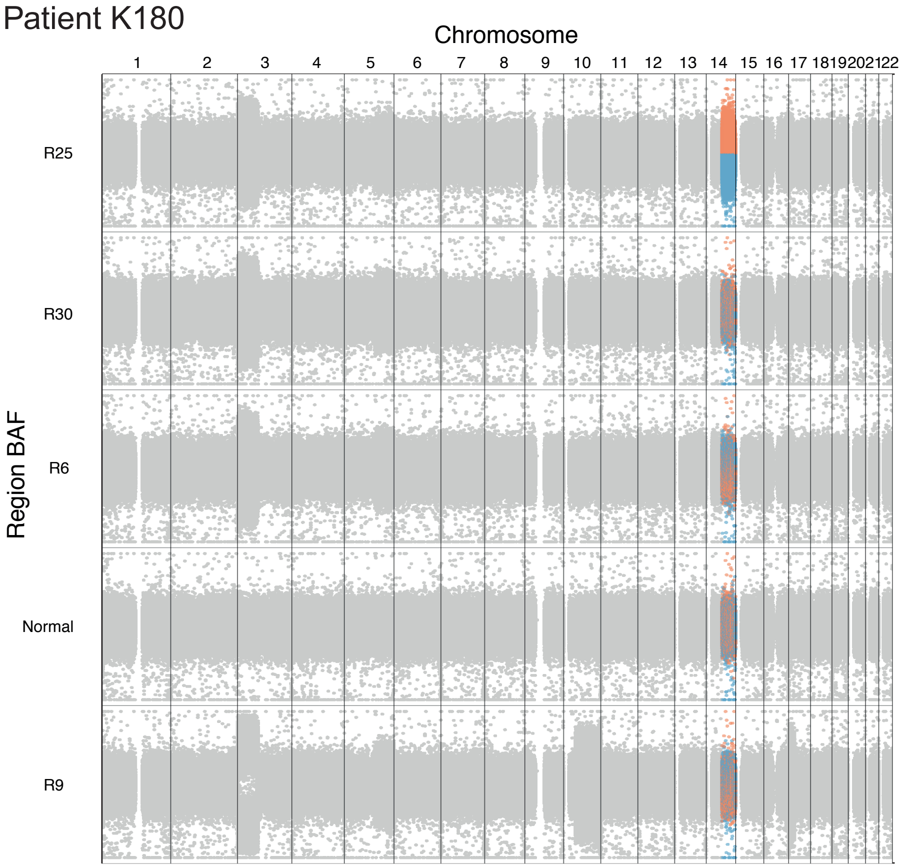
Patient K180
Chromosome
1
2
3
4
5
6
7
8
9
10
11
12
13
14
15
16
17
18
19
20
21
22
Region BAF
R25
R30
R6
Normal
R9

MSAI Summary

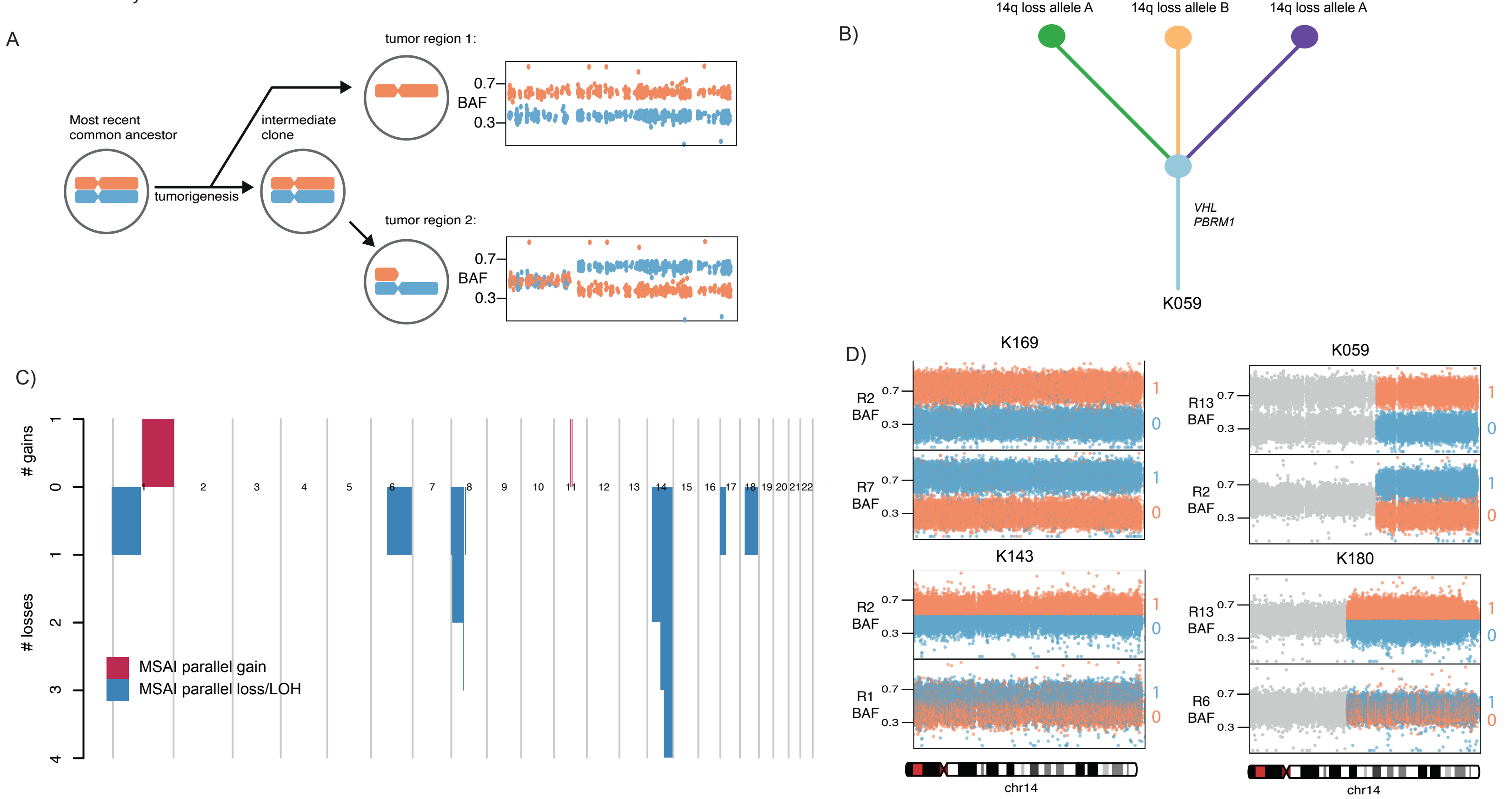

A) Shows a schematic of how a parallel copy number losses could result in mirrored subclonal allelic imbalance (MSAI). This occurs when the maternal allele is gained or lost in a subclone in one region and the paternal allele is gained or lost in a different subclone in another region. In the schematic the entire paternal (blue) chromosome is lost in R1 leading to the allelic imbalance seen in B-allele frequency (BAF) plot with the heterozygous SNPs' from the paternal (blue) chromosome having higher BAF values. In R2 the q arm of the maternal (orange) chromosome is lost leading to isolated allelic imbalance in the plot to the right with the heterozygous SNPs originating from q arm paternal q arm having higher BAF values. B) Phylogenetic tree indicating the subclonal loss of 14q from different alleles in the three regions that underwent whole genome sequencing. C) Shows the genomic position and size of all MSAI events found in the multi-region exome and multi-region whole genome sequencing performed in this study. This includes MSAI events that result from heterogeneous gains and losses relative to ploidy in same patient's disease. MSAI parallel loss of 14q is the most common event. D) shows the B-allele frequency (BAF) plots of two regions from each patient's disease that demonstrates MSAI through parallel loss relative to ploidy and loss of heterozygosity in chromosome 14.

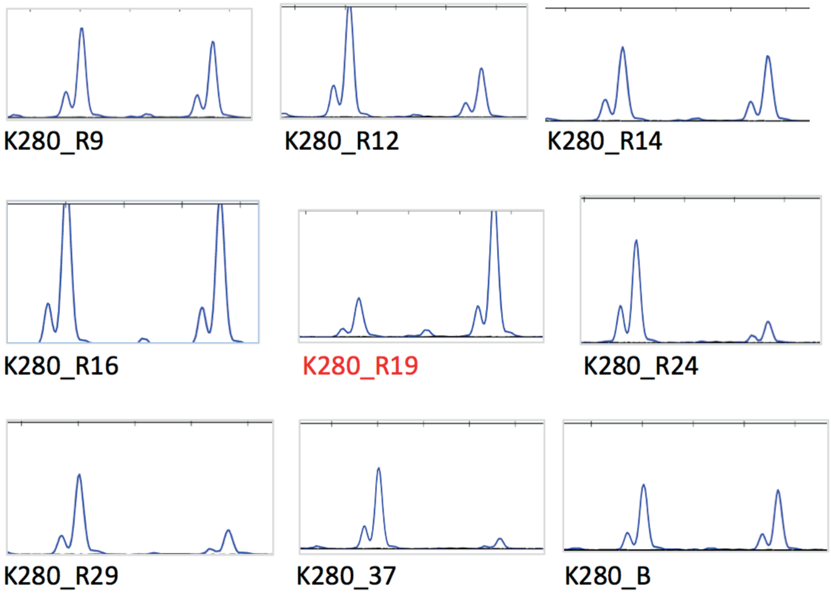

PCR fragment analysis using microsatellite marker D14S306 validating subclonal loss and MSAI (R19) in patient K280. Each number following the patient identifier indicates a tumor region and the germline is indicated by a ‘B’.

MSAI validation

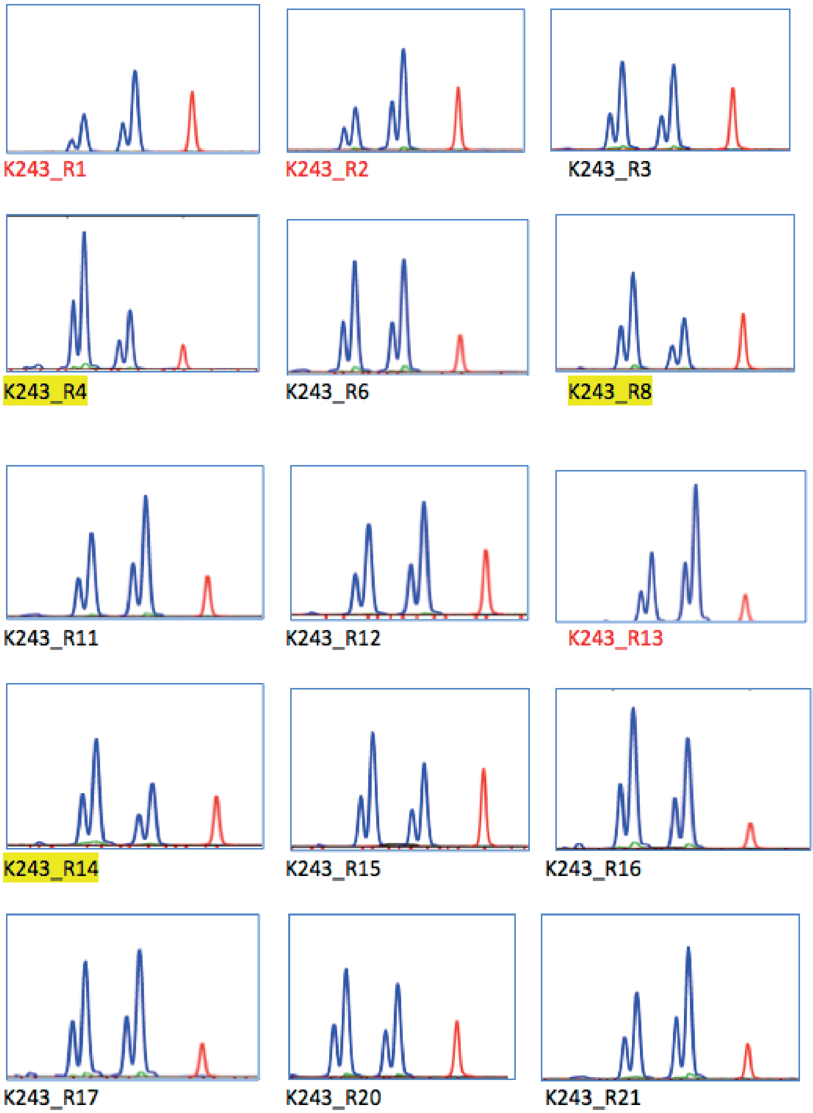

PCR fragment analysis using microsatellite marker D14S306 validating subclonal loss and MSAI (R4, R8 & R14) in patient K243.

K265 (Primary tumour 1: R1, R3; Primary tumour 2: R5 - R8)

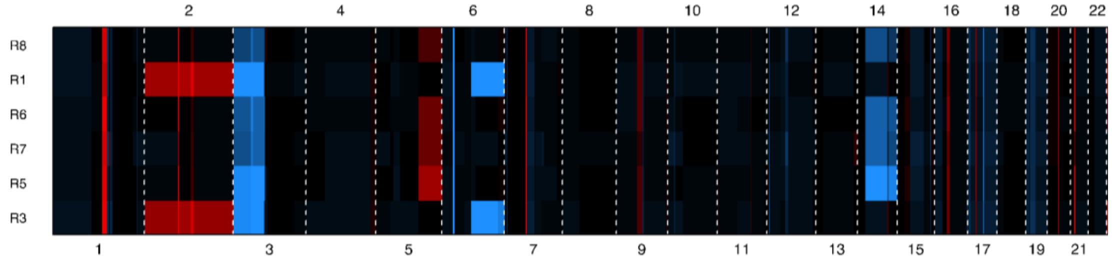

K334 (Left primary tumour: R11 - R13; Right primary tumour: R1, R5 - R8)

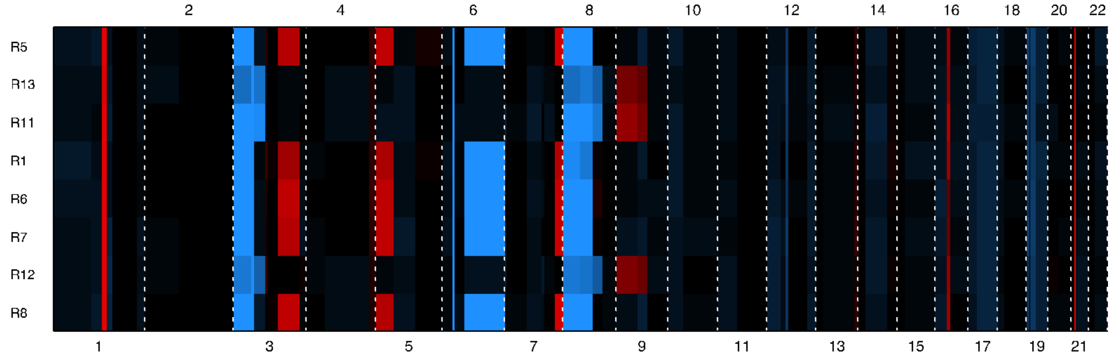

K352 (Primary tumour 1: R1, R2, R3; Primary tumour 2: R4, R5, R6)

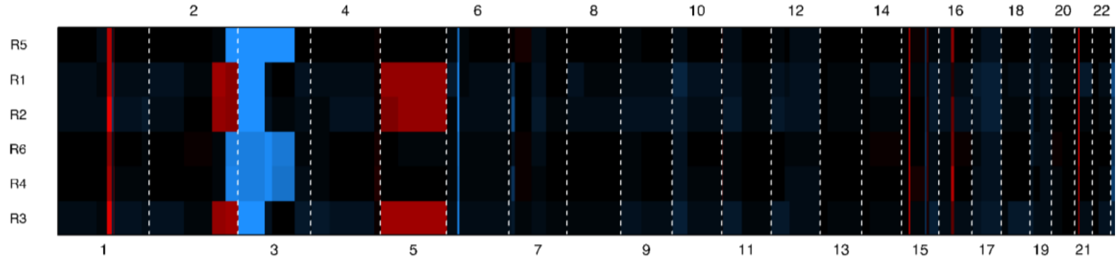

K097 (Left primary tumour: R1-R3; Right primary tumour: R101, R102)

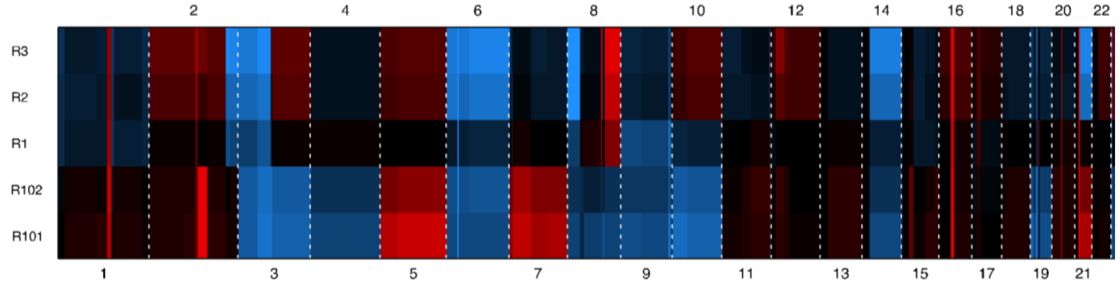

K114 (Left primary tumour: R1 – R4; Right primary tumour: R101 – R103)

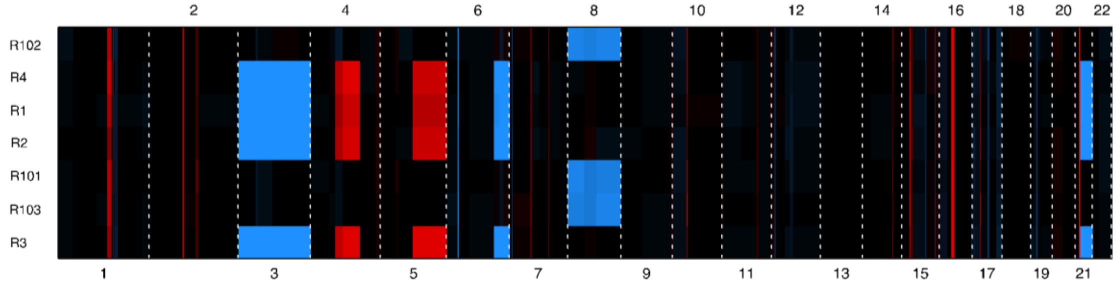

.
